# Interactions of *Entamoeba histolytica* peroxiredoxin with the TLR4/MD2 complex trigger ferroptosis in host cells

**DOI:** 10.1128/spectrum.00434-26

**Published:** 2026-07-20

**Authors:** Ruixue Zhou, Wenjie Li, Qingtong Zhou, Yanqing Zhao, Xia Li, Hongze Zhang, Dai Dong, Wenwen Jing, Hiroshi Tachibana, Meng Feng, Gang Fu, Xunjia Cheng

**Affiliations:** 1 Department of Medical Microbiology and Parasitology, School of Basic Medical Sciences, Fudan University 58305, Shanghai, China; 2 Department of Pharmacology, School of Basic Medical Sciences, Fudan University 58305, Shanghai, China; 3 Affiliated Hospital of Guizhou Medical University 74720, Guiyang, Guizhou, China; 4 Department of Infectious Diseases, Tokai University School of Medicine 314462, Isehara, Kanagawa, Japan; 5 Multiscale Research Institute of Complex Systems, Fudan University 12478, Shanghai, China; 6 Structural Biology Core Facility, Shenzhen Medical Academy of Research and Translation (SMART) 669132, Shenzhen, Guangdong, China; University of Huddersfield, Huddersfield, United Kingdom

**Keywords:** *Entamoeba histolytica*, peroxiredoxin, cryo-EM structure, TLR4, MD2, ferroptosis

## Abstract

Amoebiasis is a critical global parasitic disease caused by *Entamoeba histolytica*. It includes amoebic colitis and extraintestinal abscesses. *E. histolytica* peroxiredoxin (*Eh*Prx) is a crucial antioxidant enzyme that maintains redox homeostasis and participates in counteracting oxidative stress, supporting trophozoite survival and pathogenicity in the host. We observed the *Eh*Prx structure to be a decameric ring formed through the polymerization of five dimers. *Eh*Prx had a unique α-helical structure at its N-terminus. Protein-protein docking and surface plasmon resonance (SPR) assay results suggest that *Eh*Prx may bind to myeloid differentiation factor 2 (MD2), the ligand protein of toll-like receptor 4 (TLR4), rather than directly to TLR4. Furthermore, *Eh*Prx was crucial in the induction of cellular ferroptosis by *E. histolytica* trophozoites and regulated ferroptosis in host cells through the TLR4/MD2 pathway. These findings may hold a solid structural basis and promising concepts for investigating the pathogenic mechanisms and drug targets of *E. histolytica*.

**IMPORTANCE:**

*Entamoeba histolytica* is the causative agent of amoebiasis, a significant global health concern. Within this parasite, *E. histolytica* peroxiredoxin (*Eh*Prx) plays a pivotal role in mitigating oxidative stress, a critical survival mechanism in the hostile environment of the human host. *Eh*Prx belongs to a family of antioxidant enzymes responsible for detoxifying peroxides, which are deleterious byproducts of cellular metabolism and host-derived immune responses. By neutralizing reactive oxygen species (ROS), EhPrx safeguards the parasite's cellular integrity, ensuring its survival, proliferation, and pathogenicity. Despite its functional importance, the precise structural details of *Eh*Prx remain elusive, hindering a comprehensive understanding of its molecular mechanisms. Cryo-electron microscopy (cryo-EM) offers a promising avenue for elucidating the high-resolution structure of *Eh*Prx, which could reveal critical insights into its biological functions and inter- or intramolecular interactions. Such structural characterization may be indispensable for advancing our knowledge of *E. histolytica* biology and identifying novel diagnostic markers to combat amoebiasis effectively.

## INTRODUCTION

*Entamoeba histolytica* causes human amoebiasis, an enteric disease that affects millions worldwide (1) and represents a significant public health challenge in developing countries (2). *E. histolytica* is capable of becoming virulent, causing harm to the host and acting as the causative agent of amoebiasis, dysentery, and liver abscesses (3). There is currently no commercially available vaccine against amoebiasis. The mainstay of treatment is metronidazole, though its use in humans is associated with some adverse effects (4, 5). Identifying the factors that determine the outcome of *E. histolytica* infections and elucidating the nature of amoeba virulence are critical challenges in amoebiasis research, representing a long-standing and fundamental question in the study of pathogenicity. Addressing these challenges is essential for advancing the development of effective therapeutics and vaccines.

*E. histolytica*, a microaerophilic unicellular organism, thrives in low-oxygen environments. However, during its growth, it is exposed to reactive oxygen species (ROS), which induce oxidative stress (6). This oxidative stress is intensified by the activation of the host immune response (7). *E. histolytica* lacks key components of canonical eukaryotic anti-oxidative defense systems, such as peroxidase, glutathione, catalase, and glutathione-recycling enzymes (glutathione peroxidase and glutathione reductase) (8); however, the genome of *E. histolytica* encodes several proteins, such as peroxiredoxin (Prx), flavoprotein A, superoxide dismutase, ferredoxin, thioredoxin, and thioredoxin reductase, for detoxification of ROS (9–11). *E. histolytica* peroxiredoxin (*Eh*Prx) is a 29-kDa thiol-rich peripheral membrane protein with peroxidase activity (12) and a marker of pathogenic isolates (13). It is believed to be crucial in protecting organisms against H_2_O_2_ generated by internal and environmental oxidative stress (14). Inhibition of *Eh*Prx gene expression by antisense RNA technology has shown approximately 55% inhibition in *Eh*Prx expression, maximum ROS accumulation, and significantly lower viability in *Eh*Prx downregulated trophozoites during oxidative stress (15). *Eh*Prx, an antioxidant enzyme essential for the parasite’s survival during host tissue invasion, may induce autophagy and exhibit cytotoxic effects in macrophages (16). Notably, single-cell RNA sequencing and trophozoite expression profile analysis revealed that *Eh*Prx expression was markedly upregulated in trophozoites co-incubated with Chinese hamster ovary cells compared to those cultured in isolation (NCBI BioProject accession number: PRJNA680388) (17). Similarly, other studies showed that *Eh*Prx was overexpressed in different specimens of patients with amoebic liver abscess and specimens of genital amoebiasis by reverse transcription-quantitative polymerase chain reaction (RT-qPCR) (18); and levels increased in human intestinal xenografts at the 24 h time point (19). A previous laboratory study revealed that *Eh*Prx triggered toll-like receptor 4 (TLR4)-dependent activation of the NLRP3 inflammasome in a manner independent of cell-to-cell contact (20). Thus, *Eh*Prx has great potential to function as a virulence factor during *E. histolytica* infection of the host.

In this study, we used cryo-electron microscopy (cryo-EM) to analyze the structure of *Eh*Prx, purified from *E. histolytica* trophozoites, and investigate its role in regulating the pathogenicity of *E. histolytica* in host cells. This research is crucial for elucidating the mechanisms underlying *E. histolytica* pathogenicity.

## RESULTS

### Cryo-EM structure of *Eh*Prx

**Fig 1:**
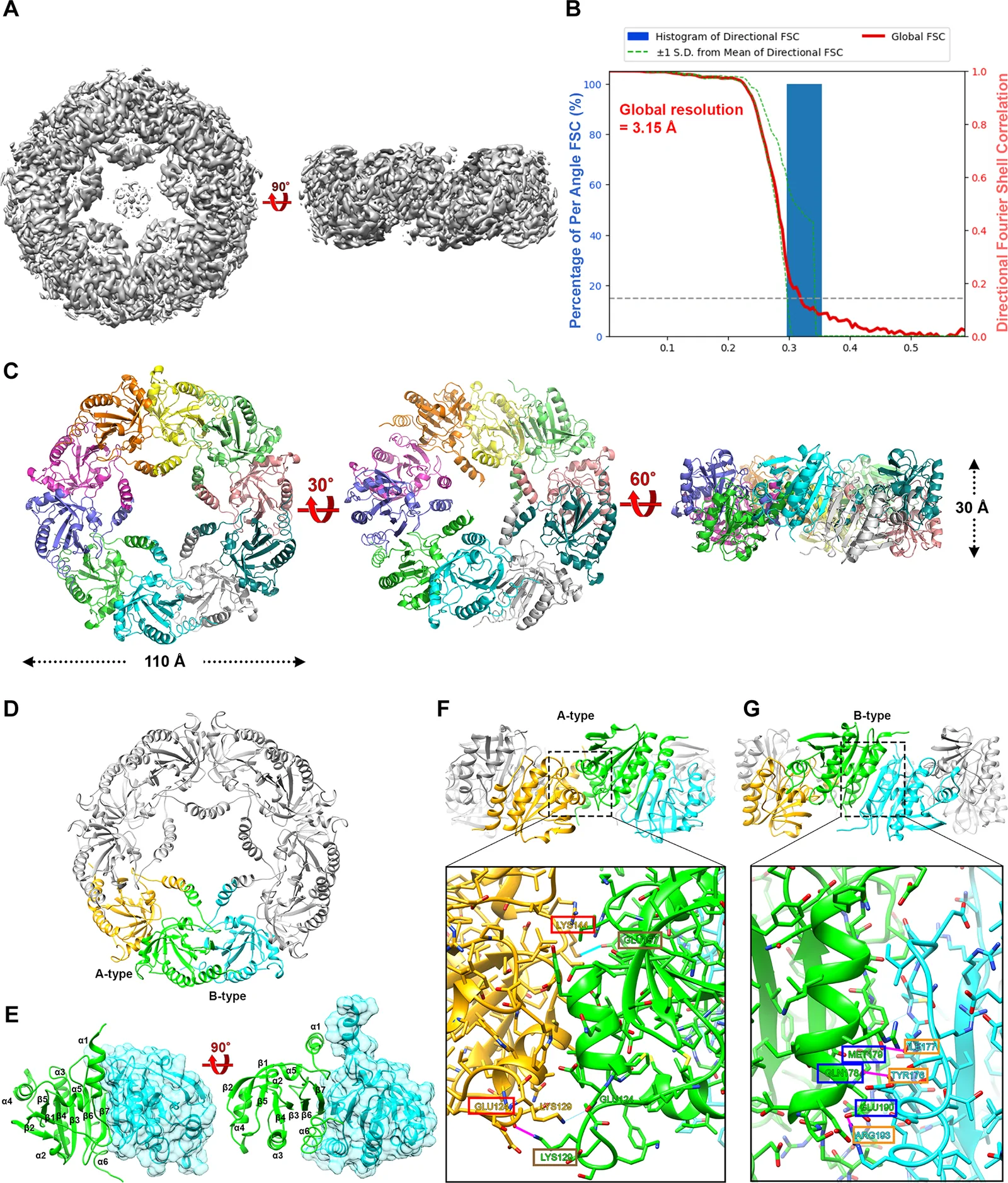
Cryo-EM structure of *Entamoeba histolytica* peroxiredoxin (*Eh*Prx). (**A**) Electron density map of the *Eh*Prx ring structure obtained by three-dimensional reconstruction. (**B**) FSC (=0.143) curve with an overall resolution of 3.15 Å. (**C**) *Eh*Prx decameric ring atomistic model at different angles. (**D**) Three of the monomers were selected for analysis of the interactions between A-type (orange and green) and B-type (green and cyan) dimers. (**E**) The *Eh*Prx monomer comprises six α-helices and seven β-folds. The monomers indicated by the green banded schematic are labeled with α-helical and β-folded compositions, and the subunits of the monomers adjacent to them are represented by the SURFACE model. (**F, G**) Close-up views of the A-type (**F**) and B-type (**G**) dimers show amino acid side chains within the corresponding dashed boxes. Residues involved in intermolecular hydrogen bonds (magenta lines) and salt bridges (cyan lines) are labeled. In the A-type dimer, GLU124 and LYS144 from the left monomer are highlighted in red boxes; LYS129 and GLU157 from the right monomer are marked in brown boxes. In the B-type dimer, GLU190, GLN178, and MET179 from the left monomer are shown in blue boxes; ARG193, TYR176, and ILE177 from the right monomer are indicated in orange boxes.

Confocal microscopy was performed using a monoclonal antibody (4G6) (13, 21) that had been prepared in advance and exhibited highly specific reactivity with *Eh*Prx. The results of confocal microscopy observations revealed the presence of *Eh*Prx beneath the membrane of the *E. histolytica* trophozoites (Fig. S1A). Subsequently, the purification of *Eh*Prx from *E. histolytica* trophozoites utilizing the same 4G6 monoclonal antibody through immunoaffinity chromatography was achieved successfully (Fig. S1B through D). Direct electron detection imaging revealed that *Eh*Prx in solution aggregated to form well-defined, intact, and uniformly sized particles (Fig. S1E). Two-dimensional (2D) classifications revealed that *Eh*Prx polymerized to form doughnut-shaped oligomeric cyclic particles. These particles were uniformly distributed and randomly oriented in the top-bottom and side directions (Fig. S1F). An electron density map of the *Eh*Prx ring structure (Fig. 1A; Fig. S1G), obtained through 3D reconstruction with an overall resolution of 3.15 Å (Fig. 1B; Table S1), was used to build an atomistic model of the *Eh*Prx ring. The results indicated that the *Eh*Prx ring is a decameric complex formed by the polymerization of five dimers (Fig. 1C). Additionally, electron densities were observed in the center of the decameric ring, which may represent non-proteinaceous material or the highly flexible N-terminal portion of *Eh*Prx (amino acid residues 1–23).

The interactions between *Eh*Prx dimers included A-type and B-type (Fig. 1D). Each *Eh*Prx monomer comprised six α-helices and seven β-strands (Fig. 1E). The interactions between *Eh*Prx dimers primarily involved hydrophobic and polar interactions. In the A-type dimer, the GLU124 between α4 and β5 formed a hydrogen bond with LYS129 of the adjacent monomeric subunit. Additionally, the LYS144 on the loop between β5 and α6 formed a salt bridge with GLU157 of the neighboring monomeric subunit (Fig. 1F). In the B-type dimer, interactions were primarily mediated by the β7 and the region between β7 and α6. Hydrogen bonds were formed between GLU190, GLN178, and MET179 with ARG193, TYR176, and ILE177 of the neighboring monomeric subunits, respectively (Fig. 1G). Thus, the A-type and B-type dimers differ primarily in the residues and positions of their monomers involved in hydrogen bonding.

**Fig 2:**
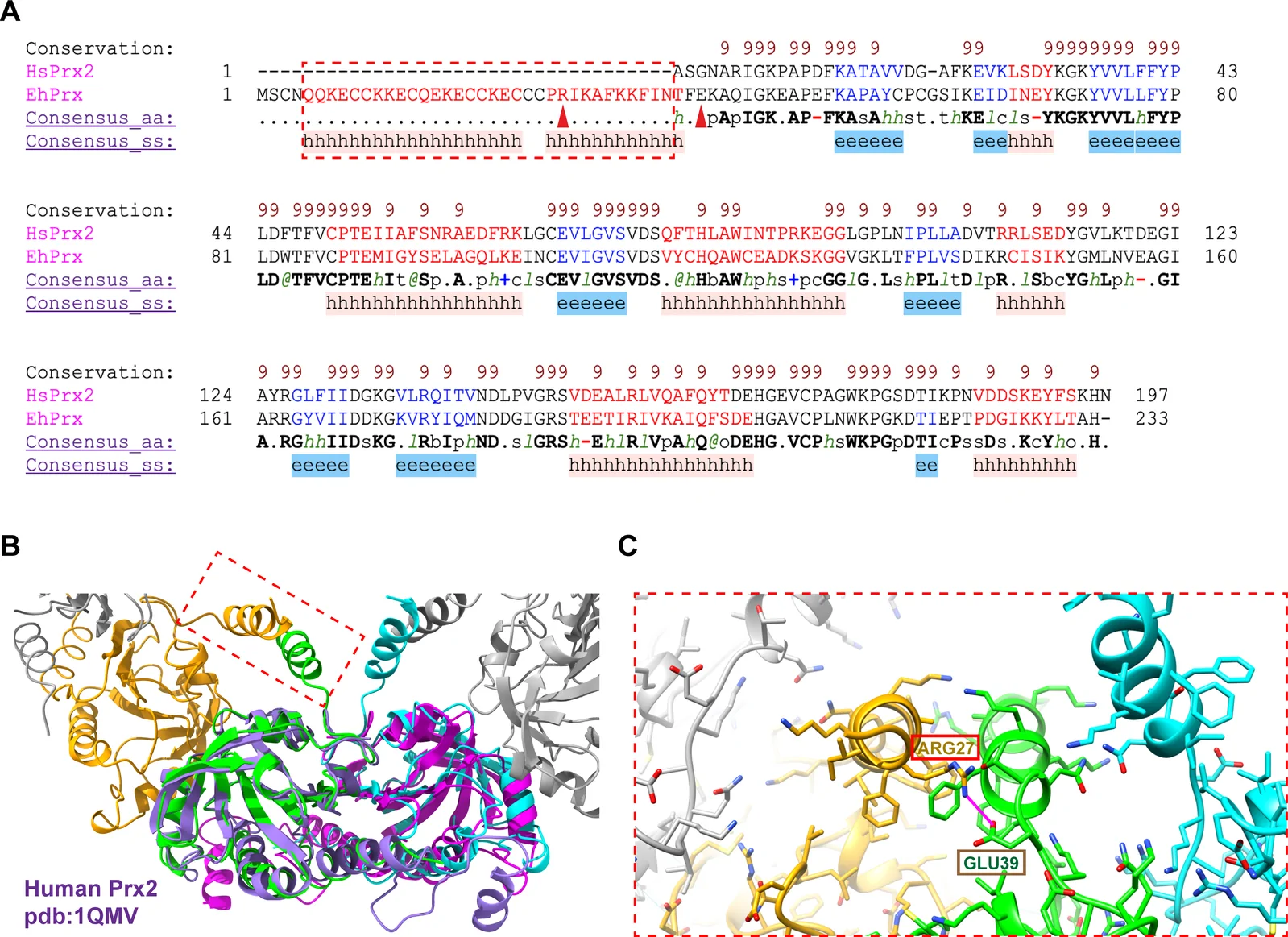
*Eh*Prx had a unique α-helical structure at the N-terminus. (**A**) Amino acid sequence comparison and secondary structure prediction of *Eh*Prx with human Prx2 (Uniprot code: P32119), where the red dashed box indicated the N-terminal sequence specific to *Eh*Prx, and the red arrowheads indicate residues ARG27 and GLU39 involved in hydrogen bond formation. (**B**) Structural comparison between *Eh*Prx and human Prx2 (PDB: 1QMV). The dimeric framework of human Prx2 is shown in medium purple and magenta. The red dashed box highlights the *Eh*Prx-specific α-helix structure. (**C**) Hydrogen bonding within the N-terminal α-helix of *Eh*Prx. ARG27 (brown box) from the left monomer forms a hydrogen bond (magenta line) with GLU39 (brown box) from the right monomer.

Human Prx2 is a 2-Cys Prx (22). Comparative analysis of the amino acid sequences and secondary structures of *Eh*Prx and human Prx2 (Uniprot: P32119) revealed that *Eh*Prx was well-conserved but possessed a distinct N-terminal α-helix (Fig. 2A). Structural alignment with the human Prx2 dimer (PDB: 1QMV) confirmed high conservation, yielding a Cα root-mean-square deviation of 1.31 Å. Notably, the *Eh*Prx-specific α-helix corresponded to the CYS24-GLU39 segment in the atomic model resolved in this study (Fig. 2B). Further analysis revealed that the ARG27 in the α1 helix formed a salt bridge with the GLU39 in the loop between α1 and β1, interacting with its monomer subunit (Fig. 2C). This α-helical interaction with the neighboring monomer subunit was not observed in the B-type dimer. We predicted that the α-helical structure in the *Eh*Prx decameric ring may serve to stabilize the complex and may also possess potential biological functions.

### Interaction between *Eh*Prx and TLR4/MD2

**Fig 3:**
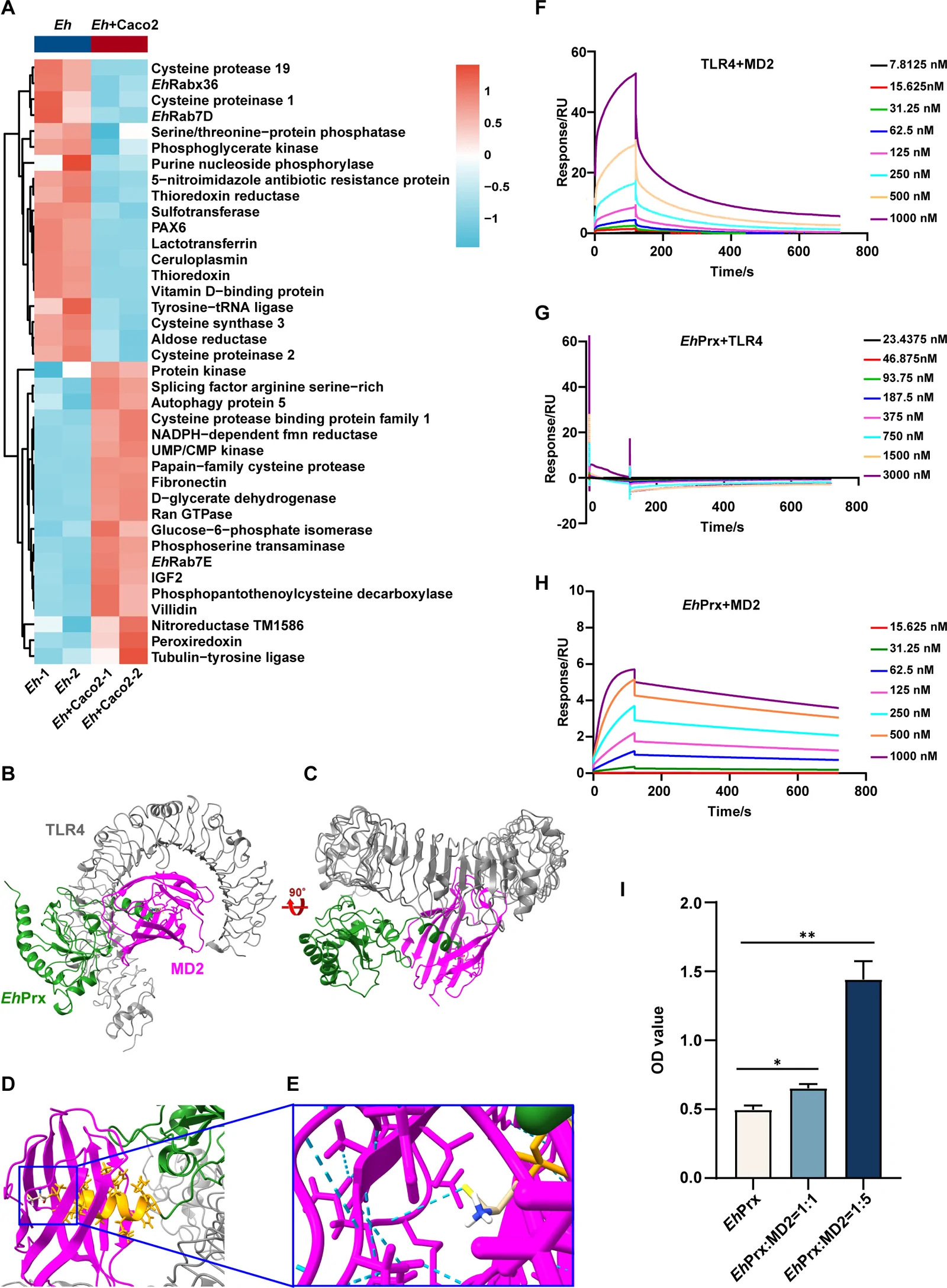
Interaction between *Eh*Prx and TLR4/MD2. (**A**) Quantitative mass spectrometry analysis was performed to assess changes in the levels of oxidative stress-related proteins released by *E. histolytica* trophozoites upon contact with Caco2 cells, including samples from the cell supernatants of *E. histolytica* alone (*Eh*) and co-incubation of *E. histolytica* with Caco2 (*Eh*+Caco2). Two independent biological replicates were performed for each condition, denoted as *Eh*-1, *Eh*-2, *Eh*+Caco2-1, and *Eh*+Caco2-2. (**B**) Confirmation of *Eh*Prx-TLR4/MD2 molecular docking, green for *Eh*Prx, gray for TLR4, and magenta for MD2. (**C**) The configuration of (**B**) was rotated through 90° in a forward direction. (**D**) The specific α-helix structure of *Eh*Prx is in orange. (**E**) The hydrogen bond of the α-helix of *Eh*Prx bound to MD2. (**F**) SPR assay was used to detect the interaction between TLR4 and MD2, with eight analytical concentrations set for TLR4. The kinetic parameters *K*_a_(1/Ms) = 2.367 × 10^4^, *K*_d_(1/s) = 3.581 × 10^−3^, and *K*_D_(M) = 1.512 × 10^−7^. (**G**) The interaction between *Eh*Prx and TLR4 was observed using a BIAcore instrument, but no binding was detected. (**H**) The SPR assay was employed to ascertain the interactions between *Eh*Prx with MD2, *K*_a_(1/Ms) = 3.608 × 10^4^, *K*_d_(1/s) = 5.594 × 10^−4^, *K*_D_(M) = 1.550 × 10^−8^. (**I**) Enzyme-linked immunosorbent assay (ELISA) results of *Eh*Prx interaction with MD2, with molar ratios of 1:1 and 1:5, respectively (data are presented as mean ± SEM; *n* = 3, *P < 0.05, **P < 0.01, by ordinary one-way ANOVA).

We employed quantitative mass spectrometry to analyze protein changes in the extracellular environment following the co-incubation of *E. histolytica* with Caco2 cells. Our results revealed alterations in the levels of oxidative stress-related proteins released by the trophozoites upon contact with Caco2 cells (Fig. 3A), notably an increased expression of *Eh*Prx (Fig. S2A and B). Previous research has shown that *Eh*Prx is overexpressed in extracellular vesicles (EVs) derived from *E. histolytica* trophozoites following coculture with human neutrophils (23). Consistent with this, we also detected *Eh*Prx in EVs derived from *E. histolytica* trophozoites (Fig. S2C). Based on these findings, we propose that *Eh*Prx is released through two potential mechanisms: via EVs or as a consequence of trophozoite rupture during host cell interaction.

Laboratory studies have demonstrated that Prx effectively activates NLRP3 (NOD-like receptor family pyrin domain containing 3) inflammatory vesicles and autophagy via TLR4 in *E. histolytica*. To investigate how *Eh*Prx interacts with TLR4, we used the protein-protein docking module of the Schrödinger 2021-2 software to predict the binding conformation of the monomeric *Eh*Prx to the TLR4/MD2 complex, with MD2 being a crucial co-receptor for TLR4 (24). The ternary TLR4-MD2-*Eh*Prx complex was initially modeled by first docking *Eh*Prx to MD2 to identify their optimal interface, followed by the alignment of the TLR4-MD2 complex structure with the docked MD2-*Eh*Prx unit. This approach preserved the physiological TLR4-MD2 interface, which maintains a stable, well-defined conformation as resolved by both cryo-EM and X-ray structures (25, 26), while positioning *Eh*Prx at MD2’s ligand-binding pocket. Three potential complex structures were generated using the Protein Preparation Wizard after energy minimization (Table S2). The best docking score model exhibited the highest level of structural stability, reflected in its PIPER (an FFT-based protein docking program with pairwise potentials [27]) pose energy (−906.8) and PIPER pose score (−468.7) (Fig. 3B). Structural analysis revealed that the interactions between *Eh*Prx and TLR4 were less robust than those between MD2 and TLR4 (Fig. 3C). The unique α-helix of *Eh*Prx was inserted into the MD2 structure (Fig. 3D), creating additional polar interactions, including hydrogen bonds and salt bridges (Fig. 3E). The molecular docking suggests that *Eh*Prx may bind to MD2 rather than TLR4 and potentially exert a biological role.

MD2, a ligand protein for TLR4 that recognizes pathogen-associated molecular patterns and activates intrinsic immunity (28), was a key cofactor in the activation of pro-inflammatory pathways (29). To investigate the interaction partner(s) of *Eh*Prx within host cells, we employed the label-free SPR assay using a BIAcore instrument. This analysis revealed that TLR4 and MD2 exhibited high affinity [*K*_D_(M) = 1.512 × 10^−7^] (Fig. 3F). However, *Eh*Prx proteins showed no interactions with TLR4 proteins (Fig. 3G). In contrast, the interaction between *Eh*Prx and MD2 was characterized by high affinity [*K*_D_(M) = 1.55 × 10^−8^] (Fig. 3H). Enzyme-linked immunosorbent assay (ELISA) demonstrated an elevated binding response at a 1:5 molar ratio of *Eh*Prx to MD2 (Fig. 3I). This experimental condition was employed solely to facilitate the detection of the binding signal and does not reflect the physiological stoichiometry *in vivo*. To validate *Eh*Prx binding to MD2, bacteria expressing recombinant Prx (rPrx) and mutant-rPrx (two mutations in the enzyme active site) were purified. SPR assay provided affinity constants of 1.022 × 10^−8^ and 9.73 × 10^−7^ M, respectively (Fig. S2D and E). These results indicate that natural *Eh*Prx purified from *E. histolytica* trophozoites and prokaryotically expressed rPrx proteins can bind to MD2. This interaction appears to be independent of the two enzyme activation sites of *Eh*Prx. Confocal microscopy revealed that when *E. histolytica* trophozoites were co-cultured with host cells, *Eh*Prx accumulated beneath the trophozoite membrane and colocalized with MD2 on the host cell surface (Fig. S3). These results suggest that *Eh*Prx may signal through MD2 on the host cell surface to facilitate interaction with TLR4.

### *Eh*Prx induced ferroptosis in Caco2 cells

**Fig 4:**
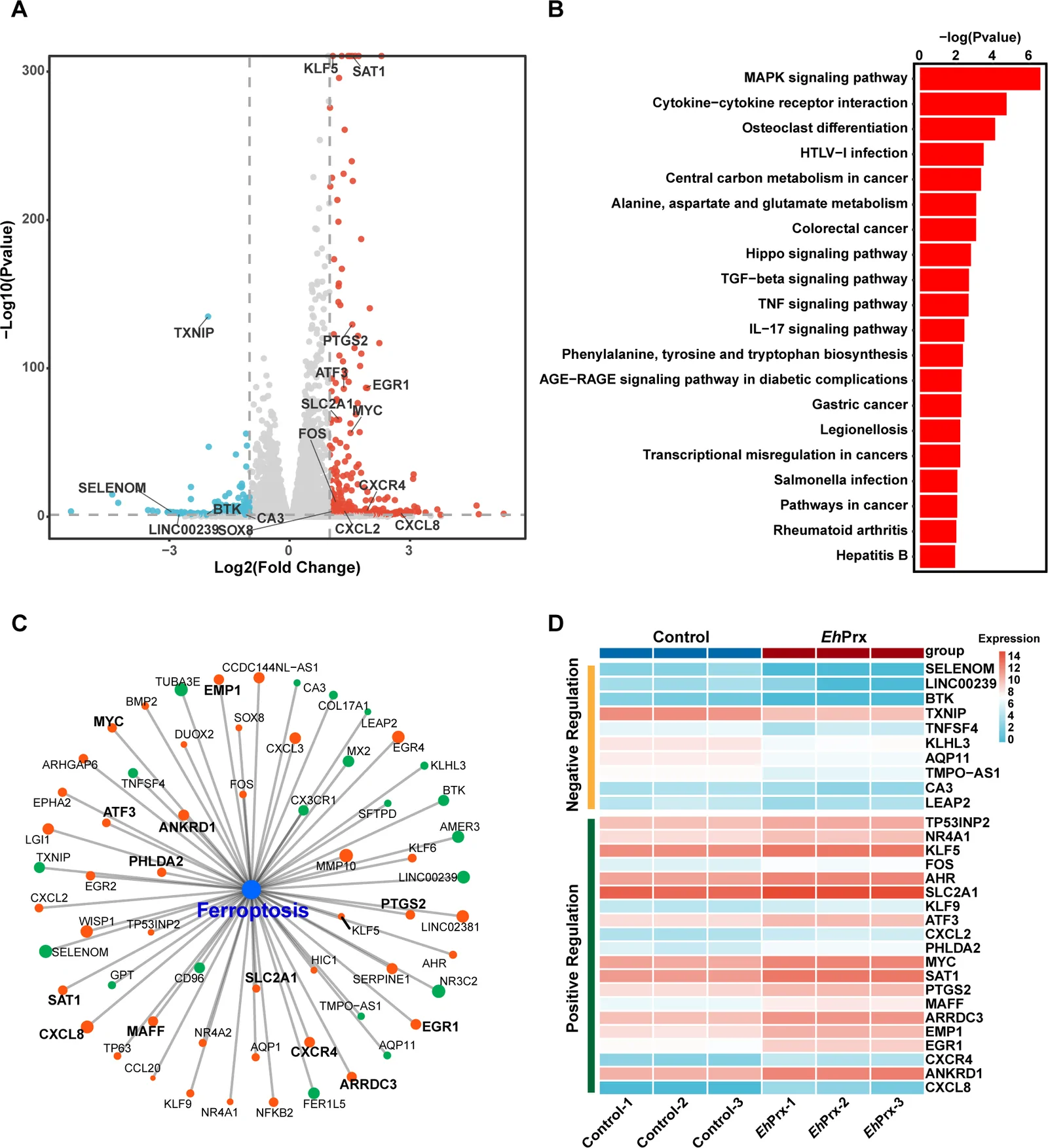
*Eh*Prx influenced the transcriptional profile of Caco2 cells. (**A**) Volcano plots of differentially expressed genes, blue dots indicate genes significantly downregulated in the *Eh*Prx/control groups, red dots indicate genes significantly upregulated in the *Eh*Prx/control group, and gray dots in the middle indicate non-significantly differing genes. (**B**) Significant enrichment of KEGG pathway histograms. (**C**) Diagram of ferroptosis regulators expression. The spot size corresponds to the Log2(Fold Change). Red and green colors denote upregulated and downregulated genes, respectively. Genes most frequently associated with ferroptosis in the literature are highlighted in bold. (**D**) Clustered heatmap of differentially expressed genes: each row represents one gene, and each column represents a sample. The color scale (right) denotes the expression levels of the significantly differentially expressed genes, where a gradient from red to blue represents high to low expression, respectively. Relative to the control group, a darker blue or lighter red color in the *Eh*Prx group is interpreted as negative gene regulation, whereas a lighter blue or darker red color indicates positive regulation.

To investigate the impact of *Eh*Prx on host cells, we used RNA-seq to analyze the transcriptional profiles of Caco2 cells following stimulation with *Eh*Prx (Fig. S4A and B). A total of 20,473 human genes were identified, among which 684 showed significant differential expression (*P* value ≤0.05), including 371 upregulated and 313 downregulated (Fig. 4A; Fig. S4C). Kyoto Encyclopedia of Genes and Genomes (KEGG)-enriched pathways included the mitogen-activated protein kinase (MAPK) signaling pathway, amino acid metabolism, and interleukin (IL)-17 signaling pathway, which are closely associated with the onset and regulation of ferroptosis (Fig. 4B). Ferroptosis is characterized by the abnormal accumulation of toxic lipid reactive oxygen clusters in the cell membrane system (30). Ferroptosis is regulated by multiple intracellular signaling pathways and antioxidant systems, including amino acid (31), iron (32), and lipid metabolism (33). Gene Ontology functional enrichment analysis revealed that the most significant biological process associated with ferroptosis was the “response to lipid.” The cellular composition most closely related to ferroptosis was the “plasma membrane.” The molecular functions of “DNA-binding transcription factor activities” and “signaling receptor binding” were involved in regulating ferroptosis (Fig. S4D). In addition, several genes associated with ferroptosis were identified among the differentially expressed genes (Fig. 4C and D). These suggest that *Eh*Prx stimulation of host cells may induce ferroptosis. Caco2 cells were stimulated with *Eh*Prx purified from *E. histolytica* trophozoites to investigate the role of *Eh*Prx in host cells. *Eh*Prx caused decreased mitochondrial membrane potential (Fig. S5A and B) and increased ROS (Fig. S5C) and Fe^2+^ (Fig. S5D) levels. These effects are vital biochemical indicators of ferroptosis in Caco2 cells. Additionally, exposure of Caco2 cells to *E. histolytica* trophozoites resulted in elevated intracellular levels of both ROS (Fig. S5E) and Fe^2+^ (Fig. S5F). Dinaciclib has been identified as a novel direct inhibitor of *Eh*Prx (34). Prior to co-incubation with Caco2 cells, *E. histolytica* trophozoites were treated with 10 μM Dinaciclib for 24 h. Under these conditions, inhibition of *Eh*Prx abrogated the elevation of intracellular ROS (Fig. S5G) and Fe^2+^ (Fig. S5H) levels. Notably, *Eh*Prx and *E. histolytica* trophozoites induce ferroptosis in host cells. This finding suggests that *Eh*Prx plays a critical role during the *E. histolytica* trophozoites’ challenge of host cells and contributes to the induction of cellular ferroptosis.

### *Eh*Prx induced ferroptosis in Caco2 cells via the TLR4/MD2 pathway

**Fig 5:**
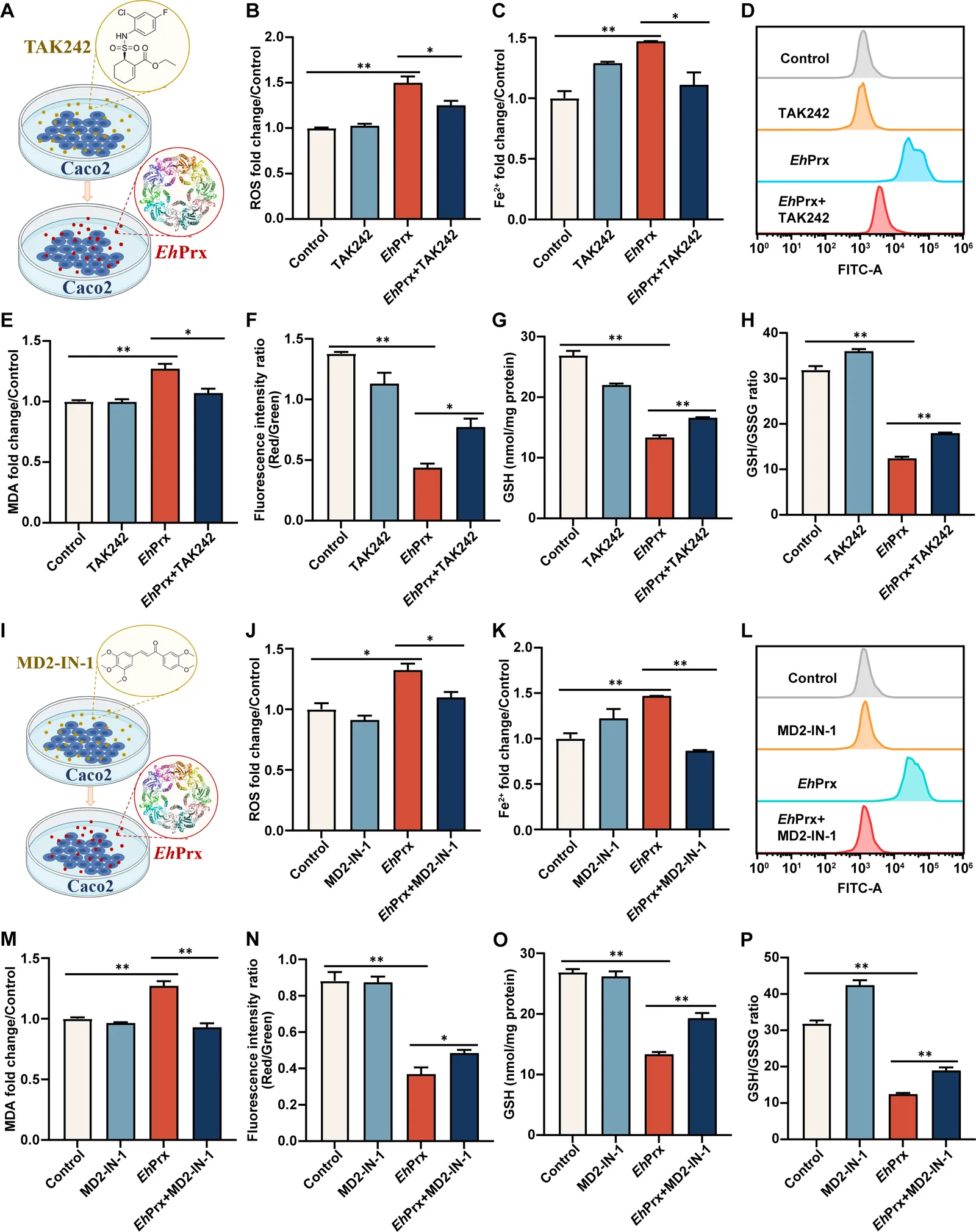
Biochemical indicators of ferroptosis in Caco2 cells stimulated with *Eh*Prx and TLR4/MD2 inhibitors. (**A**) Schematic diagram: Caco2 cells were stimulated with *Eh*Prx after being treated with TAK242 (TLR4 inhibitor). Detection of changes in ROS (**B**), Fe^2+^ (**C**), LPO (**D**), MDA (**E**), mitochondrial membrane potential changes (**F**), GSH content (**G**), and GSH/GSSG ratio (**H**) levels. Data are presented as mean ± SEM; *n* = 3, *P < 0.05, **P < 0.01, by ordinary one-way ANOVA. (**I**) Schematic diagram: Caco2 cells were stimulated with *Eh*Prx after being treated with MD2-IN-1 (MD2 inhibitor). Detection of changes in ROS (**J**), Fe^2+^ (**K**), LPO (**L**), MDA (**M**), mitochondrial membrane potential (**N**), GSH content (**O**), and GSH/GSSG ratio (**P**) levels. Data are presented as mean ± SEM; *n* = 3, *P < 0.05, **P < 0.01, by ordinary one-way ANOVA.

Caco2 cells were stimulated with *Eh*Prx after being treated with TAK242 (TLR4 inhibitor) (Fig. 5A). *Eh*Prx increased ROS (Fig. 5B), Fe^2+^ (Fig. 5C), lipid peroxide (Fig. 5D), and malondialdehyde (MDA) (Fig. 5E) levels in Caco2 cells, but these indices decreased after being treated with TAK242. *Eh*Prx treatment resulted in decreased mitochondrial membrane potential (Fig. 5F), GSH (reduced glutathione, GSH) content (Fig. 5G), and GSH/GSSG (oxidized glutathione, GSSG) ratio (Fig. 5H); but these indices increased after treatment with TAK242.

Similarly, Caco2 cells were stimulated with *Eh*Prx after being treated with MD2-IN-1 (MD2 inhibitor) (Fig. 5I). *Eh*Prx increased ROS (Fig. 5J), Fe^2+^ (Fig. 5K), lipid peroxide (Fig. 5L), and MDA (Fig. 5M); decreased mitochondrial membrane potential (Fig. 5N), GSH content (Fig. 5O), and GSH/GSSG ratio (Fig. 5P) levels via the MD2 in Caco2 cells.

**Fig 6:**
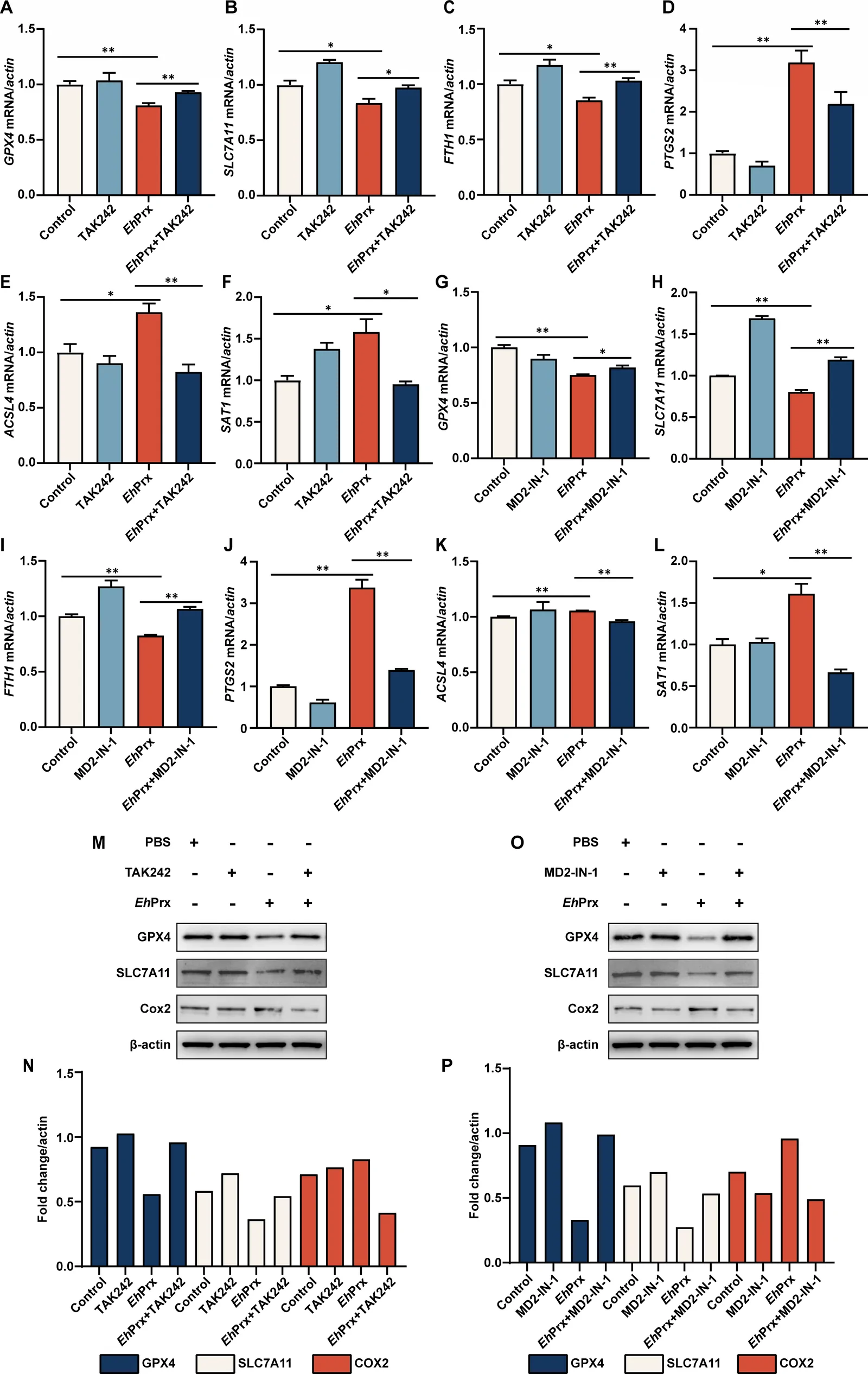
Transcriptional and protein expression of ferroptosis-related genes in Caco2 cells treated with *Eh*Prx and TLR4/MD2 inhibitors. (**A–F**) mRNA expression levels of ferroptosis-related genes—(**A**) *GPX4*, (**B**) *SLC7A11*, (**C**) *FTH1*, (**D**) *PTGS2*, (**E**) *ACSL4*, and (**F**) *SAT1*—in Caco2 cells stimulated with *Eh*Prx and TAK242, as determined by qPCR. Data are presented as mean ± SEM; *n* = 3; *P < 0.05, **P < 0.01 by ordinary one-way ANOVA. (**G–L**) mRNA expression levels of the same gene set in Caco2 cells treated with *Eh*Prx and MD2-IN-1, measured by qPCR. Data are presented as mean ± SEM; *n* = 3; *P < 0.05, **P < 0.01 by ordinary one-way ANOVA. (**M**) Representative western blotting images of ferroptosis-associated proteins GPX4, SLC7A11, and COX2 (also known as PTGS2) in cells treated with *Eh*Prx and TAK242. (**N**) Densitometric quantification of the protein bands shown in panel **M**. (**O**) Representative western blotting images of GPX4, SLC7A11, and COX2 in cells treated with *Eh*Prx and MD2-IN-1. (**P**) Densitometric quantification of the protein bands shown in (**O**).

These changes are significant biochemical indicators of ferroptosis. At the gene level, ferroptosis-related genes showed alterations upon stimulation with *Eh*Prx. *Eh*Prx regulated ferroptosis genes *GPX4* (Fig. 6A and G), *SLC7A11* (Fig. 6B and H), and *FTH1* (Fig. 6C and I) through TLR4 and MD2, resulting in decreased transcription, whereas the transcription levels of *PTGS2* (Fig. 6D and J), *ACSL4* (Fig. 6E and K), and *SAT1* (Fig. 6F and L) were increased. At the protein level, the expression of ferroptosis-related proteins was altered in *Eh*Prx-stimulated Caco2 cells. *Eh*Prx decreased the expression levels of GPX4 and SLC7A11 proteins while increasing COX2 (PTGS2) expression through TLR4 (Fig. 6M and N) and MD2 (Fig. 6O and P). These indicate that *Eh*Prx induces changes in the expression of host cell ferroptosis-related genes via the TLR4/MD2 pathway.

## DISCUSSION

When *E. histolytica* invades the host tissues, injured intestinal epithelial cells release potent chemokines, recruiting immune cells to the site of infection (35, 36). Upon activation, these immune cells produce ROS and nitric oxide to attack the trophozoites (37). Furthermore, *E. histolytica* infection triggers excessive ROS production in the host, underscoring the complex interplay between parasitic defense mechanisms and host oxidative responses (38). In this oxidative environment, the parasite maintains intracellular hypoxia as a crucial survival strategy (39). *E. histolytica* adapts to oxidative stress through an array of encoded detoxification systems, including L-cysteine, flavodiiron proteins, peroxiredoxin, and trichostatin A (40–44). Among these, *Eh*Prx—a key enzyme in oxygen metabolism—is highly expressed in the HM-1: IMSS strain (45). During tissue adherence and invasion, *Eh*Prx has been shown to interact with the cytosolic domain of the surface Gal/GalNAc lectin, initiating signal transduction that protects the parasite from oxidative attack by activated host cells (46).

*Eh*Prx represents a typical 2-Cys Prx with over 20 transcripts, as indicated in the Entamoeba database (http://amoebadb.org). The fundamental structural units of the *Eh*Prx decameric ring are asymmetric homodimers formed by the monomeric subunits of the *Eh*Prx protein, similar to the decameric rings in other Prx species (47). However, *Eh*Prx features a unique α-helical structure at its N-terminus. Numerous studies have characterized the functional roles of the N-terminal extensions in Prx proteins. The Prx4 from the large yellow croaker *Pseudosciaena crocea* is a typical 2-Cys Prx with an N-terminal antiparallel β-sheet that contributes to the dimer interface. Deletion of this β-sheet decreases the *in vitro* peroxidase activity to approximately 50% of that of the wild type (48). Comparative studies of Prx proteins in *E. histolytica*, *Entamoeba dispar*, and *Entamoeba moshkovskii* revealed conserved active-site architectures but divergent N-terminal domains. These structural variations, particularly in the N-terminal extension length, correspond to marked differences in Prx catalytic efficiency across species (49). The potential biological function of this special α-helical structure may involve stabilizing the polymerization of the A-type dimer or regulating the enzymatic activity of Prx.

Cryo-EM structural analysis revealed that the N-terminal α-helix of *EhPrx* has a relatively independent structure. Previous studies have indicated that Prx has biological functions that extend beyond its canonical antioxidant activity. In acute respiratory distress syndrome, elevated PRDX6 exacerbated lung injury by binding MD2 to activate the TLR4/NF-κB pathway, promoting M1 macrophage polarization (50). A tumor microenvironment study demonstrated that Prx1 activated macrophage TLR4 in an MD2-dependent manner, triggering proinflammatory cytokine secretion (TNF-α and IL-6) (51). The present study demonstrates that *Eh*Prx mediates the regulation of host cell immune responses through the TLR4/MD2 pathway during challenge by *E. histolytica*. Protein-protein docking demonstrated that the special α-helix of *Eh*Prx fused into the MD2 structure, forming additional polar interactions, such as hydrogen bonds and salt bridges. This suggests that *Eh*Prx may bind to MD2 rather than TLR4, thereby exerting its biological function. SPR assay also revealed that *Eh*Prx did not interact with TLR4 but with MD2 with high affinity. The redistribution of *Eh*Prx to a submembrane location during host cell challenge by *E. histolytica* trophozoites suggests an immunomodulatory role distinct from its canonical antioxidant function. *Eh*Prx appears to regulate host cell signaling pathways through MD2 interactions and subsequent TLR4 activation.

Emerging evidence indicates that *E. histolytica* employs trogocytosis as a distinct mechanism to kill human cells (52). Interestingly, the choice between trogocytosis and phagocytosis for engulfing host cells appears to depend on host cell size (53). Another well-established mechanism in *E. histolytica* pathogenesis involves the induction of programmed cell death or apoptosis (54, 55). More recently, ferroptosis—a novel form of regulated cell death driven by lethal lipid peroxidation—has been implicated in amebic pathogenesis. This process is characterized by increased ROS production, compromised antioxidant capacity, and elevated intracellular iron concentrations (56, 57). The activation of TLR4 by pathogen- or damage-associated molecular patterns initiates downstream signaling that regulates inflammatory and immune responses, including mechanisms interconnected with ferroptosis. Transcriptional profiling data suggested that *Eh*Prx markedly affects host cells by regulating several pathways, including the MAPK signaling pathway, amino acid metabolism, and the IL-17 signaling pathway, which are related to the occurrence and regulation of ferroptosis. Notably, ferroptosis is intricately regulated by numerous intracellular signaling pathways and antioxidant systems, including amino acid (58–60), iron (61, 62), and lipid (63–65) metabolisms. A distinctive feature of ferroptosis is the abnormal accumulation of toxic lipid ROS within the cellular membrane system (66). Therefore, during infestation, *E. histolytica* trophozoites express abundant Prx in response to the peroxides released from host cells. Some Prx may act on host cells, disrupting intracellular redox homeostasis, causing elevated ROS and Fe^2+^ levels, decreased mitochondrial membrane potential, and ferroptosis. The interaction between *Eh*Prx and the TLR4/MD2 complex led us to hypothesize that *Eh*Prx acts as a potential regulator of host cell ferroptosis. We propose that this may represent a common mechanism for inducing host cell ferroptosis in various pathogens. Confirmation of this pathway provides valuable insights into the pathogenic mechanisms of *E. histolytica*.

**Fig 7:**
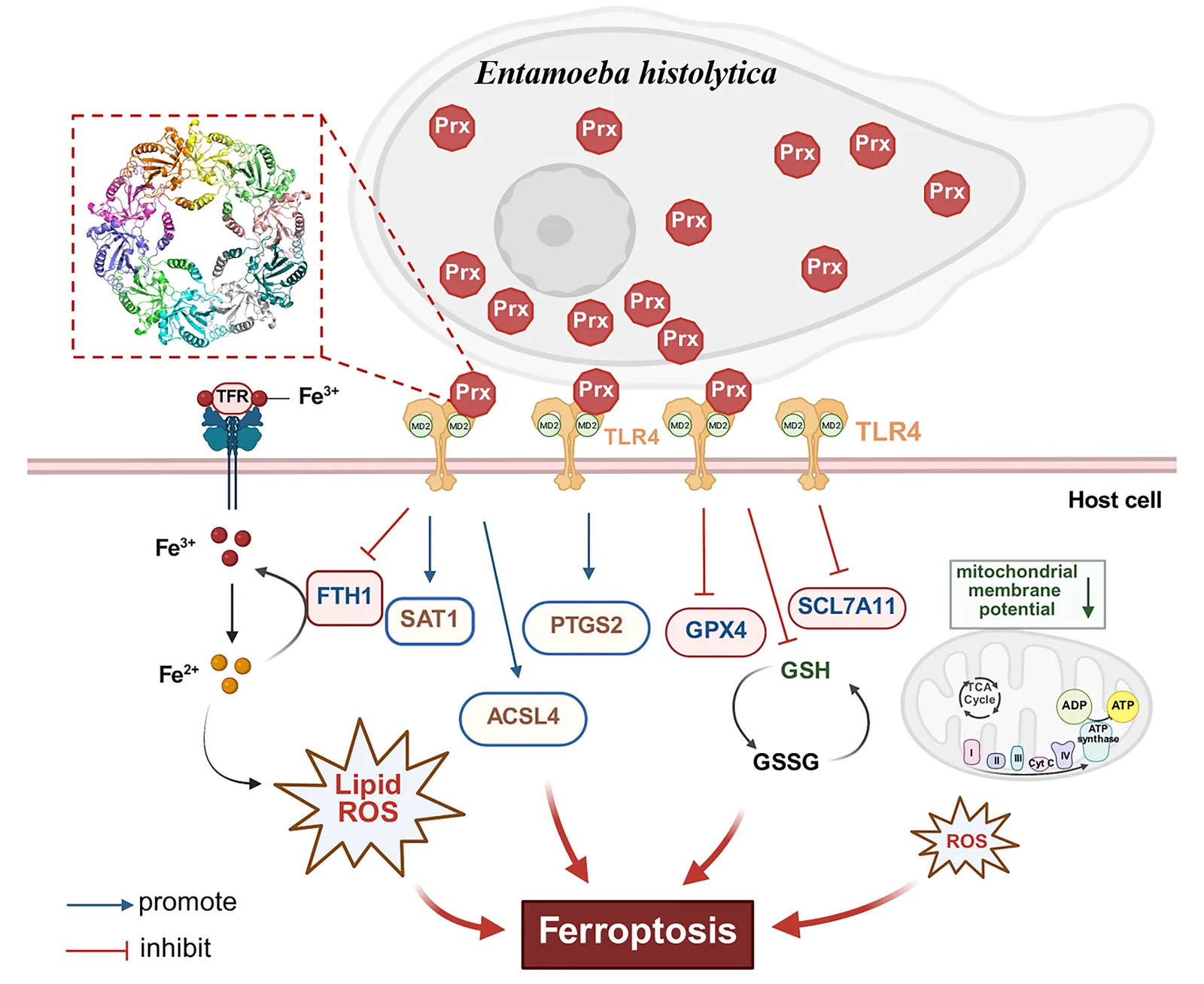
Schematic representation of the action of *Eh*Prx on host cells. It has been established that *Eh*Prx interacts with host cells by communicating with TLR4 using MD2 as an intermediate protein to activate the downstream ferroptosis signaling pathway. The ferroptosis-related genes act either synergistically or antagonistically, resulting in the accumulation of intracellular ROS and lipid peroxides, which ultimately leads to ferroptosis in the host cells.

In this study, we elucidated the structural architecture of *Eh*Prx and identified a distinctive N-terminal α-helix motif that may contribute to the stability of its multimeric assembly. We further demonstrated that *Eh*Prx facilitates activation of the TLR4/MD2 signaling pathway and promotes host cell ferroptosis (Fig. 7). These findings establish that Prx in *E. histolytica* serves dual physiological functions: as a crucial antioxidant protein essential for trophozoite survival, and as an immunomodulatory effector mediating host-pathogen interactions. This work provides mechanistic insights into a novel pathogenic strategy employed by *E. histolytica* and opens avenues for developing targeted anti-amoebic therapeutics by exploiting the unique structural features of *Eh*Prx.

## MATERIALS AND METHODS

### Cultivation of parasites and cells

Trophozoites of *E. histolytica* HM-1: IMSS were axenically cultured in a BI-S-33 medium supplemented with 10% (vol vol^−1^) heat-inactivated adult bovine serum (Sigma-Aldrich, St. Louis, Missouri, USA) at 36.5°C. Human colon carcinoma cells (Caco2 cells) were maintained in Eagle’s minimum essential medium (MEM) (Corning, Corning City, New York, USA) supplemented with 20% heat-inactivated fetal bovine serum (FBS) (HyClone Laboratories, Logan, Utah, USA), 100 units mL^−1^ penicillin, 100 μg mL^−1^ streptomycin, 2 mM L-glutamine (Gibco, San Diego, California, USA), MEM nonessential amino acids (Gibco, San Diego, California, USA), and 1 mM sodium pyruvate (Gibco, San Diego, California, USA). Cells were seeded into flasks containing a supplemented culture medium and maintained at 37°C in a humidified atmosphere of 5% CO_2_. The culture medium was changed every other day to eliminate non-adherent and dead cells. In addition, to reduce analytical interference and provide more reproducible experimental conditions, cell culture plates containing FBS were replaced with FBS-free medium in the assays. Trophozoites and cells were harvested in the logarithmic phase of growth and were used in the subsequent experiments.

### Purification of *Eh*Prx

The trophozoites of *E. histolytica* in the logarithmic growth phase were used for *Eh*Prx protein purification. An aliquot of 7.5 mg of 4G6 monoclonal antibody (13, 67) for *Eh*Prx was coupled with pretreated cyanogen bromide-activated agarose gel (Pharmacia Biotech, USA). Trophozoites were resuspended with a solubilization buffer following sonication, centrifugation, and filtration. Subsequently, the trophozoite supernatant was collected and purified using affinity chromatography with 4G6 monoclonal antibody following the manufacturer’s instructions. After dialysis with PBS, the protein solution was immediately filtered through a 0.22-μm filter for sterilization.

### Cryo-EM sample preparation

For cryo-EM grid preparation, 4 µL of *Eh*Prx protein (1.5 mg mL^−1^) was applied to gold grids (holey Quantifoil R1.2/1.3, 300 mesh) that were freshly plasma-cleaned using a Gatan 950 Solarus plasma cleaning system operating at 5 W power in the H_2_/O_2_ mixture for 20 s. The protein was incubated at 4°C and 100% humidity for 5 s. The grids were blotted for 3.5 s with a −3 blot force using a Vitrobot Mark IV (Thermo Fisher Scientific, Waltham, Massachusetts, USA), and samples were plunge-frozen in liquid ethane and stored in liquid nitrogen until use (68, 69).

### Cryo-EM data collection and image processing

Cryo-EM data were collected on a Titan Krios (Thermo Fisher Scientific, Waltham, Massachusetts, USA) transmission electron microscope operated at 300 kV. EPU software (Thermo Fisher Scientific, Waltham, Massachusetts, USA) was used to automatically capture the movie stacks with a K3 camera (Gatan, Pleasanton, California, USA) and a BioQuantum energy filter (Gatan, Pleasanton, California, USA) at a magnification of 105k× in super-resolution mode, resulting in a physical pixel size of 0.85 Å. Defocus ranges were set from −1.0 to −2.0 μm, and each movie stack (40 frames) was exposed to a dose of 50 e^−^ Å^−2^. In total, 7,743 movie stacks were imported into CryoSparc for analysis, including motion correction using MotionCor2 (70) and contrast transfer function correction. After inspection, 749,813 particles were extracted at a box size of 256 pixels from 7,444 micrographs. A non-uniform refinement was performed with a C5 symmetry, yielding a map at the resolution of 3.15 Å, which was estimated based on the gold-standard Fourier shell correlation 0.143 criterion. To build the *Eh*Prx model, a homologous model was generated using Swiss-Model (71), which was fitted into the cryo-EM using UCSF Chimera (72) and manually adjusted with COOT (73). Phenix (74) was used to perform a real-space refinement. UCSF Chimera was further used to generate the figures.

### Quantitative mass spectrometry and western blotting analysis of *E. histolytica* trophozoite proteins in the supernatants

The trophozoites of *E. histolytica* in the logarithmic growth phase were transfected into 6-well plates, with 3 × 10^4^ cells per well, and incubated at 36.5°C for 24 h in a microaerobic environment. Subsequently, the experimental group was supplemented with Caco2 cells at a density of 3 × 10^5^ cells per well in serum-free BI-S-33 medium. After a microaerobic incubation period of 3 h, the cell supernatants were extracted, and proteins were precipitated using cold acetone. The same volume of ultra-pure water was added to solubilize the proteins of the control group (*Eh*) or the experimental group (*Eh*+Caco2), followed by protein quantification using quantitative mass spectrometry with the Thermo Scientific Orbitrap Exploris 480 mass spectrometer.

Western blotting analysis was performed to detect the content of *Eh*Prx. The extracted protein was resuspended in 30 μL of loading buffer (containing β-ME) for subsequent detection by western blotting. 4G6 monoclonal antibody and goat anti-mouse IgG (H+L) (Abcam, UK) were used as the primary and secondary antibodies, respectively.

### Western blotting analysis of *Eh*Prx in EVs derived from *E. histolytica* trophozoites

For the isolation of amoebic EVs, 2 × 10^6^ *E. histolytica* trophozoites were seeded into T25 culture flasks and maintained under standard growth conditions. After attachment of the parasites, the flasks were washed twice with 2% glucose-PBS at 37°C, and 5 mL of serum-free BI-S-33 medium was added. Anaerobic incubation was then carried out for 3 h at 36.5°C. The culture supernatant was carefully collected, centrifuged at 500 × *g* for 5 min, and filtered through a 0.22-μm filter. EVs were purified with a Total Exosome Isolation Reagent (4478359; Invitrogen, Carlsbad, CA, USA) following the manufacturer’s instructions. After the addition of the isolation reagent, the mixture was incubated overnight at 4°C and subsequently centrifuged at 10,000 × *g* for 1 h to pellet the EVs. Finally, the EV pellets were resuspended in PBS for further analyses.

For *Eh*Prx identification in the EVs, western blotting was performed using a 4G6 monoclonal antibody as the primary antibody and a goat anti-mouse IgG (H+L) (Abcam, UK) as the secondary antibody.

### SPR assay

BIAcore T200 instruments (Cytiva, Wilmington, Delaware, USA) were used to evaluate the binding affinity of compounds to Prx via SPR, as previously described (75). CM5 Sensor Chip (Cytiva, Wilmington, Delaware, USA) was activated with a 7-min injection of the mixture of 50 mM N-hydroxy-succinimide and 200 mM 1-ethyl-3-(3-dimethylaminopropyl) carbodiimide. Subsequently, Prx was immobilized on the surface of the CM5 chip using an amine-coupling approach at a flow rate of 10 μL min^−1^ in 10 mM sodium acetate buffer (pH 5.0). Next, 20 μg mL^−1^ Prx was injected to reach the target level of 12,000 RU, and the surface was blocked with 1 M ethanolamine, pH 8.5. Series concentrations of MD2 were injected into the flow system and analyzed for 120 s, and the dissociation was set to 600 s. The surface was regenerated with Glycine-HCl (pH 2.0) for 30 s between different analytes. The binding affinity was determined by fitting to a Langmuir 1:1 binding model within the BIAcore Evaluation software (GE Healthcare, Chicago, Illinois, USA).

### Detection of the binding between *Eh*Prx and MD2 by ELISA

All analytical measurements were performed in 96-well plates. The plates were coated with 100 μL of *Eh*Prx (20 μg mL⁻¹) and incubated overnight at 4°C. After blocking, MD2 protein (Abcam, Cambridge, UK) was added at molar ratios of 1:1 and 1:5 (*Eh*Prx:MD2) and incubated for 2 h at room temperature. Subsequently, 100 μL per well of Rabbit anti-MD2 antibody (Abcam, Cambridge, UK) was added and incubated for 1 h at room temperature. The plates were then treated with a secondary antibody, Goat anti-Rabbit IgG H&L-HRP (Abcam, Cambridge, UK), for 1 h at room temperature. Finally, 100 μL of chromogenic solution was added to each well. Following a 20-min incubation and reaction termination, the absorbance was measured at 490 nm. Wells with *Eh*Prx alone served as the negative control.

### Detection of *Eh*Prx localization in trophozoites by laser confocal microscopy

A total of 10^6^ Caco2 or RAW264.7 cells were cultured in a 35 mm cell culture dish (Corning, Corning city, New York, USA) with sterilized cover slides (22 × 22 mm). Once the cells completely adhered to the slide, 5 × 10^5^ trophozoites were added to the culture dish. After incubating at 36.5°C for 1 h, the coverslips were fixed with 4% paraformaldehyde for 30 min and incubated with 0.01% Triton X-100. After blocking, the coverslips were incubated with Mouse 4G6 monoclonal antibody and Rabbit anti-MD2 (Abcam, Cambridge, UK) mixture, followed by Alexa Fluor Goat anti-Mouse-594 and Goat anti-Rabbit-488 (Invitrogen, Carlsbad, California, USA). Slides were observed under a laser confocal microscope (Leica, Wetzlar, Germany).

### Biochemical indicators for detecting ferroptosis

Caco2 cells were transfected into 6-well plates with 3.5 × 10^5^ cells per well, with the number of cells and well plates calculated according to the experimental requirements. The cells were cultured in a cell culture incubator at 37°C and 5% CO_2_. The TLR4 (TAK242, MedChemExpress, New Jersey City, New Jersey, USA) and MD2 (MD2-IN-1, MedChemExpress, New Jersey City, New Jersey, USA) inhibitors were added at a working concentration of 5 μmol L^−1^ (5 μmol L^−1^ DMSO as the control group), and the Caco2 cells were treated at 37°C for 1 h. Subsequently, the culture supernatant was removed, and the cells were washed twice with PBS. Next, the *Eh*Prx protein, diluted in serum-free MEM medium, was added to achieve a working concentration of 2.5 μg mL^−1^. The incubation and treatment continued for 6 h. The kits for detecting ferroptosis included FerroOrange Fluorescent Probe Assay Kits (Dojindo, Kumamoto Prefecture, Japan), ROS Assay Kit -Highly Sensitive DCFH-DA (Dojindo, Kumamoto Prefecture, Japan), Liperfluo Assay Kit (Dojindo, Kumamoto Prefecture, Japan), MDA Assay Kit (Dojindo, Kumamoto Prefecture, Japan), and GSSG/GSH Quantification Kit II (Dojindo, Kumamoto Prefecture, Japan). The experiment was conducted following the instructions provided in the kits.

### Quantitative real-time polymerase chain reaction analysis

Total RNA was extracted using the RNeasy Plus Mini kit (Qiagen, Dusseldorf, Germany). RNA was reverse transcribed using the Prime Script First-strand DNA Synthesis Kit (Takara, Osaka City, Japan). qPCR was performed on an ABI 7500 Real-time PCR System (Applied Biosystems, Carlsbad, California, USA) using TB Green Premix Ex Taq (Takara, Osaka City, Japan). β-actin was used as an internal control. The primer sequences are available in Table S3.

### Western blotting analysis of ferroptosis-related protein expression in *Eh*Prx-stimulated Caco2 cells

Treated Caco2 cells were extracted in cell lysate containing a 1% protease inhibitor cocktail (Roche, Basel, Switzerland) for western blotting. The primary antibodies included Mouse anti-beta Actin (Abcam, Cambridge, UK), Rabbit anti-Glutathione Peroxidase 4 (Abcam, Cambridge, UK), Rabbit anti-COX2 (Cell Signaling Technology, Danvers, Massachusetts, USA), Anti-xCT (Abcam, Cambridge, UK), and anti-MD2 (Abcam, Cambridge, UK). The secondary antibodies were Goat anti-Mouse IgG H&L-HRP (Abcam, Cambridge, UK) and Goat anti-Rabbit IgG H&L-HRP (Abcam, Cambridge, UK). ImageJ 1.52a (Wayne Rasband, National Institutes of Health, Bethesda, Maryland, USA) was used to evaluate signal intensity.

### Statistical analyses

All statistical data are represented as mean ± SEM. An unpaired two-tailed *t*-test or ANOVA was used to analyze the statistical significance in GraphPad Prism v.8.0 software (GraphPad Software, San Diego, California, USA). A *P* value < 0.05 was considered statistically significant (*P < 0.05; **P < 0.01) for all analyses.

## Data Availability

The authors confirm that the data supporting the findings of this study are available within the article and its supplementary materials. And the data are also available upon request by contacting the corresponding author. The raw data generated from the RNA-seq is deposited into the NCBI database with reference “PRJNA1145155.” Meanwhile, the protein crystal structure data is deposited into the NMDC database with reference “NMDCS0000029.” These data will be available upon publication.

## SUPPLEMENTAL MATERIAL

Supplemental figures and tables spectrum.00434-26-s0001.pdf Fig. S1 to S5 and Tables S1 to S5.

ASM does not own the copyrights to Supplemental Material that may be linked to, or accessed through, an article. The authors have granted ASM a non-exclusive, world-wide license to publish the Supplemental Material files. Please contact the corresponding author directly for reuse.
