# Supplementary material for: Interactions of Entamoeba histolytica peroxiredoxin with the TLR4/MD2 complex trigger ferroptosis in host cells
Source: Microbiol Spectr. 2026 Jul 20;14(9):e00434-26. doi: 10.1128/spectrum.00434-26 (PMC13532219; doi:10.1128/spectrum.00434-26)
Supplement: Supplementary file 1 [file spectrum.00434-26-s0001.pdf]

Supplementary information:

Interactions of *Entamoeba histolytica* peroxiredoxin with the TLR4/MD2 complex trigger ferroptosis in host cells

Ruixue Zhou[1#], Wenjie Li[1#], Qingtong Zhou[2], Yanqing Zhao[1], Xia Li[1, 3], Hongze Zhang[1], Dai Dong[1], Wenwen Jing[1], Hiroshi Tachibana[4], Meng Feng[1*], Gang Fu[5, 6*], Xunjia Cheng[1*]

[1] Department of Medical Microbiology and Parasitology, School of Basic Medical Sciences, Fudan University, Shanghai, 200032, China.

[2] Department of Pharmacology, School of Basic Medical Sciences, Fudan University, Shanghai, 200032, China.

[3] Affiliated Hospital of Guizhou Medical University, Guiyang, Guizhou, 550004, China.

[4] Department of Infectious Diseases, Tokai University School of Medicine, Isehara, Kanagawa 259-1193, Japan.

[5] Multiscale Research Institute of Complex Systems, Fudan University, Shanghai, 200433, China.

[6] Structural Biology Core Facility, Shenzhen Medical Academy of Research and Translation (SMART), Shenzhen, Guangdong, 518107, China.

# These authors contributed equally to this work.

* mengfeng@fudan.edu.cn (MF), fugang@smart.org.cn (GF), xjcheng@shmu.edu.cn (XC)

Supplementary figures

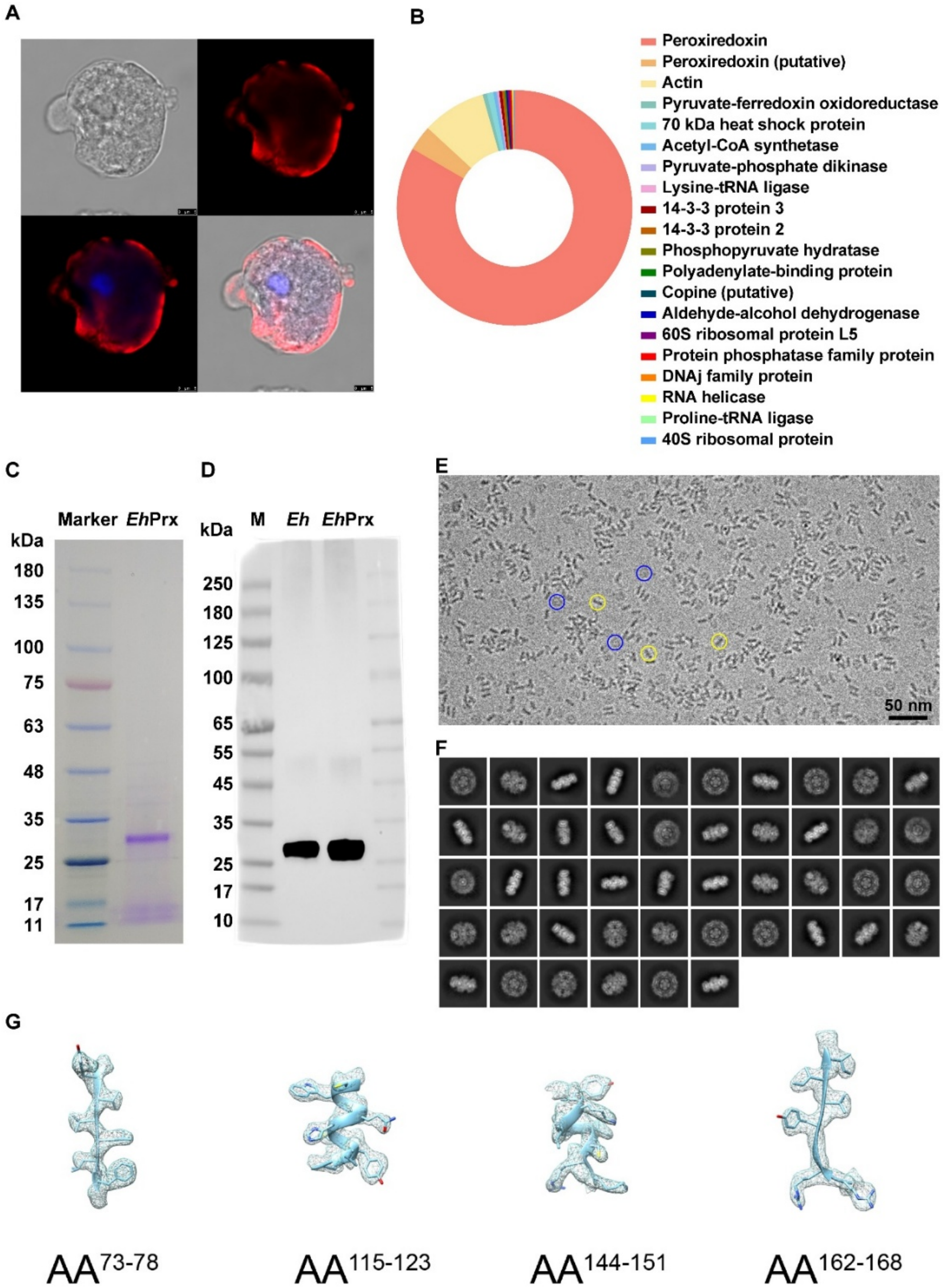

Fig S1 Purified the protein with 4G6 monoclonal antibody and screening particles of *Eh*Prx. **(A)** Confocal microscopy observations the *E. histolytica* trophozoites. The blue light was identified as the nucleus (DAPI), and the red light was identified as *Eh*Prx with mouse-4G6 monoclonal antibody and Alexa Fluor Goat anti Mouse-594. The *Eh*Prx protein was purified by immunoaffinity chromatography and subsequently dialyzed and concentrated. It was then subjected to mass spectrometry analysis (**B**)

and reducing SDS-PAGE electrophoresis and stained with Coomassie brilliant blue (**C**). (**D**) The lysate of *E. histolytica* trophozoites and the purified *Eh*Prx were identified by Western blot, with the primary antibody being the *Eh*Prx-specific 4G6 monoclonal antibody. (**E**) Check plot for *Eh*Prx using direct electron detection imaging. *Eh*Prx polymerization to form doughnut-shaped ring structures, with blue and yellow circles labelling the top-facing and side-facing ring structures, respectively. Scale length 50 nm. (**F**) 2D classifications of 46 photographs selected to represent the different morphologies of *Eh*Prx ring structure particles. (**G**) Representative regions of the *Eh*Prx cryo-EM map in mesh layout, with respective modelled residues shown as a stick representation.

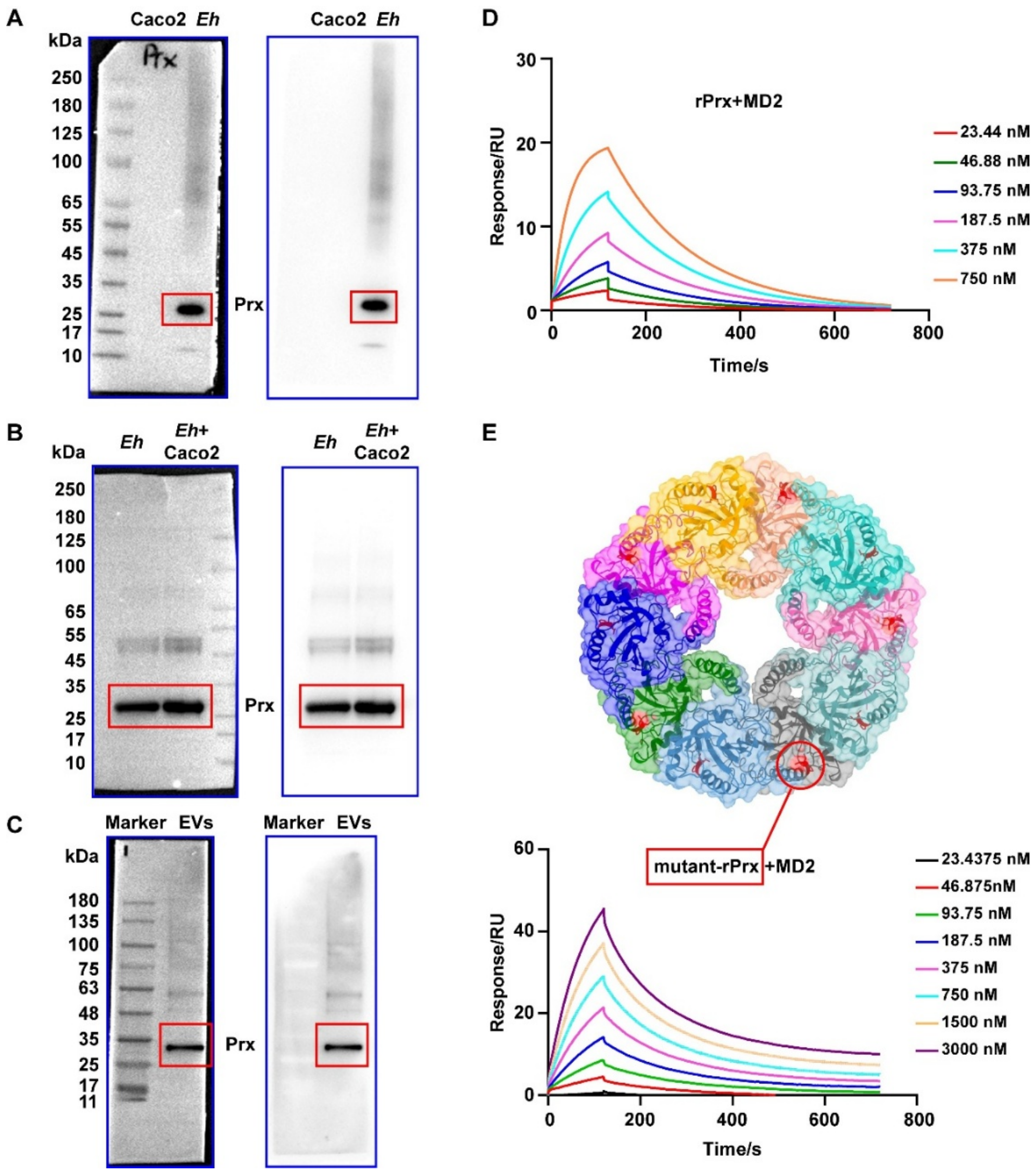

Fig S2 Expression of *Eh*Prx and interaction between *Eh*Prx and MD2. (**A**) Western blotting analysis was conducted to detect the content of *Eh*Prx in the cleavage products of Caco2 and *E. histolytica* (*Eh*); (**B**) as well as in the culture supernatants of *E. histolytica* alone (*Eh*) and in combination with Caco2 (*Eh*+Caco2) (Cold acetone method for protein concentration). (**C**) Western blotting analysis of *Eh*Prx in EVs derived from *E. histolytica* trophozoites.

Binding dissociation curves of rPrx and mutant-rPrx interacting with MD2, respectively. Both plasmids utilized for protein expression were derived from previously established laboratory stocks (Reference 20 of the main text). The SPR assay was employed to ascertain the interactions. (**D**) rPrx with MD2: K_a (1/ Ms) = 3.068×10^4, K_d(1/s) = 5.793×10^{-3}, K_D(M) = 1.888×10^{-7}; (**E**) mutant-rPrx (Enzyme active sites: VCP→VGP) with MD2: K_a(1/Ms) = 3.266×10^3, K_d(1/s) = 3.177×10^{-3}, K_D(M) = 9.729×10^{-7}.

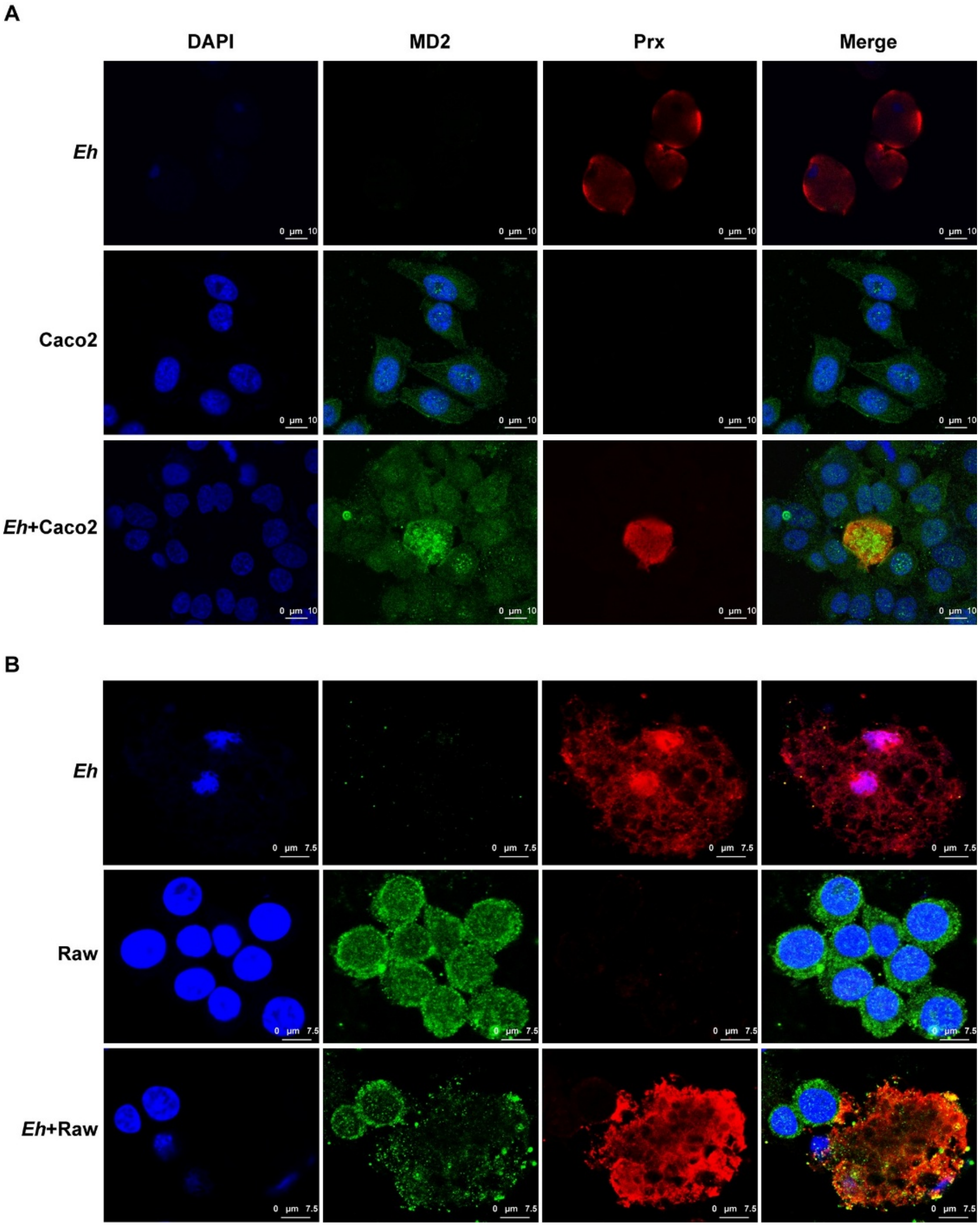

Fig S3 Confocal microscopy observations of *E. histolytica* trophozoites co-incubated with Caco2 (**A**) and Raw264.7 (**B**) cells. Blue fluorescence was used to label nuclei. Green fluorescence corresponds to MD2 observed in Caco2 and Raw264.7 cells, while red fluorescence corresponds to Prx observed in *E. histolytica* trophozoites.

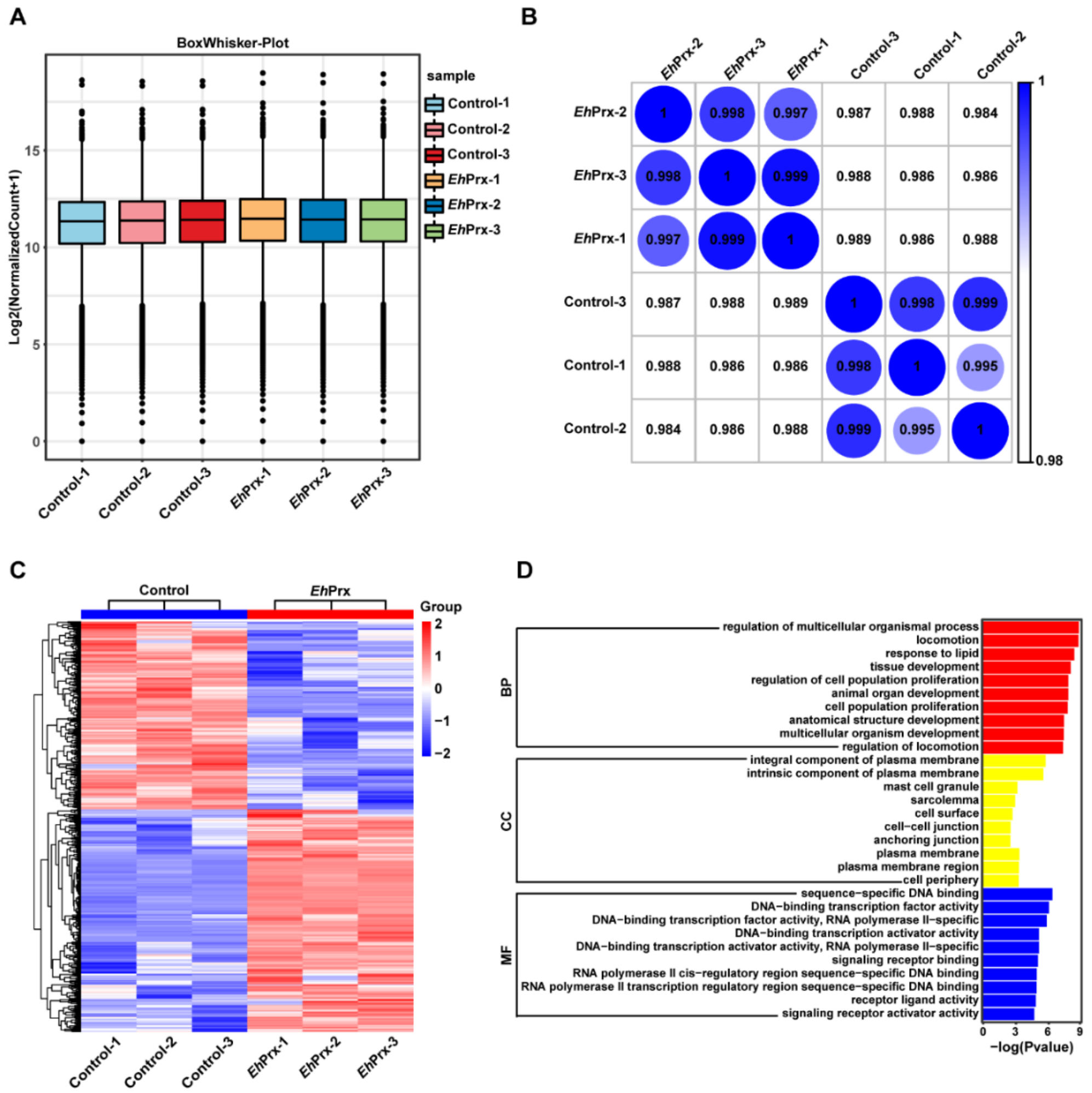

Fig S4 *Eh*Prx influenced the transcriptional profile of Caco2 cells. FPKM expression interval statistics, (**A**) Boxplot is a method of describing data using five statistics: minimum, first quartile (25%), median (50%), third quartile (75%), and maximum, which can also give a rough indication of whether there is symmetry in the gene expression of the various samples, the degree of dispersion of the distribution, and other information. (**B**) Sample correlation coefficient clusters are hierarchical clusters constructed by calculating the correlation coefficient between samples. (**C**) Analysis of differentially expressed genes between samples from *Eh*Prx and Control groups, clustered heatmap: each row represented one gene, each column represented a sample, white indicated the mean expression of significantly different genes, red indicated elevated, and blue indicated decreased. (**D**) Significantly enriched GO histogram, BP: Biological Process; CC: Cell Component; MF: Molecular Function.

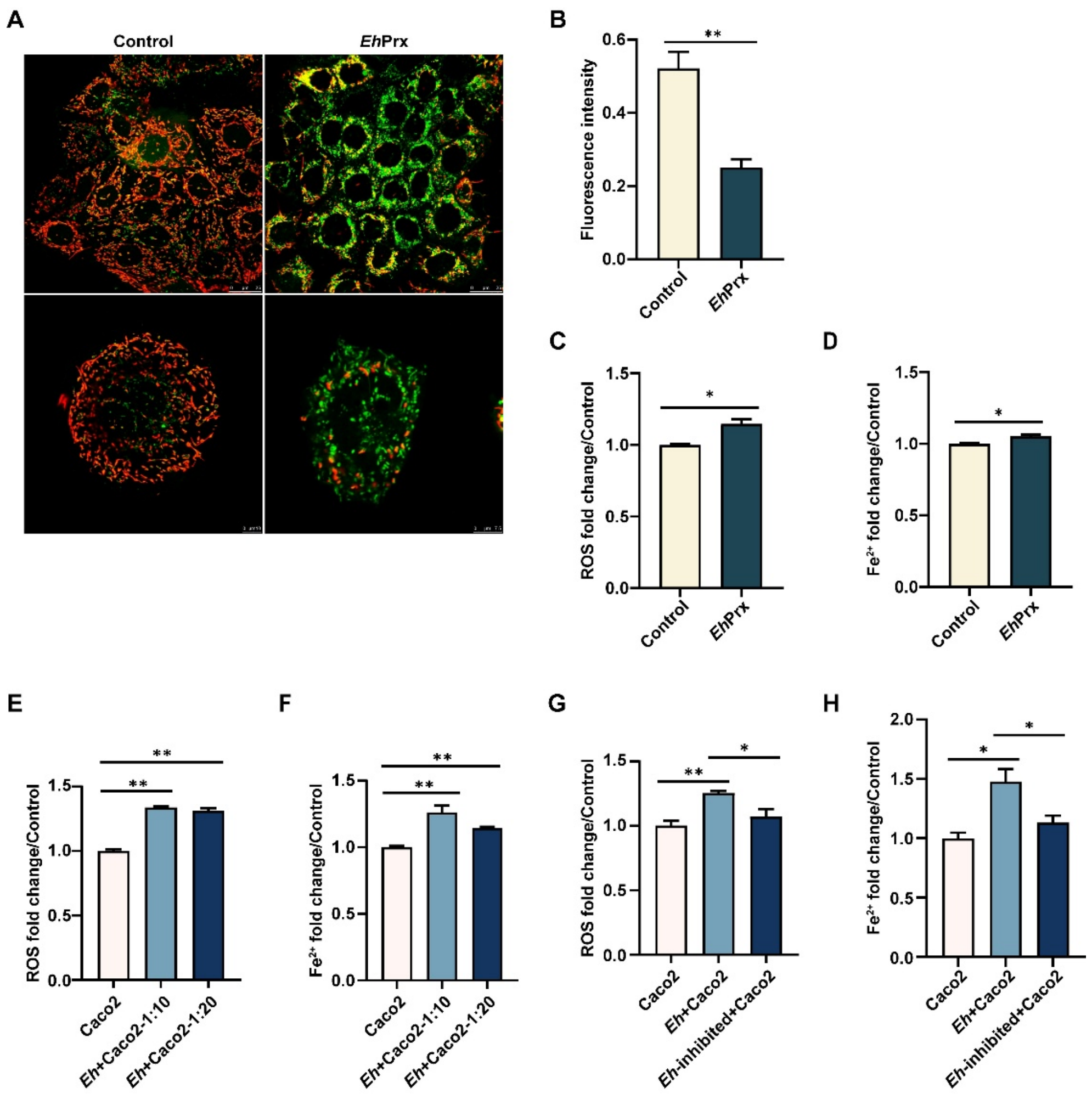

Fig S5 The biochemical indicators of ferroptosis in Caco2 cells stimulated with *Eh*Prx and *E. histolytica* trophozoites. The mitochondrial membrane potential was quantified using the Mitochondrial Membrane Potential Fluorescent Probe Kit following the stimulation of Caco2 cells with *Eh*Prx at a working concentration of 2.5 μg mL^{-1} for 6 hours, (**A**) Confocal microscopy observation; (**B**) Fluorescence enzyme marker assay results. Data are presented as mean ± SEM; n=3, ***P*<0.01, by an unpaired two tailed t-test. (**C, D**) Caco2 cells were stimulated with *Eh*Prx (2.5 μg mL^{-1}, 6 hours). Intracellular ROS (C) and Fe^{2+} (D) changes were then quantified. Data are presented as mean ± SEM; n = 3; **P* < 0.05 by unpaired two-tailed t-test. (**E, F**) Caco2 cells were stimulated with *E. histolytica* trophozoites for 2 hours at ratios of 1:10 and 1:20. Intracellular ROS (E) and Fe^{2+} (F) changes were assessed. Data are presented as mean ± SEM; n = 3; ***P* < 0.01 by ordinary one-way ANOVA. (**G, H**) Caco2 cells were co-incubated with *E. histolytica* trophozoites (untreated or pretreated with 10 μM Dinaciclib for 24 hours) at a ratio of 1:10. After 2 hours, intracellular ROS (G) and Fe^{2+} (H) changes were assessed. Data represent mean ± SEM (n = 3); **P*<0.05, ***P* < 0.01 (ordinary one-way ANOVA).

Supplementary tables

Table S1 Cryo-EM data collection, refinement and validation statistics of *Eh*Prx.

**Data collection and processing**	
TEM	Krios G3
Detector	K3
Magnification	105 000x
Voltage (kV)	300
Electron exposure (e^{-}/Å^2)	50
Defocus range (μm)	-1 ~ -2
Pixel size (Å)	0.85
Symmetry imposed	C5
Initial particle images (no.)	7 743
Final particle images (no.)	7 444
Map resolution (Å)-(FSC threshold: 0.143)	3.15
**Refinement**	
**R.m.s. deviations**	
Bond lengths (Å)	0.004
Bond angles (°)	0.933
**Validation**	
MolProbity score	2.24
Clashscore	7.94
Poor rotamers (%)	3.57
**Ramachandran plot**	
Favored (%)	94.38
Allowed (%)	5.62
Disallowed (%)	0.00

Table S2 Three possible complex structures were derived by the Protein-Protein Docking.

Names	PIPER pose energy	PIPER pose score	PIPER cluster size
TLR4-MD2-Prx-docking_pose_13_-_minimized	-739.083000	-312.800000	22
TLR4-MD2-Prx-docking_pose_3_-_minimized.pdb	-906.823000	-468.650000	82
TLR4-MD2-Prx-docking_pose_11_-_minimized.pdb	-745.598000	-410.992000	16

Table S3 qPCR primers.

Primers	Sequences (5’-3’)
β-actin-F	CTCCATCCTGGCCTCGCTGT
β-actin-R	GCTGTCACCTTCACCGTTCC
GPX4-F	ACAAGAACGGCTGCGTGGTGAA
GPX4-R	GCCACACACTTGTGGAGCTAGA
SLC7A11-F	GGGGTCCTGTCACTATTTG
SLC7A11-R	CCAGAATGTAGCGTCCAA
ACSL4-F	GCTATCTCCTCAGACACACCGA
ACSL4-R	AGGTGCTCCAACTCTGCCAGTA
PTGS2-F	CGGTGAAACTCTGGCTAGACAG
PTGS2-R	GCAAACCGTAGATGCTCAGGGA
FTH1-F	TGAAGCTGCAGAACCAACGAGG
FTH1-R	GCACACTCCATTGCATTCAGCC
SAT1-F	CTCCGGAAGGACACAGCATT
SAT1-R	CACCTCATTGCAACCTGGCT

Table S4 Data of quantitative mass spectrometry in Fig 3A

Protein	Eh-1	Eh-2	Eh-Caco2-1	Eh-Caco2-2
● 5-nitroimidazole antibiotic resistance protein	378255	422915	75901.3	92849.6
● Papain-family cysteine protease	384585	371738	1.50E+06	1.49E+06
● Aldose reductase	6.70E+06	6.97E+06	4.97E+06	4.35E+06
● Cysteine protease 19	430626	352820	73551.3	72006.5
● Cysteine protease binding protein family 1	795569	751033	1.82E+06	2.01E+06
● Cysteine proteinase 1	5.75E+06	5.04E+06	3.96E+06	4.27E+06
● Cysteine proteinase 2	6.50E+06	7.37E+06	3.89E+06	3.01E+06
● Cysteine synthase 3	2.66E+06	3.00E+06	993347	776210
● NADPH-dependent fmn reductase	2261920	2414630	6573660	7459970
● Phosphopantothenoylcystein e decarboxylase	236746	219016	566583	475830
● Purine nucleoside phosphorylase	7.66E+06	8.84E+06	7.11E+06	7.25E+06
● Nitroreductase TM1586	104040	61533.4	122606	150984
● Serine/threonine-protein phosphatase	226557	233011	161807	208272
● Splicing factor arginine serine-rich	138796	90631.7	322715	299538
● Thioredoxin	1.02E+07	9.69E+06	2.22E+06	2.18E+06
● Thioredoxin reductase	5.39E+06	5.70E+06	4.13E+06	4.28E+06
● Peroxiredoxin	1.40E+07	1.28E+07	2.55E+07	3.50E+07

● Tubulin-tyrosine ligase	254580	290258	337653	447720
● Tyrosine-tRNA ligase	636162	747899	494612	472357
● Villidin	572817	518390	1833130	1492540
● Glucose-6-phosphate isomerase	3587600	4028240	6622730	5827560
● Protein kinase	122222	281203	375337	350936
● Autophagy protein 5	55413	51487	63250.6	62106
● Sulfotransferase	814954	805166	360914	287910
● Phosphoglycerate kinase	11324300	11488700	8394440	9694490
● D-glycerate dehydrogenase	1436490	1471640	2313020	2369910
● Phosphoserine transaminase	7172150	6761000	20528700	18944500
● EhRab7E	699152	655066	1263730	1177320
● EhRab7D	238408	214304	188911	185464
● EhRabx36	403684	378722	283050	299034
● Ran GTPase	1923950	1929360	54274800	58910700
● PAX6	734642	696864	417003	408288
● Lactotransferrin	208826000	186194000	5862130	6657600
● Vitamin D-binding protein	131207000	121755000	21956500	22223600
● Fibronectin	916696	989849	7458870	7651630
● IGF2	177429	144576	486565	424141
● Ceruloplasmin	14816400	14167600	5997150	6413850
● UMP/CMP kinase	441110	481670	2455310	2729360

Table S5 Data of EhPrx-mass spectrometry in FigS1B-

Protein FDR Confidence	Accession	Description	Exp. q-value	Sum PEP Score	Coverage [%]	Peptides	PSMs	Unique Peptides	AAs	MW [kDa]	calc. pI	Score Sequest HT	Peptides	Razor Peptides	Abundance: F1: Sample
High	A0A5K1TV90	thioredoxin-dependent peroxiredoxin OS=Entamoeba histolytica OX=5759 GN=CL6EHI_123390 PE=3 SV=1	0	125.816	49	31	1364	4	237	26.7	7.88	1700.86	31	33	6727223324
High	A0A5K1V0Y5	thioredoxin-dependent peroxiredoxin OS=Entamoeba histolytica OX=5759 GN=CL6EHI_122310 PE=3 SV=1	0	123.574	48	30	1384	3	237	26.8	7.9	1699.65	30	0	280778066.1
High	A0A5K1UYW3	Actin OS=Entamoeba histolytica OX=5759 GN=CL6EHI_107290 PE=2 SV=1	0	119.015	48	27	256	27	376	42	5.33	316.08	27	0	708078921.7
High	A0A5K1VRG4	Pyruvate ferredoxin oxidoreductase OS=Entamoeba histolytica OX=5759 GN=CL6EHI_051060 PE=4 SV=1	0	89.98	24	32	125	32	1162	127.6	6.79	115.16	32	0	47502201.05
High	A0A5K1V184	70 kDa heat shock protein putative OS=Entamoeba histolytica OX=5759 GN=CL6EHI_199590 PE=3 SV=1	0	71.828	28	26	81	26	661	73.4	5.15	126.72	26	0	71889814.99
High	A0A5K1UIH2	Acetyl-coa synthetase putative OS=Entamoeba histolytica OX=5759 GN=CL6EHI_178960 PE=4 SV=1	0	68.737	19	19	73	19	713	77.4	6.27	104.26	19	0	34284347.2
High	A0A5K1UCC2	Pyruvate, phosphate dikinase OS=Entamoeba histolytica	0	59.347	24	20	39	20	885	97.8	6.18	46.64	20	0	15723279.94

		OX=5759 GN=CL6EHI_009530 PE=3 SV=1													
High	A0A5K1VR98	Lysine--tRNA ligase OS=Entamoeba histolytica OX=5759 GN=CL6EHI_047810 PE=3 SV=1	0	52.184	15	15	54	15	769	86.5	6.44	72.09	15	0	14132067.34
High	A0A5K1VB56	14-3-3 protein 3 OS=Entamoeba histolytica OX=5759 GN=CL6EHI_006810 PE=3 SV=1	0	47.408	49	15	38	13	240	27.5	4.84	54.41	15	1	31854560.63
High	A0A5K1UQU0	14-3-3 protein 2 OS=Entamoeba histolytica OX=5759 GN=CL6EHI_098280 PE=3 SV=1	0	40.709	47	13	35	10	238	27.5	4.88	49.47	13	2	25476484.61
High	A0A5K1TUF6	phosphopyruvate hydratase OS=Entamoeba histolytica OX=5759 GN=CL6EHI_130700 PE=3 SV=1	0	40.129	29	10	25	10	436	47.3	6.21	47.04	10	0	9795087.805
High	A0A5K1U4I6	Polyadenylate-binding protein putative OS=Entamoeba histolytica OX=5759 GN=CL6EHI_033250 PE=4 SV=1	0	35.58	26	13	26	13	514	58.8	8.51	39.92	13	0	11179404.05
High	A0A5K1UDP6	Copine putative OS=Entamoeba histolytica OX=5759 GN=CL6EHI_137110 PE=4 SV=1	0	32.771	16	5	8	5	273	30.5	5.06	16.58	5	0	2616905.906
High	A0A5K1TW74	Aldehyde-alcohol dehydrogenase OS=Entamoeba histolytica OX=5759 GN=CL6EHI_160940 PE=3 SV=1	0	30.186	16	16	29	16	870	95.5	7.25	18.65	16	0	12169008.45
High	A0A5K1UJ75	60S ribosomal protein L5 putative OS=Entamoeba histolytica OX=5759 GN=CL6EHI_006860 PE=2 SV=1	0	29.737	31	9	26	9	286	32.4	9.8	49.9	9	0	26213117.66

High	A0A5K1TXD2	Protein phosphatase family protein OS=Entamoeba histolytica OX=5759 GN=CL6EHI_179330 PE=3 SV=1	0	28.462	32	9	29	9	282	31.6	6.93	35.66	9	0	17899877.98
High	A0A5K1TWC6	DNAj family protein OS=Entamoeba histolytica OX=5759 GN=CL6EHI_151260 PE=3 SV=1	0	26.604	12	5	10	5	400	45.1	7.18	20.85	5	0	7806977.688
High	A0A5K1U177	RNA helicase OS=Entamoeba histolytica OX=5759 GN=CL6EHI_093900 PE=3 SV=1	0	26.471	24	9	26	9	391	44	5.43	14.35	9	0	10346101.66
High	A0A5K1VGH7	proline--tRNA ligase OS=Entamoeba histolytica OX=5759 GN=CL6EHI_040400 PE=3 SV=1	0	26.008	24	9	18	9	536	61.1	6.99	12.19	9	0	2247124.328
High	A0A5K1TWH2	Small ribosomal subunit protein uS2 OS=Entamoeba histolytica OX=5759 GN=CL6EHI_081410 PE=2 SV=1	0	24.207	21	6	21	6	254	28.6	8.05	42.58	6	0	12156096.5
High	A0A5K1VRP8	Mir domain protein OS=Entamoeba histolytica OX=5759 GN=CL6EHI_100480 PE=4 SV=1	0	24.075	27	6	27	6	211	23.4	6.71	44.86	6	0	80072357.92
High	A0A5K1TYX7	Elongation factor 1-alpha OS=Entamoeba histolytica OX=5759 GN=CL6EHI_011210 PE=3 SV=1	0	24.024	23	10	27	10	442	48.4	8.97	33.16	10	0	20928156.95
High	Q9BLE4	small GTPase Rab11B OS=Entamoeba histolytica OX=5759 GN=EhRab11B PE=2 SV=1	0	23.7	37	9	35	6	211	23.8	5.08	46.46	9	3	34459020.69

High	Q24806	Ras family GTPase OS=Entamoeba histolytica OX=5759 GN=Ehras1 PE=3 SV=1	0	23.651	27	7	19	7	205	22.7	5.5	21.07	7	0	12337191.88
High	A0A175JM79	S-adenosylmethionine synthase OS=Entamoeba histolytica OX=5759 GN=CL6EHI_004920 PE=3 SV=1	0	22.763	20	6	15	6	397	43.6	6.42	33.55	6	0	4603762.75
High	A0A5K1V9T7	Type A flavoprotein, putative OS=Entamoeba histolytica OX=5759 GN=CL6EHI_096710 PE=2 SV=1	0	22.3	14	6	17	6	406	46	6.29	20.94	6	0	12474014.44
High	A0A5K1UHS8	Nitrogen fixation protein NifU OS=Entamoeba histolytica OX=5759 GN=CL6EHI_049620 PE=3 SV=1	0	22.232	31	10	18	10	348	38.9	6.02	19.35	10	0	3693156.719
High	A0A5K1V4U9	14-3-3 protein 1 OS=Entamoeba histolytica OX=5759 GN=CL6EHI_025360 PE=3 SV=1	0	22.112	31	9	28	5	239	27.3	4.89	17.12	9	0	3583335.438
High	A0A5K1VKQ3	Nucleolar protein 56 OS=Entamoeba histolytica OX=5759 GN=CL6EHI_122740 PE=3 SV=1	0	22.062	18	7	10	7	453	51.5	6.7	17.31	7	0	2387959.516
High	A0A5K1VA97	Glyceraldehyde-3-phosphate dehydrogenase OS=Entamoeba histolytica OX=5759 GN=CL6EHI_008200 PE=2 SV=1	0	21.83	19	9	22	9	334	36	7.43	24.17	9	0	20863413.3
High	A0A5K1VKX2	Heat shock protein 70 family OS=Entamoeba histolytica OX=5759 GN=CL6EHI_001950 PE=3 SV=1	0	21.776	15	10	20	10	660	73.9	5.21	23.78	10	0	12388070.27

High	A0A5K1U1W4	19S CAP proteasome s2 subunit OS=Entamoeba histolytica OX=5759 GN=CL6EHI_198010 PE=4 SV=1	0	21.716	13	8	13	8	843	92	5.39	26.84	8	0	6253792.906
High	A0A5K1V686	Methionine aminopeptidase 2 OS=Entamoeba histolytica OX=5759 GN=CL6EHI_126880 PE=3 SV=1	0	21.224	15	7	18	7	413	46	6.61	23.36	7	0	9001663.594
High	A0A5K1UDU7	40S ribosomal protein S9 OS=Entamoeba histolytica OX=5759 GN=CL6EHI_125780 PE=2 SV=1	0	20.736	32	10	28	10	185	21.5	10.35	31.83	10	0	33068007.06
High	A0A5K1UR51	Uncharacterized protein OS=Entamoeba histolytica OX=5759 GN=CL6EHI_164470 PE=4 SV=1	0	20.661	5	4	5	4	807	93.7	6.25	10.03	4	0	961039.1563
High	S0AVN2	MIB/HERC2 domain-containing protein OS=Entamoeba histolytica OX=5759 GN=CL6EHI_188760 PE=2 SV=1	0	20.628	27	7	65	7	245	27.7	5.5	60.75	7	0	6081418.875
High	A0A5K1UQ71	Actin-like protein putative OS=Entamoeba histolytica OX=5759 GN=CL6EHI_111050 PE=3 SV=1	0	19.901	18	6	17	6	401	44.6	6.57	27.35	6	0	8988039.906
High	S0B1L4	DNAJ homolog subfamily A member 1, putative OS=Entamoeba histolytica OX=5759 GN=CL6EHI_182520 PE=2 SV=1	0	19.533	17	7	19	7	346	39.3	8.09	30.4	7	0	7467264.234
High	A0A5K1U9T2	Gal galnac lectin light subunit OS=Entamoeba histolytica	0	19.504	23	5	12	3	270	31.6	8.25	11.06	5	0	

		OX=5759 GN=CL6EHI_148790 PE=4 SV=1													
High	A0A5K1UUB0	Galactose-inhibitable lectin 35 kDa subunit OS=Entamoeba histolytica OX=5759 GN=CL6EHI_035690 PE=4 SV=1	0	19.34	19	5	15	2	288	33.6	9.17	12.28	5	5	4125356.828
High	A0A5K1VMA9	Proteasome regulatory subunit putative OS=Entamoeba histolytica OX=5759 GN=CL6EHI_054230 PE=4 SV=1	0	18.336	21	6	9	6	276	32	5.43	15.01	6	0	5221149.313
High	A0A5K1UNT2	Dynamin-like protein OS=Entamoeba histolytica OX=5759 GN=CL6EHI_052740 PE=3 SV=1	0	18.238	10	6	12	6	663	74.6	6.84	8.78	6	0	1497872.813
High	A0A5K1UHS4	Nucleotide binding protein 2 putative OS=Entamoeba histolytica OX=5759 GN=CL6EHI_000610 PE=3 SV=1	0	17.825	13	3	8	3	273	29.3	5.15	17.89	3	0	7723318.43
High	A0A5K1U4L0	Purine nucleoside phosphorylase putative OS=Entamoeba histolytica OX=5759 GN=CL6EHI_200080 PE=4 SV=1	0	17.58	19	8	18	8	318	34.7	5.92	14.33	8	0	4142159
High	A0A175JFL3	START domain-containing protein OS=Entamoeba histolytica OX=5759 GN=CL6EHI_178560 PE=4 SV=1	0	17.473	33	7	11	7	226	26.5	8.51	8.73	7	0	1794647.203
High	A0A5K1UPW0	Tubulin alpha chain OS=Entamoeba histolytica OX=5759 GN=CL6EHI_005950 PE=3 SV=1	0	17.245	10	4	8	4	455	50.2	5.92	15.43	4	0	827945.8594

High	A0A5K1VN14	60S ribosomal protein L9 putative OS=Entamoeba histolytica OX=5759 GN=CL6EHI_193080 PE=3 SV=1	0	17.108	28	5	7	5	197	22.3	9.36	9.47	5	0	466763.5313
High	S0AY27	Aminoacyl-histidine dipeptidase putative OS=Entamoeba histolytica OX=5759 GN=CL6EHI_042170 PE=2 SV=1	0	16.879	13	6	9	6	516	56.9	6.1	13.51	6	0	698770.625
High	A0A5K1U7N6	Tubulin beta chain OS=Entamoeba histolytica OX=5759 GN=CL6EHI_049920 PE=3 SV=1	0	16.658	6	3	10	3	459	51.1	5.05	17.48	3	0	1444046.188
High	A0A5K1V4M5	phosphoglycerate mutase (2,3-diphosphoglycerate-independent) OS=Entamoeba histolytica OX=5759 GN=CL6EHI_050940 PE=3 SV=1	0	16.613	11	5	10	5	555	61.8	6.24	12.21	5	0	2830976.063
High	A0A5K1VB02	Purine nucleoside phosphorylase putative OS=Entamoeba histolytica OX=5759 GN=CL6EHI_048740 PE=4 SV=1	0	16.486	21	6	10	6	320	35.1	7.02	13.58	6	0	1961660.906
High	A0A5K1USW9	Alcohol dehydrogenase 3 putative OS=Entamoeba histolytica OX=5759 GN=CL6EHI_198760 PE=4 SV=1	0	15.988	14	6	13	6	384	42.5	6.98	5.09	6	0	775564.0156
High	A0A5K1UJN7	WD repeat protein OS=Entamoeba histolytica OX=5759 GN=CL6EHI_038880 PE=2 SV=1	0	15.708	21	5	6	4	322	35.7	5.97	13.87	5	1	4516112.594

High	A0A060N036	galactokinase OS=Entamoeba histolytica OX=5759 GN=CL6EHI_094100 PE=2 SV=1	0	15.53	12	5	16	5	389	43.4	6.54	26.2	5	0	4382121.961
High	A0A5K1VME4	Xaa-pro dipeptidase putative OS=Entamoeba histolytica OX=5759 GN=CL6EHI_010070 PE=3 SV=1	0	15.516	14	6	12	6	471	53.9	6.1	11.5	6	0	2149528.063
High	A0A5K1VAT6	threonine--tRNA ligase OS=Entamoeba histolytica OX=5759 GN=CL6EHI_103730 PE=3 SV=1	0	15.475	11	8	18	8	676	77.8	7.01	5.91	8	0	3701890.75
High	A0A5K1UG59	Non-transporter abc protein putative OS=Entamoeba histolytica OX=5759 GN=CL6EHI_175440 PE=4 SV=1	0	15.458	7	6	8	6	724	83.3	6.09	11.08	6	0	2082856.719
High	S0AY55	Galactose-specific adhesin light subunit putative OS=Entamoeba histolytica OX=5759 GN=CL6EHI_049690 PE=2 SV=1	0	15.051	15	4	12	1	288	33.9	8.91	4.02	4	0	
High	A0A5K1VLZ8	Synapsin putative OS=Entamoeba histolytica OX=5759 GN=CL6EHI_127220 PE=3 SV=1	0	14.965	14	4	8	4	314	36.2	7.68	13.99	4	0	3969714.672
High	A0A175JX50	von willebrand factor type a domain containing protein OS=Entamoeba histolytica OX=5759 GN=CL6EHI_187800 PE=4 SV=1	0	14.68	9	5	8	5	577	65.4	5.15	9.67	5	0	1601243.469
High	A0A5K1U9J0	Sulfate adenylyltransferase putative OS=Entamoeba histolytica OX=5759 GN=CL6EHI_179080 PE=3 SV=1	0	14.25	12	5	8	5	477	52.7	9.01	11.48	5	0	7939469.586

High	A0A5K1UCJ8	phosphoglucomutase (alpha-D-glucose-1,6-bisphosphate-dependent) OS=Entamoeba histolytica OX=5759 GN=CL6EHI_110120 PE=3 SV=1	0	14.234	10	5	12	5	553	60.8	6.29	18	5	0	2669218.813
High	A0A5K1V8N2	Arginase OS=Entamoeba histolytica OX=5759 GN=CL6EHI_152330 PE=3 SV=1	0	14.145	12	3	7	3	296	32.8	5.83	16.64	3	0	1202762.156
High	A0A060N1J3	Galactose-inhibitable lectin putative OS=Entamoeba histolytica OX=5759 GN=CL6EHI_058330 PE=2 SV=1	0	14.075	22	5	13	2	271	31.6	8.43	7.52	5	0	390943.4688
High	A0A5K1UJZ1	Cysteine desulfurase putative OS=Entamoeba histolytica OX=5759 GN=CL6EHI_136380 PE=3 SV=1	0	13.449	15	5	8	5	390	42.8	6.34	13.6	5	0	3843732.063
High	A0A5K1V8N8	AP-3 complex subunit mu OS=Entamoeba histolytica OX=5759 GN=CL6EHI_099240 PE=3 SV=1	0	13.308	17	6	7	6	426	48	8.31	6.88	6	0	1758256.953
High	A0A5K1VR65	Ngg1-interacting factor 3 putative OS=Entamoeba histolytica OX=5759 GN=CL6EHI_161860 PE=3 SV=1	0	13.154	18	5	22	5	267	30	5.64	24.75	5	0	16138571.06
High	A0A5K1URY9	Ricin B lectin domain-containing protein OS=Entamoeba histolytica OX=5759 GN=CL6EHI_138510 PE=4 SV=1	0	13.044	10	5	57	5	279	32.3	5.26	122.41	5	0	55287097.14
High	A0A175K110	fumarate hydratase OS=Entamoeba histolytica OX=5759 GN=CL6EHI_201720 PE=3 SV=1	0	12.99	10	4	8	4	565	62.2	6.89	5.1	4	0	769672.3047

High	A0A5K1UTC4	MIB/HERC2 domain-containing protein OS=Entamoeba histolytica OX=5759 GN=CL6EHI_181140 PE=2 SV=1	0	12.965	21	6	11	6	219	25.1	5.73	11.32	6	0	1277025.594
High	A0A5K1UA40	RuvB-like helicase OS=Entamoeba histolytica OX=5759 GN=CL6EHI_091070 PE=3 SV=1	0	12.956	15	7	9	7	449	49.3	5.95	16.52	7	0	1359481.594
High	A0A5K1VRQ8	Heat shock protein 70 putative OS=Entamoeba histolytica OX=5759 GN=CL6EHI_052860 PE=3 SV=1	0	12.879	13	6	6	6	656	71.5	5.47	9.6	6	0	2596711.875
High	A0A5K1VPX2	Uncharacterized protein OS=Entamoeba histolytica OX=5759 GN=CL6EHI_068510 PE=4 SV=1	0	12.694	8	5	8	5	744	82.5	5.27	7.67	5	0	1517294.656
High	A0A5K1UVQ2	Myosin heavy chain OS=Entamoeba histolytica OX=5759 GN=CL6EHI_110180 PE=3 SV=1	0	12.608	3	4	4	4	2151	246.4	5.31	6.89	4	0	1319300.859
High	A0A5K1VF67	Nucleosome assembly protein putative OS=Entamoeba histolytica OX=5759 GN=CL6EHI_139460 PE=3 SV=1	0	12.508	9	3	7	3	348	40.2	4.51	10.75	3	0	3621863.289
High	A0A5K1UY83	Protein kinase domain containing protein OS=Entamoeba histolytica OX=5759 GN=CL6EHI_121880 PE=3 SV=1	0	12.316	8	3	5	3	382	43.5	7.99	6.97	3	0	1580604.867
High	A0A5K1URM2	Enhancer binding protein OS=Entamoeba histolytica	0	12.303	22	6	10	6	306	35.3	9.73	7.24	6	0	3197935.023

		OX=5759 GN=CL6EHI_121780 PE=4 SV=1													
High	A0A5K1V9R6	Dipeptidyl peptidase 3 OS=Entamoeba histolytica OX=5759 GN=CL6EHI_110750 PE=3 SV=1	0	12.111	9	4	4	4	645	73.9	5.38	5.59	4	0	192676.6875
High	A0A5K1TXS4	SPRY domain-containing protein 7 OS=Entamoeba histolytica OX=5759 GN=CL6EHI_138060 PE=4 SV=1	0	12.009	13	3	10	3	266	30.5	8.97	15.31	3	0	4048295
High	A0A5K1UE61	60S ribosomal protein L12 putative OS=Entamoeba histolytica OX=5759 GN=CL6EHI_030710 PE=2 SV=1	0	11.856	26	4	5	4	167	17.8	9.6	7.27	4	0	7816130.758
High	A0A5K1VNY3	Uncharacterized protein OS=Entamoeba histolytica OX=5759 GN=CL6EHI_188820 PE=4 SV=1	0	11.767	6	3	3	3	540	58.7	8.84	3.4	3	0	624244.5156
High	A0A5K1V3N3	V-type proton ATPase subunit C OS=Entamoeba histolytica OX=5759 GN=CL6EHI_092600 PE=3 SV=1	0	11.707	14	6	9	6	378	44.1	6.11	7.94	6	0	3008377.25
High	A0A5K1U3T6	Ribosomal protein S4, putative OS=Entamoeba histolytica OX=5759 GN=CL6EHI_003940 PE=2 SV=1	0	11.698	20	5	32	5	326	36.7	10.36	2.38	5	0	1991650.143
High	A0A175K294	Clathrin heavy chain putative OS=Entamoeba histolytica OX=5759 GN=CL6EHI_201510 PE=4 SV=1	0	11.589	4	4	4	3	1144	129.6	5.5	4.24	4	1	515638.0391
High	S0AXQ0	G protein alpha subunit putative OS=Entamoeba	0	11.416	11	3	4	3	358	39.9	5.33	4.47	3	0	504112.8125

		histolytica OX=5759 GN=CL6EHI_140350 PE=2 SV=1												
High	A0A5K1VQA4	Proteasome regulatory subunit putative OS=Entamoeba histolytica OX=5759 GN=CL6EHI_182600 PE=3 SV=1	0	11.385	9	4	5	4	385	43.3	5.92	7.01	4	0	2339648.219
High	A0A5K1UNE8	Thioredoxin putative OS=Entamoeba histolytica OX=5759 GN=CL6EHI_021560 PE=4 SV=1	0	11.073	14	5	6	5	379	42.9	6.01	13.18	5	0	2426785
High	A0A175JF05	Rab family GTPase OS=Entamoeba histolytica OX=5759 GN=CL6EHI_067850 PE=3 SV=1	0	10.993	20	4	9	1	212	23.7	6.23	12.2	4	0	
High	A0A5K1VQE2	Histone-glutamine methyltransferase OS=Entamoeba histolytica OX=5759 GN=CL6EHI_118840 PE=3 SV=1	0	10.934	14	4	8	4	305	32.5	9.92	9.93	4	0	2160197.125
High	A0A5K1VBU4	60S ribosomal protein L18a OS=Entamoeba histolytica OX=5759 GN=CL6EHI_035600 PE=3 SV=1	0	10.816	26	5	8	5	173	20.1	10.15	8.3	5	0	2239772.734
High	S0AXD9	Fructose 1 6-bisphosphate aldolase putative OS=Entamoeba histolytica OX=5759 GN=CL6EHI_098570 PE=2 SV=1	0	10.741	18	5	11	5	330	36.2	6.54	12.08	5	0	964722.0078
High	A0A5K1UC66	inositol-3-phosphate synthase OS=Entamoeba histolytica OX=5759 GN=CL6EHI_070720 PE=3 SV=1	0	10.61	9	4	4	4	508	56.5	6.48	5.16	4	0	118788.9219

High	A0A5K 1VLR1	26s proteasome non-ATPase regulatory subunit putative OS=Entamoeba histolytica OX=5759 GN=CL6EHI_030170 PE=3 SV=1	0	10.423	15	3	6	3	314	35.8	6.33	6.72	3	0	1052019.75
High	A0A5K 1UFB1	ribose-phosphate diphosphokinase OS=Entamoeba histolytica OX=5759 GN=CL6EHI_035650 PE=3 SV=1	0	10.38	16	5	8	5	332	36.6	6.19	9.63	5	0	1550159.922
High	A0A5K 1VL93	Phospholipase B-like OS=Entamoeba histolytica OX=5759 GN=CL6EHI_114320 PE=3 SV=1	0	10.14	5	3	5	3	550	63.3	6	7.42	3	0	907378.1563
High	A0A5K 1VAG6	SnoRNA binding protein putative OS=Entamoeba histolytica OX=5759 GN=CL6EHI_183900 PE=3 SV=1	0	10.032	8	3	3	3	503	56.5	6.05	3.13	3	0	794472.4375
High	Q9503 0	Cysteine proteinase OS=Entamoeba histolytica OX=5759 GN=CP5 PE=2 SV=2	0	9.929	14	5	14	5	318	34.7	8.46	15.32	5	0	6318723.813
High	P1371 7	SWISS-PROT:P13717 Nuclease - Serratia marcescens.	0	9.903	14	4	4	4	266	28.9	7.42	6.47	4	0	103506.875
High	A0A5K 1U832	Isopentenyl phosphate kinase OS=Entamoeba histolytica OX=5759 GN=CL6EHI_178490 PE=3 SV=1	0	9.852	16	4	9	4	259	28.5	6.79	7.04	4	0	4171492.578
High	A0A5K 1V480	Proteasome regulatory subunit putative OS=Entamoeba histolytica OX=5759 GN=CL6EHI_103850 PE=4 SV=1	0	9.847	11	3	6	3	377	43.8	5.08	7.66	3	0	1480918.469

High	A0A5K 1VRV0	Adenylyl cyclase-associated protein OS=Entamoeba histolytica OX=5759 GN=CL6EHI_136150 PE=3 SV=1	0	9.793	13	5	9	5	478	53.2	6.7	5.03	5	0	10949942.5
High	A0A5K 1VFF2	serine--tRNA ligase OS=Entamoeba histolytica OX=5759 GN=CL6EHI_092640 PE=4 SV=1	0	9.715	15	7	10	7	453	52	7.28	7.78	7	0	1449738.688
High	A0A5K 1UC07	26S protease regulatory subunit putative OS=Entamoeba histolytica OX=5759 GN=CL6EHI_080890 PE=3 SV=1	0	9.552	12	4	6	3	417	46.9	5.82	5.33	4	1	1294721
High	A0A5K 1VSX8	Protein TRANSPARENT TESTA GLABRA OS=Entamoeba histolytica OX=5759 GN=CL6EHI_105120 PE=4 SV=1	0	9.551	14	3	5	3	326	36.6	5.6	4.43	3	0	771133.3516
High	A0A5K 1VRW 9	3-oxoacyl-acyl-carrier protein reductase putative OS=Entamoeba histolytica OX=5759 GN=CL6EHI_186920 PE=3 SV=1	0	9.548	11	4	8	4	265	29.7	5.17	6.24	4	0	995822.375
High	A0A5K 1UBQ0	non-specific serine/threonine protein kinase OS=Entamoeba histolytica OX=5759 GN=CL6EHI_095940 PE=3 SV=1	0	9.402	14	7	11	7	395	45.4	8.51	4.04	7	0	1854070.93
High	A0A17 5JXP1	Uncharacterized protein OS=Entamoeba histolytica OX=5759 GN=CL6EHI_113590 PE=3 SV=1	0	9.391	6	3	4	3	498	57.6	8.87	3.57	3	0	194852.7031
High	A0A5K 1USA5	Heat repeat domain containing protein OS=Entamoeba	0	9.378	7	4	4	4	707	78.3	8.44	4.34	4	0	1084025.352

		histolytica OX=5759 GN=CL6EHI_069480 PE=4 SV=1												
High	A0A5K1U9X7	Estradiol 17-beta-dehydrogenase putative OS=Entamoeba histolytica OX=5759 GN=CL6EHI_163540 PE=3 SV=1	0	9.34	14	4	8	4	319	36.5	8.57	16.96	4	0	2724994.75
High	S0AYM4	small monomeric GTPase OS=Entamoeba histolytica OX=5759 GN=CL6EHI_058090 PE=2 SV=1	0	9.236	34	5	7	5	184	20.5	6.21	4.9	5	0	1616548.875
High	S0B1M6	Cysteine proteinase 2 OS=Entamoeba histolytica OX=5759 GN=CL6EHI_033710 PE=2 SV=1	0	9.201	9	3	7	3	315	34.7	7.21	11.5	3	0	18274781.09
High	A0A5K1UN99	60S ribosomal protein L7a OS=Entamoeba histolytica OX=5759 GN=CL6EHI_103310 PE=3 SV=1	0	8.914	16	4	7	4	286	32.2	10.02	12	4	0	587628.625
High	A0A5K1U4G2	VASt domain-containing protein OS=Entamoeba histolytica OX=5759 GN=CL6EHI_024350 PE=4 SV=1	0	8.892	11	5	5	5	604	68.9	7.53	6.03	5	0	430625.4688
High	A0A5K1V8I0	dihydropyrimidine dehydrogenase (NADP(+)) OS=Entamoeba histolytica OX=5759 GN=CL6EHI_012980 PE=3 SV=1	0	8.694	4	3	6	3	901	98.7	6.57	9.68	3	0	876441.0625
High	A0A5K1VRV8	Ubiquitin carboxyl-terminal hydrolase OS=Entamoeba histolytica OX=5759 GN=CL6EHI_012290 PE=3 SV=1	0	8.679	6	4	6	4	880	102.9	5.29	4.42	4	0	1977572.438

High	A0A5K 1U6Q9	Ubiquitin putative OS=Entamoeba histolytica OX=5759 GN=CL6EHI_166800 PE=4 SV=1	0	8.634	36	3	5	3	97	11.1	9.63	7.5	3	0	222968.6094
High	A0A06 0N111	Cysteine proteinase putative OS=Entamoeba histolytica OX=5759 GN=CL6EHI_050570 PE=2 SV=1	0	8.524	13	2	3	2	311	33.7	7.34	0	2	0	124135.2266
High	A0A5K 1UGQ 8	Calcium-regulated actin-bundling protein C-terminal domain-containing protein OS=Entamoeba histolytica OX=5759 GN=CL6EHI_189930 PE=4 SV=1	0	8.518	18	3	5	3	222	25.2	8.82	5.37	3	0	1190378.531
High	Q1EQ3 8	Beta2-COP (Fragment) OS=Entamoeba histolytica OX=5759 GN=CL6EHI_088220 PE=4 SV=1	0	8.47	4	3	4	3	910	103. 5	5.81	6.59	3	0	
High	Q1EQ5 7	ADP-ribosylation factor-like protein 1 OS=Entamoeba histolytica OX=5759 GN=CL6EHI_194500 PE=3 SV=1	0	8.436	21	4	9	4	179	20.7	8.65	4.93	4	0	2077095.344
High	Q5NT2 8	Rab family GTPase (Fragment) OS=Entamoeba histolytica OX=5759 GN=EhRab2B PE=3 SV=1	0	8.405	15	4	9	1	213	23.8	6.23	9.6	4	3	6551986.734
High	A0A5K 1TWM 9	ADP-ribosylation factor OS=Entamoeba histolytica OX=5759 GN=CL6EHI_073470 PE=2 SV=1	0	8.404	17	3	6	3	174	19.9	6.58	3.45	3	0	871976.5391
High	A0A17 5JTS9	Thioredoxin domain-containing protein OS=Entamoeba	0	8.37	9	3	3	3	506	59.2	5.11	2.17	3	0	227931.1406

		histolytica OX=5759 GN=CL6EHI_072060 PE=4 SV=1												
High	A0A5K1VIN8	Nuclease domain containing protein OS=Entamoeba histolytica OX=5759 GN=CL6EHI_101240 PE=4 SV=1	0	8.344	5	4	5	4	843	95.6	8.59	4.32	4	0	526819.0156
High	A0A5K1UQN3	Carbohydrate kinase PfkB domain-containing protein OS=Entamoeba histolytica OX=5759 GN=CL6EHI_149860 PE=3 SV=1	0	8.321	4	2	4	2	691	75.7	5.9	1.87	2	0	421248.4219
High	A0A5K1UMY4	40S ribosomal protein s13 putative OS=Entamoeba histolytica OX=5759 GN=CL6EHI_035460 PE=3 SV=1	0	8.243	22	3	5	3	151	17	10.2	9.03	3	0	1057104.219
High	A0A5K1V3P4	Dolichyl-diphosphooligosaccharide--protein glycosyltransferase subunit 1 OS=Entamoeba histolytica OX=5759 GN=CL6EHI_093790 PE=3 SV=1	0	8.208	11	4	4	4	423	49.1	6.32	2.2	4	0	449285.5469
High	ENSEMBL:ENSBTAP00000014147	(Bos taurus) 12 kDa protein	0	8.206	32	3	12	3	113	11.7	6.55	2.15	3	0	22446685.83
High	A0A5K1U6Y6	Phosphoribulokinase uridine kinase family protein OS=Entamoeba histolytica OX=5759 GN=CL6EHI_087540 PE=4 SV=1	0	7.975	7	2	2	2	543	61.4	6.48	5.41	2	0	228427.2656

High	Q9BLE8	small GTPase Rab7A OS=Entamoeba histolytica OX=5759 GN=EhRab7A PE=2 SV=1	0	7.899	19	3	5	3	206	22.9	5.41	6.32	3	0	1833145.531
High	ENSEMBL:ENSBTAP00000024462	(Bos taurus) 47 kDa protein	0	7.79	8	3	4	3	419	46.5	6.33	7.38	3	0	356935.3438
High	A0A5K1TWL3	small monomeric GTPase OS=Entamoeba histolytica OX=5759 GN=CL6EHI_129750 PE=3 SV=1	0	7.739	23	4	8	3	201	22.1	6.21	7.26	4	1	1667321.844
High	A0A5K1UAM6	Polyadenylate-binding protein putative OS=Entamoeba histolytica OX=5759 GN=CL6EHI_198750 PE=4 SV=1	0	7.61	8	4	4	4	524	60.2	8.51	1.94	4	0	326481.5781
High	A0A5K1VRK7	Alcohol dehydrogenase putative OS=Entamoeba histolytica OX=5759 GN=CL6EHI_088020 PE=4 SV=1	0	7.563	8	3	4	2	382	42.5	7.21	5.89	3	0	203650.3438
High	A0A5K1VV61	AIG1 family protein OS=Entamoeba histolytica OX=5759 GN=CL6EHI_144270 PE=4 SV=1	0	7.55	12	3	7	3	287	32.9	8.43	3.86	3	0	1864714.875
High	A0A5K1U0M8	40S ribosomal protein S3 OS=Entamoeba histolytica OX=5759 GN=CL6EHI_146340 PE=2 SV=1	0	7.542	13	4	5	4	244	26.9	9.85	4.42	4	0	1227175.375

High	Q9NH04	Malic enzyme OS=Entamoeba histolytica OX=5759 GN=CL6EHI_044970 PE=1 SV=1	0	7.538	9	4	4	4	487	53.3	6.29	2.23	4	0	
High	A0A175JJ49	uridine/cytidine kinase OS=Entamoeba histolytica OX=5759 GN=CL6EHI_051730A PE=4 SV=1	0	7.472	21	4	6	4	261	30.6	6.81	4.09	4	0	1477844.156
High	A0A5K1TXY2	WD-repeat protein OS=Entamoeba histolytica OX=5759 GN=CL6EHI_198950 PE=2 SV=1	0	7.46	9	2	2	1	329	36.9	4.93	5.13	2	0	
High	A0A5K1VNR7	Uncharacterized protein OS=Entamoeba histolytica OX=5759 GN=CL6EHI_104580 PE=4 SV=1	0	7.434	16	4	11	4	251	29.2	10.21	7.4	4	0	4396976.375
High	A0A5K1U550	Uncharacterized protein OS=Entamoeba histolytica OX=5759 GN=CL6EHI_053850 PE=4 SV=1	0	7.37	6	4	6	4	652	76.4	9.22	2.45	4	0	1430238.813
High	A0A5K1US20	Arp2/3 complex 34 kDa subunit OS=Entamoeba histolytica OX=5759 GN=CL6EHI_091250 PE=3 SV=1	0	7.161	14	3	8	3	293	33.6	8.88	14.15	3	0	12004048.83
High	A0A5K1U6X0	non-specific serine/threonine protein kinase OS=Entamoeba histolytica OX=5759 GN=CL6EHI_050140 PE=3 SV=1	0	7.127	8	2	2	2	302	35.5	9.26	2.31	2	0	112831.3359
High	A0A5K1VA91	Heat repeat domain containing protein OS=Entamoeba histolytica OX=5759 GN=CL6EHI_040260 PE=3 SV=1	0	7.026	5	4	6	4	1074	122.5	7.58	1.94	4	0	1413729.016

High	A0A5K1UYX0	Phosphoglycerate kinase OS=Entamoeba histolytica OX=5759 GN=CL6EHI_188180 PE=3 SV=1	0	7.002	7	2	2	2	415	44.8	6.79	4.14	2	0	325332.9688
High	A0A5K1UCI2	Receptor mediated endocytosis protein putative OS=Entamoeba histolytica OX=5759 GN=CL6EHI_105270 PE=4 SV=1	0	6.99	7	3	5	1	507	57.6	7.4	8.22	3	0	894156.8125
High	A0A5K1TZQ4	EH-domain containing protein putative OS=Entamoeba histolytica OX=5759 GN=CL6EHI_152680 PE=4 SV=1	0	6.864	6	3	6	1	508	57.6	7.08	4.3	3	1	631652.0625
High	A0A5K1V659	Histidine acid phosphatase family protein OS=Entamoeba histolytica OX=5759 GN=CL6EHI_039860 PE=3 SV=1	0	6.825	4	3	6	3	420	48.8	6.68	6.54	3	0	1293300.539
High	A0A5K1VQK9	3 exoribonuclease family protein OS=Entamoeba histolytica OX=5759 GN=CL6EHI_040320 PE=3 SV=1	0	6.8	7	1	4	1	238	26.6	5.27	10.56	1	0	
High	A0A5K1TVJ4	Calponin homology domain protein putative OS=Entamoeba histolytica OX=5759 GN=CL6EHI_199000 PE=4 SV=1	0	6.791	5	3	5	3	619	69.8	4.92	6.09	3	0	495413.9297
High	Q5NT18	Rab family GTPase (Fragment) OS=Entamoeba histolytica OX=5759 GN=EhRabM3 PE=3 SV=1	0	6.781	13	2	2	2	204	22.5	4.67	2.55	2	0	392642.9063
High	A0A5K1VBQ1	RNAseI inhibitor-like protein putative OS=Entamoeba histolytica OX=5759 GN=CL6EHI_156200 PE=4 SV=1	0	6.755	4	3	6	3	651	73.6	8.76	6.93	3	0	641244.6875

High	A0A5K1V6W1	Aminoacyl-histidine dipeptidase putative OS=Entamoeba histolytica OX=5759 GN=CL6EHI_127280 PE=4 SV=1	0	6.752	5	2	7	2	505	56.3	5.44	2.46	2	0	210324.3594
High	A0A175JQJ5	Phospholipid/glycerol acyltransferase domain-containing protein OS=Entamoeba histolytica OX=5759 GN=CL6EHI_163570A PE=4 SV=1	0	6.655	10	2	2	2	269	31.2	8.85	2.37	2	0	380893.9688
High	A0A5K1URF7	Leukocyte-endothelial cell adhesion molecule 3 putative OS=Entamoeba histolytica OX=5759 GN=CL6EHI_201200 PE=2 SV=1	0	6.518	6	2	8	2	312	35.9	5.08	2.68	2	0	317463.5625
High	P02769	SWISS-PROT:P02769 (Bos taurus) Bovine serum albumin precursor	0	6.491	4	3	4	1	607	69.2	6.18	10.12	3	0	111131.2734
High	A0A5K1UJU1	Aspartate ammonia-lyase putative OS=Entamoeba histolytica OX=5759 GN=CL6EHI_150390 PE=4 SV=1	0	6.477	6	3	7	3	472	52.3	6.23	12.25	3	0	646787.625
High	A0A5K1U0I1	Peptidase C1A papain C-terminal domain-containing protein OS=Entamoeba histolytica OX=5759 GN=CL6EHI_093970 PE=4 SV=1	0	6.448	5	2	2	2	595	68.8	5.36	2.12	2	0	90992.03125
High	A0A5K1VAZ1	Rubrerythrin putative OS=Entamoeba histolytica OX=5759 GN=CL6EHI_134810 PE=4 SV=1	0	6.402	6	1	4	1	189	21.2	6.39	4.48	1	0	1369627.063

High	A0A5K1VSB3	26S protease regulatory subunit putative OS=Entamoeba histolytica OX=5759 GN=CL6EHI_194570 PE=3 SV=1	0	6.376	8	2	4	1	391	44.2	7.66	5.33	2	0	
High	A0A5K1VC31	Ras family GTPase OS=Entamoeba histolytica OX=5759 GN=CL6EHI_198330 PE=2 SV=1	0	6.331	13	2	3	2	207	22.8	6.01	7.07	2	0	693118.5
High	A0A5K1VIE7	Elongation factor 2 OS=Entamoeba histolytica OX=5759 GN=CL6EHI_189490 PE=4 SV=1	0	6.329	5	5	6	5	841	93.3	6.28	1.85	5	0	3978007.25
High	A0A5K1ULH3	T-complex protein 1 subunit alpha OS=Entamoeba histolytica OX=5759 GN=CL6EHI_155220 PE=3 SV=1	0	6.287	6	3	4	3	544	59.1	6.02	6.12	3	0	252422.8906
High	A0A5K1UPF5	Zinc finger, c2h2 type domain containing protein OS=Entamoeba histolytica OX=5759 GN=CL6EHI_158220 PE=4 SV=1	0	6.274	9	3	5	3	371	42.2	8.06	7.56	3	0	1920871.93
High	Q9BLF2	Rab family GTPase OS=Entamoeba histolytica OX=5759 GN=EhRabC1 PE=2 SV=1	0	6.181	22	3	4	2	204	22.4	7.2	5.68	3	1	893047.3906
High	A0A5K1V6X5	WH2 domain-containing protein OS=Entamoeba histolytica OX=5759 GN=CL6EHI_016130 PE=4 SV=1	0	6.147	2	2	2	2	510	55.9	7.2	2.66	2	0	1264412.625
High	A0A175JI50	Uncharacterized protein OS=Entamoeba histolytica	0	6.085	2	3	5	1	1153	130.9	6.55	5.1	3	2	1636850.281

		OX=5759 GN=CL6EHI_198620 PE=4 SV=1												
High	A0A5K1U1K6	sulfate adenylyltransferase OS=Entamoeba histolytica OX=5759 GN=CL6EHI_197160 PE=3 SV=1	0	5.927	5	2	3	2	423	47.9	9.07	5.76	2	0	312099.5313
High	A0A5K1UHJ8	Rhodanese domain-containing protein OS=Entamoeba histolytica OX=5759 GN=CL6EHI_175400 PE=4 SV=1	0	5.927	4	3	7	3	919	108.2	5.76	3.8	3	0	451366.1563
High	A0A5K1U271	Grainin putative OS=Entamoeba histolytica OX=5759 GN=CL6EHI_120360 PE=4 SV=1	0	5.913	11	2	4	2	215	24.6	7.18	7.67	2	0	934414.4063
High	A0A5K1VCJ7	40S ribosomal protein s16 putative OS=Entamoeba histolytica OX=5759 GN=CL6EHI_038620 PE=3 SV=1	0	5.883	23	3	3	3	158	17.5	10.02	4.62	3	0	182722.5313
High	A0A5K1VR88	proton-translocating NAD(P)(+) transhydrogenase OS=Entamoeba histolytica OX=5759 GN=CL6EHI_055400 PE=4 SV=1	0	5.881	3	3	4	3	1098	119	5.86	5.4	3	0	2281952.25
High	A0A175JKZ9	Pyrophosphate--fructose 6-phosphate 1-phosphotransferase OS=Entamoeba histolytica OX=5759 GN=CL6EHI_000730 PE=3 SV=1	0	5.827	5	2	2	2	546	60.1	6.79	2.01	2	0	
High	A0A5K1VQN1	Proteasome regulatory subunit putative OS=Entamoeba histolytica OX=5759 GN=CL6EHI_179970 PE=3 SV=1	0	5.698	7	3	3	3	473	54.2	7.9	0	3	0	596417.4219

High	A0A5K1VUH5	3'-5' exonuclease domain-containing protein OS=Entamoeba histolytica OX=5759 GN=CL6EHI_148670 PE=4 SV=1	0	5.681	12	3	4	3	279	32	6.92	2.9	3	0	799540.1563
High	A0A5K1VCU5	NadR/Ttd14 AAA domain-containing protein OS=Entamoeba histolytica OX=5759 GN=CL6EHI_095850 PE=4 SV=1	0	5.558	11	2	5	2	240	27.3	5.85	2.17	2	0	2076351.5
High	A0A175K126	Ribosomal protein L1 OS=Entamoeba histolytica OX=5759 GN=CL6EHI_201830 PE=4 SV=1	0	5.515	6	1	1	1	289	33.2	9.35	0	1	0	
High	A0A5K1TUH8	Phosphatidylinositol 4 phosphate 5-kinase putative OS=Entamoeba histolytica OX=5759 GN=CL6EHI_153770 PE=4 SV=1	0	5.504	5	3	3	3	646	74.3	5.76	1.76	3	0	214084.0938
High	A0A5K1VUH4	Actin putative OS=Entamoeba histolytica OX=5759 GN=CL6EHI_008780 PE=3 SV=1	0	5.49	5	3	4	3	763	84.7	6.11	5.07	3	0	479679.9063
High	Q5D862	SWISS-PROT:Q5D862 Tax_Id=9606 Gene_Symbol=FLG2 Filaggrin-2	0	5.429	1	1	1	1	2391	247.9	8.31	2.55	1	0	92631.17969
High	A0A5K1TUY8	Alcohol dehydrogenase putative OS=Entamoeba histolytica OX=5759 GN=CL6EHI_125950 PE=4 SV=1	0	5.419	6	3	5	2	383	42.1	7.05	4.03	3	0	436001.3438
High	A0A5K1UMV9	Uncharacterized protein OS=Entamoeba histolytica	0	5.404	6	2	2	2	440	51.1	5.39	2.88	2	0	

		OX=5759 GN=CL6EHI_151200 PE=4 SV=1												
High	S0AWN0	EF-hand calcium-binding domain containing protein OS=Entamoeba histolytica OX=5759 GN=CL6EHI_060740 PE=2 SV=1	0	5.39	9	2	12	2	227	26.6	7.27	10.46	2	0	35658723.13
High	A0A175JXV0	Coronin OS=Entamoeba histolytica OX=5759 GN=CL6EHI_083590B PE=3 SV=1	0	5.271	7	2	4	2	304	33.7	6.52	7.17	2	0	2178776.141
High	A0A175JJ87	Uncharacterized protein OS=Entamoeba histolytica OX=5759 GN=CL6EHI_166920 PE=4 SV=1	0	5.226	2	3	5	1	1151	130.8	6.58	2.19	3	0	
High	A0A175JUB5	Adapter-related protein complex 3 p 3 subunit putative OS=Entamoeba histolytica OX=5759 GN=CL6EHI_083430 PE=3 SV=1	0	5.134	2	2	4	2	855	97.8	4.67	1.65	2	0	602069.9688
High	A0A5K1VTL5	dolichyl-diphosphooligosaccharide--protein glycotransferase OS=Entamoeba histolytica OX=5759 GN=CL6EHI_011940 PE=3 SV=1	0	5.082	3	2	3	2	721	82.9	8.65	6.89	2	0	202890.5156
High	Q6AW60	EhRab11A protein OS=Entamoeba histolytica OX=5759 GN=EhRAB11A PE=2 SV=1	0	5.07	15	4	22	1	207	23.3	5.15	17.95	4	0	
High	A0A5K1UZD0	protein disulfide-isomerase OS=Entamoeba histolytica	0	5.06	8	3	3	3	368	41.7	7.99	3.88	3	0	807624.625

		OX=5759 GN=CL6EHI_071590 PE=3 SV=1													
High	A0A5K1TXA3	START domain-containing protein OS=Entamoeba histolytica OX=5759 GN=CL6EHI_173480 PE=4 SV=1	0	4.983	5	2	4	2	240	27.6	7.77	4.16	2	0	
High	A0A5K1TUI0	T-complex protein 1 subunit epsilon OS=Entamoeba histolytica OX=5759 GN=CL6EHI_155450 PE=3 SV=1	0	4.979	3	2	2	2	536	58.5	6.04	0	2	0	232321.7188
High	A0A5K1U480	Histidine acid phosphatase family protein OS=Entamoeba histolytica OX=5759 GN=CL6EHI_146950 PE=2 SV=1	0	4.974	6	2	2	2	418	49.3	5.45	2.53	2	0	80385.57813
High	A0A5K1VJV7	SH3 domain-containing protein OS=Entamoeba histolytica OX=5759 GN=CL6EHI_026470 PE=4 SV=1	0	4.954	6	3	3	3	375	43.1	5.68	0	3	0	178362.9375
High	A0A5K1UFS8	Long-chain-fatty-acid--coa ligase putative OS=Entamoeba histolytica OX=5759 GN=CL6EHI_147010 PE=3 SV=1	0	4.95	3	2	3	2	682	77.3	6.55	0	2	0	583305.25
High	Q963D1	Rab family GTPase OS=Entamoeba histolytica OX=5759 GN=rab8 PE=2 SV=1	0	4.949	11	2	4	1	199	22.3	5.16	7.07	2	0	1809056.5
High	A0A5K1U0N3	60S ribosomal protein L7 putative OS=Entamoeba histolytica OX=5759 GN=CL6EHI_146530 PE=3 SV=1	0	4.948	11	3	5	3	230	25.7	10.02	7.51	3	0	1126594.188
High	A0A5K1UTZ9	Uncharacterized protein OS=Entamoeba histolytica	0	4.859	12	2	2	2	224	25.2	5.1	2.56	2	0	

		OX=5759 GN=CL6EHI_073160 PE=4 SV=1												
High	A0A5K1UEB0	F-BAR domain-containing protein OS=Entamoeba histolytica OX=5759 GN=CL6EHI_065490 PE=4 SV=1	0	4.821	6	2	4	2	378	43.3	5	1.77	2	0	428379.375
High	A0A5K1VTE2	Oxidation resistance protein 1 OS=Entamoeba histolytica OX=5759 GN=CL6EHI_134660 PE=3 SV=1	0	4.813	7	2	2	2	359	41.7	4.69	3.95	2	0	212961.625
High	A0A5K1UCY1	Ribosomal protein L11 putative OS=Entamoeba histolytica OX=5759 GN=CL6EHI_124300 PE=3 SV=1	0	4.783	19	3	14	3	174	19.6	9.54	2.8	3	0	1622345.172
High	A0A5K1UV23	40S ribosomal protein s15a putative OS=Entamoeba histolytica OX=5759 GN=CL6EHI_073600 PE=3 SV=1	0	4.725	24	3	5	3	130	14.7	9.8	4.45	3	0	
High	A0A5K1TUE3	Endoplasmic reticulum transmembrane protein OS=Entamoeba histolytica OX=5759 GN=CL6EHI_195140 PE=3 SV=1	0	4.707	15	2	2	2	152	17.7	8.27	0	2	0	113007.4609
High	A0A5K1ULU2	small monomeric GTPase OS=Entamoeba histolytica OX=5759 GN=CL6EHI_181250 PE=3 SV=1	0	4.701	16	3	34	3	196	21.8	7.56	18.29	3	0	4902185.547
High	A0A5K1V873	Triosephosphate isomerase OS=Entamoeba histolytica OX=5759 GN=CL6EHI_056480 PE=3 SV=1	0	4.701	11	2	3	2	261	28.1	6.55	0	2	0	478330.4375

High	A0A5K1TZ40	Signal recognition particle receptor subunit beta OS=Entamoeba histolytica OX=5759 GN=CL6EHI_000670 PE=3 SV=1	0	4.675	10	2	2	2	236	26.3	6	2.02	2	0	188665.3438
High	A0A5K1VEM6	Proteasome subunit beta OS=Entamoeba histolytica OX=5759 GN=CL6EHI_078710 PE=3 SV=1	0	4.658	10	2	5	2	198	22.3	6.2	9.19	2	0	184271.4531
High	A0A5K1VIV7	Skp1 family protein OS=Entamoeba histolytica OX=5759 GN=CL6EHI_066770 PE=3 SV=1	0	4.525	16	2	4	2	158	18.2	4.67	2.24	2	0	272301.875
High	A0A5K1U279	Eukaryotic translation initiation factor 6 OS=Entamoeba histolytica OX=5759 GN=EIF6 PE=3 SV=1	0	4.499	10	2	9	2	239	25.9	4.79	15.93	2	0	54423031.19
High	A0A060N0F8	Antigen OS=Entamoeba histolytica OX=5759 GN=CL6EHI_004370 PE=2 SV=1	0	4.491	7	2	2	2	335	39.2	5.68	2.51	2	0	264802.5313
High	A0A5K1UPI5	T-complex protein 1 subunit zeta OS=Entamoeba histolytica OX=5759 GN=CL6EHI_125800 PE=3 SV=1	0	4.471	5	2	3	2	540	59.9	6.39	4.14	2	0	
High	A0A5K1U6T1	Ribosome biogenesis protein WDR12 homolog OS=Entamoeba histolytica OX=5759 GN=CL6EHI_118030 PE=3 SV=1	0	4.465	7	2	4	2	406	44.8	6.98	8.51	2	0	2970523.75
High	A0A5K1VM78	Chaperone proteinDNAj putative OS=Entamoeba	0	4.408	6	1	3	1	284	31.2	8.75	0	1	0	

		histolytica OX=5759 GN=CL6EHI_049400 PE=4 SV=1													
High	A0A5K1TWJ3	Metallo-beta-lactamase superfamily protein OS=Entamoeba histolytica OX=5759 GN=CL6EHI_107070 PE=3 SV=1	0	4.333	3	1	2	1	622	71	6.39	5.4	1	0	
High	A0A5K1UKP7	Galactose-inhibitable lectin small subunit putative OS=Entamoeba histolytica OX=5759 GN=CL6EHI_159870 PE=2 SV=1	0	4.33	8	3	3	3	257	30.8	5.72	3.27	3	0	1784356.156
High	A0A5K1U033	DNA/RNA-binding protein Alba-like domain-containing protein OS=Entamoeba histolytica OX=5759 GN=CL6EHI_006070 PE=4 SV=1	0	4.314	9	1	3	1	223	26.1	9.99	7	1	0	507506.6875
High	Q9BLF3	Rab family GTPase OS=Entamoeba histolytica OX=5759 GN=EhRab1A PE=2 SV=1	0	4.311	10	2	3	1	205	22.9	6.24	5.85	2	0	718086.8125
High	A0A175JJ28	Signal recognition particle subunit SRP72 OS=Entamoeba histolytica OX=5759 GN=CL6EHI_131200 PE=4 SV=1	0	4.304	5	1	1	1	257	29.5	9.54	2.67	1	0	
High	A0A5K1VI00	V-type proton ATPase subunit OS=Entamoeba histolytica OX=5759 GN=CL6EHI_106350 PE=3 SV=1	0	4.286	8	3	4	3	352	40.3	4.67	5.8	3	0	407809.6875
High	A0A5K1VSI7	Uncharacterized protein OS=Entamoeba histolytica	0	4.275	2	2	5	2	909	101.9	5.33	2.07	2	0	277580.5313

		OX=5759 GN=CL6EHI_049680 PE=3 SV=1													
High	Q76KF5	D 3 phosphoglycerate dehydrogenase putative OS=Entamoeba histolytica OX=5759 GN=EhPGDH PE=1 SV=1	0	4.27	10	3	3	3	299	33.4	8.09	2.11	3	0	252878.7031
High	Q6AW67	EhRab7B protein OS=Entamoeba histolytica OX=5759 GN=EhRAB7B PE=3 SV=1	0	4.257	11	2	2	1	207	23.1	5.66	3.8	2	0	
High	A0A5K1VQD4	proteasome endopeptidase complex OS=Entamoeba histolytica OX=5759 GN=CL6EHI_148040 PE=4 SV=1	0	4.227	7	2	4	2	262	28.6	6.99	1.85	2	0	375373.2813
High	A0A5K1UF17	26S protease regulatory subunit putative OS=Entamoeba histolytica OX=5759 GN=CL6EHI_180350 PE=3 SV=1	0	4.203	5	2	4	2	410	46.3	6.49	0	2	0	1704566.5
High	A0A5K1TVM3	Signal recognition particle receptor alpha subunit OS=Entamoeba histolytica OX=5759 GN=CL6EHI_200840 PE=3 SV=1	0	4.198	6	2	2	2	488	54.1	8.57	3.98	2	0	205154.25
High	A0A175JYK2	Clathrin coat assembly protein putative OS=Entamoeba histolytica OX=5759 GN=CL6EHI_135430 PE=3 SV=1	0	4.182	7	3	4	3	414	47	9.26	4.31	3	0	3115965.281
High	A0A5K1VQU7	C2 NT-type domain-containing protein OS=Entamoeba histolytica OX=5759 GN=CL6EHI_192140 PE=4 SV=1	0	4.146	3	1	1	1	581	66.5	8.43	0	1	0	92148.73438

High	A0A5K 1VDV9	SHSP domain-containing protein OS=Entamoeba histolytica OX=5759 GN=CL6EHI_184220 PE=3 SV=1	0	4.139	4	3	3	3	512	61.2	5.21	2.68	3	0	208779.0313
High	A0A5K 1V7V8	Phosphoglycerate mutase family protein. putative OS=Entamoeba histolytica OX=5759 GN=CL6EHI_129820 PE=4 SV=1	0	4.123	10	2	5	2	205	22.9	6.79	4.93	2	0	421013.3125
High	A0A5K 1UT51	Malate dehydrogenase OS=Entamoeba histolytica OX=5759 GN=CL6EHI_030810 PE=3 SV=1	0	4.111	7	2	2	2	355	39.3	6.54	3.38	2	0	893014.875
High	A0A5K 1VPW 7	Centromere microtubule binding protein cbf5 putative OS=Entamoeba histolytica OX=5759 GN=CL6EHI_115300 PE=3 SV=1	0	4.092	2	2	3	2	484	54.1	8.21	0	2	0	251799.2813
High	A0A5K 1TWS3	3-ketoacyl-CoA synthase OS=Entamoeba histolytica OX=5759 GN=CL6EHI_111000 PE=3 SV=1	0	4.07	4	2	2	2	494	56.3	8.69	2.24	2	0	
High	S0AV4 2	Malate dehydrogenase putative OS=Entamoeba histolytica OX=5759 GN=CL6EHI_092450 PE=2 SV=1	0	3.994	6	2	4	2	370	40.5	6.71	1.77	2	0	489464.9063
High	A0A5K 1UUS7	Proteasome subunit alpha type OS=Entamoeba histolytica OX=5759 GN=CL6EHI_153190 PE=3 SV=1	0	3.948	9	2	3	2	242	26.9	6.29	3.76	2	0	367509.9688
High	A0A5K 1U299	40S ribosomal protein s10 putative OS=Entamoeba	0	3.887	12	2	3	2	127	14.5	8.95	0	2	0	132342.25

		histolytica OX=5759 GN=CL6EHI_197030 PE=3 SV=1													
High	A0A5K1VSQ4	Estradiol 17-beta-dehydrogenase putative OS=Entamoeba histolytica OX=5759 GN=CL6EHI_165070 PE=3 SV=1	0	3.854	3	2	3	2	323	36.6	9.04	6.8	2	0	1260572.563
High	A0A5K1UHL7	NAD-dependent epimerase/dehydratase domain-containing protein OS=Entamoeba histolytica OX=5759 GN=CL6EHI_098800 PE=4 SV=1	0	3.849	8	3	3	3	365	40.9	6.44	2.04	3	0	648223.4688
High	A0A5K1U8H1	Threonylcarbamoyladenosine tRNA methylthiotransferase OS=Entamoeba histolytica OX=5759 GN=CL6EHI_141830 PE=3 SV=1	0	3.819	3	1	2	1	438	48.9	6.58	2.42	1	0	211889.875
High	A0A5K1VQI3	uridine/cytidine kinase OS=Entamoeba histolytica OX=5759 GN=CL6EHI_196580 PE=4 SV=1	0	3.787	8	2	4	2	257	29.5	6.15	1.82	2	0	579699.7813
High	A0A5K1USN1	F-actin-capping protein subunit beta OS=Entamoeba histolytica OX=5759 GN=CL6EHI_005020 PE=3 SV=1	0	3.783	4	2	3	2	276	31.4	5.05	1.99	2	0	970354.5625
High	A0A5K1UC53	Serine/threonine-protein phosphatase OS=Entamoeba histolytica OX=5759 GN=CL6EHI_117570 PE=3 SV=1	0	3.761	4	1	1	1	433	48.9	5.08	2.64	1	0	
High	A0A5K1UCT7	Rhogap domain containing protein OS=Entamoeba	0	3.759	10	3	3	3	301	33.9	6.15	1.69	3	0	261410.7656

		histolytica OX=5759 GN=CL6EHI_172130 PE=4 SV=1												
High	A0A5K1V9U3	Uncharacterized protein OS=Entamoeba histolytica OX=5759 GN=CL6EHI_035730 PE=4 SV=1	0	3.748	4	2	2	2	679	78.9	6.74	1.84	2	0	62747.96484
High	A0A5K1VC06	F-actin-capping protein subunit alpha OS=Entamoeba histolytica OX=5759 GN=CL6EHI_033740 PE=2 SV=1	0	3.744	6	1	2	1	270	30.5	7.72	2.32	1	0	293049.3125
High	A0A5K1U481	Autophagy protein 5 OS=Entamoeba histolytica OX=5759 GN=CL6EHI_022880 PE=3 SV=1	0	3.72	10	3	4	3	288	33.5	4.98	1.67	3	0	
High	A0A5K1VIF4	40S ribosomal protein S7 OS=Entamoeba histolytica OX=5759 GN=CL6EHI_067530 PE=3 SV=1	0	3.72	9	2	2	1	201	21.9	10.18	1.84	2	1	360198.6875
High	Q1RMK2	TREMBL:Q1RMK2 (Bos taurus) IGHM protein	0	3.714	3	2	2	2	597	65	5.97	1.68	2	0	1111515.914
High	A0A5K1VE83	Geranylgeranyl pyrophosphate synthase putative OS=Entamoeba histolytica OX=5759 GN=CL6EHI_105060 PE=3 SV=1	0	3.711	5	2	8	2	349	40	5.66	0	2	0	1774991.594
High	A0A5K1U3R2	Actin-related protein 2/3 complex subunit OS=Entamoeba histolytica OX=5759 GN=CL6EHI_094120 PE=3 SV=1	0	3.689	11	3	6	3	366	40.5	6.83	0	3	0	194713.9688
High	A0A5K1UEP5	Ribosomal protein L19 OS=Entamoeba histolytica	0	3.661	7	2	4	2	198	22.9	10.76	3.29	2	0	624953.3281

		OX=5759 GN=CL6EHI_096850 PE=3 SV=1													
High	A0A5K1TX85	40S ribosomal protein s2 putative OS=Entamoeba histolytica OX=5759 GN=CL6EHI_020280 PE=3 SV=1	0	3.654	8	2	3	2	255	27.7	10.14	0	2	0	370443.25
High	A0A5K1VPW6	26S protease regulatory subunit putative OS=Entamoeba histolytica OX=5759 GN=CL6EHI_053020 PE=3 SV=1	0	3.602	3	1	1	1	422	47.3	5.54	3.37	1	0	
High	A0A5K1U4R4	40S ribosomal protein S25 OS=Entamoeba histolytica OX=5759 GN=CL6EHI_074800 PE=3 SV=1	0	3.589	20	3	4	3	137	14.7	10.14	3.99	3	0	
High	A0A5K1VTJ5	VPS9 domain-containing protein OS=Entamoeba histolytica OX=5759 GN=CL6EHI_163600 PE=4 SV=1	0	3.579	2	1	3	1	628	71.4	6.24	0	1	0	131734.125
High	A0A5K1URT5	Leucine-rich repeat containing protein OS=Entamoeba histolytica OX=5759 GN=CL6EHI_140310 PE=4 SV=1	0	3.549	2	1	2	1	663	75.9	6.55	2.67	1	0	
High	A0A5K1UFV7	Ribosomal protein s18 putative OS=Entamoeba histolytica OX=5759 GN=CL6EHI_009680 PE=3 SV=1	0	3.547	17	3	4	3	155	17.7	10.48	1.85	3	0	1770955.578
High	A0A175JRI2	J domain-containing protein OS=Entamoeba histolytica OX=5759 GN=CL6EHI_200560 PE=4 SV=1	0	3.54	1	2	3	2	1927	220.3	6.49	3.89	2	0	263801.5313
High	A0A5K1VJP9	60S ribosomal protein L3 putative OS=Entamoeba	0	3.501	8	2	3	2	402	45.5	10.29	0	2	0	113677.1094

		histolytica OX=5759 GN=CL6EHI_005890 PE=2 SV=1												
High	A0A5K1VP89	Dolichol-phosphate mannosyltransferase subunit 1 OS=Entamoeba histolytica OX=5759 GN=CL6EHI_054260 PE=3 SV=1	0	3.5	6	1	1	1	353	38.8	8.21	3.98	1	0	
High	A0A5K1V9X5	Calcium-regulated actin-bundling protein C-terminal domain-containing protein OS=Entamoeba histolytica OX=5759 GN=CL6EHI_086690 PE=4 SV=1	0	3.467	8	2	3	2	382	42	5.82	5.73	2	0	
High	A0A5K1VA88	Sulfotransferase OS=Entamoeba histolytica OX=5759 GN=CL6EHI_166030 PE=2 SV=1	0	3.434	3	1	2	1	441	51.7	6.07	4.7	1	0	
High	A0A175JDQ4	Uncharacterized protein OS=Entamoeba histolytica OX=5759 GN=CL6EHI_148770B PE=4 SV=1	0	3.432	5	1	2	1	245	29.8	9.06	6.38	1	0	689747.4375
High	A0A5K1TWR0	Heat repeat domain containing protein OS=Entamoeba histolytica OX=5759 GN=CL6EHI_064450 PE=3 SV=1	0	3.419	1	1	3	1	1062	119.9	4.91	0	1	0	
High	A0A175K1Z8	Clathrin heavy chain putative OS=Entamoeba histolytica OX=5759 GN=CL6EHI_201710 PE=4 SV=1	0	3.409	4	2	2	1	665	76	5.01	2.12	2	0	170547.375
High	A0A5K1VAX7	26S protease regulatory subunit putative OS=Entamoeba histolytica OX=5759 GN=CL6EHI_177320 PE=3 SV=1	0	3.37	5	2	3	2	389	43.8	7.61	2.18	2	0	383312.7813

High	A0A5K1U235	threonine ammonia-lyase OS=Entamoeba histolytica OX=5759 GN=CL6EHI_126080 PE=3 SV=1	0	3.365	3	1	2	1	426	45.2	7.06	5.09	1	0	929645.1875
High	A0A5K1UD09	small monomeric GTPase OS=Entamoeba histolytica OX=5759 GN=CL6EHI_015440 PE=3 SV=1	0	3.335	8	2	4	2	215	23.8	6.77	2.03	2	0	
High	A0A5K1UV56	Tartrate dehydrogenase putative OS=Entamoeba histolytica OX=5759 GN=CL6EHI_143560 PE=3 SV=1	0	3.313	4	1	1	1	370	41.1	5.77	2.46	1	0	
High	A0A5K1UAC0	non-specific serine/threonine protein kinase OS=Entamoeba histolytica OX=5759 GN=CL6EHI_044470 PE=3 SV=1	0	3.308	3	1	1	1	440	50.6	6.32	2.31	1	0	200760.6719
High	A0A5K1UQT1	Uncharacterized protein OS=Entamoeba histolytica OX=5759 GN=CL6EHI_011550 PE=4 SV=1	0	3.287	2	1	1	1	615	69.9	6.89	2.56	1	0	
High	A0A5K1UJ17	acetyl-CoA carboxytransferase OS=Entamoeba histolytica OX=5759 GN=CL6EHI_082530 PE=4 SV=1	0	3.276	1	2	2	2	1244	137.9	6.74	0	2	0	302503.0938
High	A0A5K1VNR3	40S ribosomal protein S7 OS=Entamoeba histolytica OX=5759 GN=CL6EHI_140540 PE=3 SV=1	0	3.273	9	2	3	1	196	21.5	10.18	1.84	2	0	109920.1953
High	Q9U8X2	serine O-acetyltransferase OS=Entamoeba histolytica OX=5759 GN=EhSAT PE=1 SV=1	0	3.195	6	2	2	2	305	34.4	7.08	0	2	0	1049344.75

High	A0A5K1UXM1	Uncharacterized protein OS=Entamoeba histolytica OX=5759 GN=CL6EHI_057830 PE=4 SV=1	0	3.154	5	2	3	2	366	42.6	8.95	1.68	2	0	500208.4844
High	A0A5K1U5U1	Alcohol dehydrogenase putative OS=Entamoeba histolytica OX=5759 GN=CL6EHI_166490 PE=4 SV=1	0	3.147	5	2	2	2	419	46.6	6.58	1.72	2	0	
High	A0A5K1UP32	GTP-binding nuclear protein OS=Entamoeba histolytica OX=5759 GN=CL6EHI_148190 PE=3 SV=1	0	3.146	12	2	2	2	219	25	8.6	1.74	2	0	
High	A0A5K1VI74	Superoxide dismutase OS=Entamoeba histolytica OX=5759 GN=CL6EHI_159160 PE=3 SV=1	0	3.138	11	2	3	2	190	22	6.79	1.66	2	0	1974507.344
High	S0AXC7	Endoplasmic reticulum-Golgi intermediate compartment protein 3 OS=Entamoeba histolytica OX=5759 GN=CL6EHI_182970 PE=2 SV=1	0	3.133	3	1	1	1	361	41.1	6.79	0	1	0	
High	A0A5K1VFE0	Deoxycytidine triphosphate deaminase putative OS=Entamoeba histolytica OX=5759 GN=CL6EHI_140240 PE=4 SV=1	0	3.124	6	1	1	1	171	19.4	6.19	2.21	1	0	
High	A0A5K1TUN7	Long-chain-fatty-acid--coa ligase putative OS=Entamoeba histolytica OX=5759 GN=CL6EHI_029050 PE=3 SV=1	0	3.116	3	2	3	2	613	68.9	6.9	2.47	2	0	953360.7656
High	A0A5K1UKP5	Elongation factor 2 kinase putative OS=Entamoeba	0	3.096	3	1	1	1	402	46.1	5.97	0	1	0	

		histolytica OX=5759 GN=CL6EHI_090240 PE=4 SV=1													
High	A0A5K1VD18	D-hydantoinase putative OS=Entamoeba histolytica OX=5759 GN=CL6EHI_024420 PE=3 SV=1	0	3.075	3	1	1	1	460	50.6	5.45	2.9	1	0	
High	A0A5K1U9I4	Variant sh3 domain containing protein OS=Entamoeba histolytica OX=5759 GN=CL6EHI_148050 PE=2 SV=1	0	3.02	3	1	1	1	393	43.9	5.05	0	1	0	
High	A0A5K1UDE6	Heat repeat domain containing protein OS=Entamoeba histolytica OX=5759 GN=CL6EHI_146520 PE=4 SV=1	0	2.931	3	2	3	2	715	81.4	7.99	2.12	2	0	160379.4375
High	A0A5K1U5E2	VHS domain-containing protein OS=Entamoeba histolytica OX=5759 GN=CL6EHI_100460 PE=4 SV=1	0	2.916	3	1	1	1	360	42.1	9.29	0	1	0	331326.75
High	A0A5K1UXW5	START domain-containing protein OS=Entamoeba histolytica OX=5759 GN=CL6EHI_006670 PE=4 SV=1	0	2.907	4	2	2	2	264	31.4	5.47	1.76	2	0	930554.6875
High	A0A5K1VL35	3-ketoacyl-CoA synthase OS=Entamoeba histolytica OX=5759 GN=CL6EHI_092190 PE=3 SV=1	0	2.865	4	1	1	1	487	55.8	8.59	0	1	0	
High	Q6Y0Y5	Vacuolar protein sorting 35 OS=Entamoeba histolytica OX=5759 GN=Vps35-1 PE=3 SV=2	0	2.858	2	2	3	2	757	87.1	5.74	1.67	2	0	173279.6719
High	A0A5K1TU85	non-specific serine/threonine protein kinase OS=Entamoeba	0	2.858	6	1	1	1	354	39.9	4.7	0	1	0	

		histolytica OX=5759 GN=CL6EHI_156670 PE=3 SV=1													
High	A0A5K1V6P1	Ribosomal protein s17 putative OS=Entamoeba histolytica OX=5759 GN=CL6EHI_135060 PE=3 SV=1	0	2.853	9	1	2	1	117	13.6	10.1	4.65	1	0	683654.5
High	A0A5K1UP90	Calcium-transporting ATPase OS=Entamoeba histolytica OX=5759 GN=CL6EHI_030830 PE=3 SV=1	0	2.849	1	1	1	1	1026	113.9	5.47	0	1	0	105968.0391
High	A0A5K1UZE7	Heat shock protein 90 putative OS=Entamoeba histolytica OX=5759 GN=CL6EHI_196940 PE=3 SV=1	0	2.817	2	1	1	1	718	82.9	5.03	0	1	0	
High	A0A5K1V1D8	RuvB-like helicase OS=Entamoeba histolytica OX=5759 GN=CL6EHI_040360 PE=3 SV=1	0	2.731	4	2	3	2	439	47.8	6.33	1.68	2	0	
High	A0A5K1UJX5	Mut nudix family protein OS=Entamoeba histolytica OX=5759 GN=CL6EHI_097930 PE=4 SV=1	0	2.66	10	2	3	2	209	23.7	5.34	1.76	2	0	1106530.938
High	A0A5K1UK83	Sh2-protein kinase domain containing protein OS=Entamoeba histolytica OX=5759 GN=CL6EHI_055990 PE=4 SV=1	0	2.65	3	2	3	2	495	55.6	6.48	5.99	2	0	798033.6875
High	A0A5K1V8P6	60S ribosomal protein L27 putative OS=Entamoeba histolytica OX=5759 GN=CL6EHI_146370 PE=3 SV=1	0	2.645	9	1	1	1	139	15.7	10.43	0	1	0	

High	A0A5K1UR19	Serine threonine-protein kinase putative OS=Entamoeba histolytica OX=5759 GN=CL6EHI_148200 PE=4 SV=1	0	2.628	5	3	6	3	582	67.4	8.09	1.61	3	0	234761.3125
High	A0A5K1U6A7	Thioredoxin domain-containing protein OS=Entamoeba histolytica OX=5759 GN=CL6EHI_160850 PE=4 SV=1	0	2.59	2	1	1	1	497	57.8	5.73	0	1	0	
High	A0A175JPR3	Uncharacterized protein OS=Entamoeba histolytica OX=5759 GN=CL6EHI_040340 PE=4 SV=1	0	2.589	6	1	1	1	205	24.1	9.35	0	1	0	
High	A0A5K1VGN6	Rab family GTPase OS=Entamoeba histolytica OX=5759 GN=CL6EHI_161030 PE=3 SV=1	0	2.571	10	2	4	2	212	23.8	5.29	0	2	0	
High	A0A5K1UKC9	Gem-associated protein 2 OS=Entamoeba histolytica OX=5759 GN=CL6EHI_131510 PE=4 SV=1	0	2.57	5	1	1	1	224	25.9	5.39	0	1	0	
High	A0A5K1UHM3	Aminopeptidase OS=Entamoeba histolytica OX=5759 GN=CL6EHI_008380 PE=3 SV=1	0	2.498	2	1	1	1	827	94.7	5.35	0	1	0	148733.3281
High	A0A5K1VNU0	nucleoside-diphosphate kinase OS=Entamoeba histolytica OX=5759 GN=CL6EHI_104360 PE=3 SV=1	0	2.489	6	1	1	1	211	23.7	6.98	0	1	0	
High	A0A5K1UMF6	small monomeric GTPase OS=Entamoeba histolytica OX=5759 GN=CL6EHI_052150 PE=3 SV=1	0	2.487	10	2	3	1	189	21.3	5.76	1.64	2	0	120510.4609

High	A0A5K1UG99	B30.2/SPRY domain-containing protein OS=Entamoeba histolytica OX=5759 GN=CL6EHI_116470 PE=4 SV=1	0	2.472	6	2	4	2	282	32.4	6.89	1.78	2	0	6076731.375
High	A0A5K1U3P0	Cysteine synthase a putative OS=Entamoeba histolytica OX=5759 GN=CL6EHI_160930 PE=4 SV=1	0	2.441	4	1	1	1	337	36.7	6.81	0	1	0	
High	A0A5K1VTZ3	Papain family cysteine protease domain containing protein OS=Entamoeba histolytica OX=5759 GN=CL6EHI_050800 PE=4 SV=1	0	2.43	2	1	1	1	567	64.5	5.41	2.02	1	0	130040.5625
High	A0A5K1UXY9	Heat repeat domain containing protein OS=Entamoeba histolytica OX=5759 GN=CL6EHI_124530 PE=2 SV=1	0	2.416	4	1	1	1	308	34.3	5.26	2.29	1	0	138906.3906
High	Q9BLE6	Rab family GTPase OS=Entamoeba histolytica OX=5759 GN=EhRabF5 PE=2 SV=1	0	2.402	7	1	1	1	196	21.3	7.74	2.22	1	0	
High	A0A5K1UTW9	Thioredoxin domain-containing protein OS=Entamoeba histolytica OX=5759 GN=CL6EHI_175280 PE=4 SV=1	0	2.385	2	1	1	1	510	59.7	5.6	1.89	1	0	
High	A0A5K1UPB5	DNAj domain containing protein OS=Entamoeba histolytica OX=5759 GN=CL6EHI_134630 PE=4 SV=1	0	2.348	2	1	2	1	719	83.9	6.02	2.42	1	0	306410.6563
High	A0A5K1UZG0	Ribosomal protein s14 putative OS=Entamoeba histolytica	0	2.348	16	2	4	2	146	15.6	10.1	1.61	2	0	1527166.125

		OX=5759 GN=CL6EHI_012360 PE=3 SV=1													
High	A0A5K1UCX9	DNA topoisomerase OS=Entamoeba histolytica OX=5759 GN=CL6EHI_042880 PE=3 SV=1	0	2.345	2	1	1	1	840	95.2	8.47	0	1	0	
High	A0A175JIF7	Dynamin 1 like protein putative OS=Entamoeba histolytica OX=5759 GN=CL6EHI_013180 PE=4 SV=1	0	2.331	3	2	2	2	682	76.8	6.44	0	2	0	4734104.143
High	A0A5K1TVX7	Haloacid dehalogenase-like hydrolase OS=Entamoeba histolytica OX=5759 GN=CL6EHI_151930 PE=2 SV=1	0	2.309	6	2	3	2	303	33.7	5.34	3.45	2	0	3047622.031
High	A0A5K1U871	Rap ran GTPase-activating protein putative OS=Entamoeba histolytica OX=5759 GN=CL6EHI_074060 PE=4 SV=1	0	2.266	2	1	1	1	583	67.5	5.57	1.85	1	0	
High	A0A5K1U1H7	Uncharacterized protein OS=Entamoeba histolytica OX=5759 GN=CL6EHI_169970 PE=4 SV=1	0	2.249	3	1	1	1	368	42.8	5.68	2.4	1	0	256424.8281
High	ENSEMBL:ENSBTAP00000024146	(Bos taurus) similar to alpha-2-macroglobulin isoform 1	0	2.244	1	1	1	1	1477	164.2	6.02	2.4	1	0	582088.8125
High	A0A5K1UC77	Right handed beta helix domain-containing protein OS=Entamoeba histolytica	0	2.232	3	1	1	1	448	47.5	5.5	0	1	0	

		OX=5759 GN=CL6EHI_103420 PE=4 SV=1													
High	A0A5K1UQV4	mRNA decapping protein putative OS=Entamoeba histolytica OX=5759 GN=CL6EHI_058810 PE=3 SV=1	0	2.217	5	1	1	1	232	27.5	7.09	2.53	1	0	
High	A0A5K1V156	mannose-6-phosphate isomerase OS=Entamoeba histolytica OX=5759 GN=CL6EHI_162210 PE=3 SV=1	0	2.215	3	1	1	1	372	42.8	6.84	2.21	1	0	107560.4219
High	A0A5K1UCD7	non-specific serine/threonine protein kinase OS=Entamoeba histolytica OX=5759 GN=CL6EHI_104670 PE=4 SV=1	0	2.21	3	1	1	1	386	44.6	7.75	0	1	0	
High	A0A5K1U0Q0	Serine/threonine-protein phosphatase OS=Entamoeba histolytica OX=5759 GN=CL6EHI_069970 PE=3 SV=1	0	2.206	3	2	3	2	532	60.8	5.53	3.93	2	0	221789.1719
High	A0A5K1URN4	sphinganine-1-phosphate aldolase OS=Entamoeba histolytica OX=5759 GN=CL6EHI_039350 PE=3 SV=1	0	2.206	2	1	1	1	514	57.6	6.48	2.03	1	0	
High	A0A5K1VK47	AP complex subunit beta OS=Entamoeba histolytica OX=5759 GN=CL6EHI_023600 PE=3 SV=1	0	2.197	3	2	3	2	724	80.9	5.24	0	2	0	117393.1719
High	A0A5K1U5Q4	NAD FAD-dependent dehydrogenase putative OS=Entamoeba histolytica OX=5759 GN=CL6EHI_099700 PE=4 SV=1	0	2.181	1	1	2	1	1066	116.1	6.95	0	1	0	262506.8125

High	A0A5K1U4F2	Eukaryotic translation initiation factor 2 alpha subunit putative OS=Entamoeba histolytica OX=5759 GN=CL6EHI_005100 PE=3 SV=1	0	2.171	5	1	1	1	299	33.5	5.17	2.16	1	0	
High	A0A5K1VB03	AP 1 complex subunit mu 2 putative OS=Entamoeba histolytica OX=5759 GN=CL6EHI_124460 PE=3 SV=1	0	2.164	3	1	1	1	427	49.7	8.15	2.78	1	0	
High	A0A5K1UCX1	WD domain containing protein OS=Entamoeba histolytica OX=5759 GN=CL6EHI_118780 PE=3 SV=1	0	2.16	6	2	2	2	341	37.5	7.53	2.18	2	0	570967.9219
High	REFSEQ:XP_986630	Tax_Id=10090 Gene_Symbol=Krt33b keratin complex 1, acidic, gene 3	0	2.159	3	2	8	1	476	53.7	5.29	6.53	2	0	
High	P02754	SWISS-PROT:P02754 Beta-lactoglobulin - Bos taurus (Bovine).	0	2.145	9	2	3	2	162	18.3	4.92	1.85	2	0	369014.1563
High	A0A5K1UGR4	Short chain dehydrogenase family protein OS=Entamoeba histolytica OX=5759 GN=CL6EHI_164760 PE=4 SV=1	0	2.142	3	1	2	1	298	33.2	7.34	3.93	1	0	825109.4375
High	A0A5K1UY18	ATP-binding cassette protein putative OS=Entamoeba histolytica OX=5759 GN=CL6EHI_047760 PE=4 SV=1	0	2.103	2	1	1	1	630	71.4	7.06	2.17	1	0	161356.5469
High	A0A5K1VHH3	60S acidic ribosomal protein P0 OS=Entamoeba histolytica OX=5759 GN=CL6EHI_090400 PE=2 SV=1	0	2.088	8	2	2	2	316	34.3	6.09	0	2	0	262019.3594

High	A0A175JJT3	Uncharacterized protein OS=Entamoeba histolytica OX=5759 GN=CL6EHI_004580 PE=4 SV=1	0	2.083	6	1	1	1	172	19.9	9.38	1.66	1	0	317099.625
High	A0A5K1V2X9	Vesicle-fusing ATPase OS=Entamoeba histolytica OX=5759 GN=CL6EHI_004640 PE=3 SV=1	0	2.077	1	1	1	1	736	82.6	6.46	0	1	0	181720.3438
High	Q5NT21	Rab family GTPase (Fragment) OS=Entamoeba histolytica OX=5759 GN=EhRabK2 PE=3 SV=1	0	2.066	6	1	1	1	209	24.1	5.03	0	1	0	
High	A0A5K1VNZ7	isoleucine--tRNA ligase OS=Entamoeba histolytica OX=5759 GN=CL6EHI_177660 PE=3 SV=1	0	2.063	2	2	2	2	1056	122.7	7.01	0	2	0	
High	A0A175K0U6	Leucine rich repeat protein bspa family OS=Entamoeba histolytica OX=5759 GN=CL6EHI_122410 PE=4 SV=1	0	2.048	2	1	1	1	635	71.9	5.19	2.28	1	0	
High	A0A5K1VCM6	Phosphoglucomutase phosphomannomutase family protei OS=Entamoeba histolytica OX=5759 GN=CL6EHI_084950 PE=3 SV=1	0	2.047	3	2	2	2	454	49.7	7.01	0	2	0	102116.8281
High	A0A5K1U3D8	Peptidyl-prolyl cis-trans isomerase OS=Entamoeba histolytica OX=5759 GN=CL6EHI_044730 PE=3 SV=1	0	2.031	12	1	1	1	120	13.2	9.5	0	1	0	
High	A0A5K1UJ07	Uncharacterized protein OS=Entamoeba histolytica	0	2.025	3	1	1	1	298	34.7	6.95	0	1	0	490578.2188

		OX=5759 GN=CL6EHI_057690 PE=4 SV=1													
High	A0A5K1UMD3	Replication factor c familiy protein OS=Entamoeba histolytica OX=5759 GN=CL6EHI_099850 PE=3 SV=1	0	2.006	3	1	1	1	325	36	6.77	2.32	1	0	
High	A0A5K1VJ78	Uncharacterized protein OS=Entamoeba histolytica OX=5759 GN=CL6EHI_020330 PE=4 SV=1	0	1.985	6	1	1	1	196	22.2	4.65	0	1	0	
High	Q6KB66-1	SWISS-PROT:Q6KB66-1 Tax_Id=9606 Gene_Symbol=KRT80 Isoform 1 of Keratin, type II cytoskeletal 80	0	1.984	2	1	1	1	452	50.5	5.67	2.42	1	0	164345.8125
High	A0A5K1UTK0	Long-chain-fatty-acid--coa ligase putative OS=Entamoeba histolytica OX=5759 GN=CL6EHI_025090 PE=3 SV=1	0	1.972	2	1	1	1	683	78.6	7.02	2.63	1	0	231272.1875
High	A0A5K1VGH2	LIM Zinc finger domain containing protein OS=Entamoeba histolytica OX=5759 GN=CL6EHI_110280 PE=2 SV=1	0	1.969	4	1	1	1	301	32.8	8.37	2.44	1	0	192036.0156
High	A0A5K1V1K8	Arrestin C-terminal-like domain-containing protein OS=Entamoeba histolytica OX=5759 GN=CL6EHI_167660 PE=4 SV=1	0	1.963	2	1	1	1	649	74.2	7.5	1.86	1	0	
High	A0A5K1U2Q1	Leucine-rich repeat containing protein OS=Entamoeba	0	1.939	1	1	1	1	883	99	8.41	1.67	1	0	

		histolytica OX=5759 GN=CL6EHI_177990 PE=4 SV=1													
High	A0A5K 1U3A1	Protein kinase putative OS=Entamoeba histolytica OX=5759 GN=CL6EHI_158590 PE=4 SV=1	0	1.932	1	1	1	1	675	77	8.88	0	1	0	
High	A0A5K 1USI8	Type a flavoprotein putative OS=Entamoeba histolytica OX=5759 GN=CL6EHI_159860 PE=4 SV=1	0	1.925	2	1	1	1	413	46.7	5.31	0	1	0	279249.4063
High	A0A5K 1UKK4	Small ribosomal subunit protein eS1 OS=Entamoeba histolytica OX=5759 GN=CL6EHI_050280 PE=3 SV=1	0	1.919	4	1	1	1	256	29.1	9.85	2.53	1	0	211663.0313
High	A0A17 5JWU8	p-glyco protein 6 putative OS=Entamoeba histolytica OX=5759 GN=CL6EHI_101230 PE=4 SV=1	0	1.908	1	1	1	1	128 2	142. 4	7.11	0	1	0	
High	A0A17 5JXR7	Coronin putative OS=Entamoeba histolytica OX=5759 GN=CL6EHI_083590A PE=4 SV=1	0	1.889	8	1	1	1	123	13.8	5.01	1.86	1	0	325185.7188
High	A0A5K 1ULL0	DUF676 domain-containing protein OS=Entamoeba histolytica OX=5759 GN=CL6EHI_177530 PE=4 SV=1	0	1.877	3	1	1	1	401	46.5	5.81	2.2	1	0	
High	A0A5K 1VQI6	TLDc domain-containing protein OS=Entamoeba histolytica OX=5759 GN=CL6EHI_164770 PE=4 SV=1	0	1.869	6	2	2	2	260	29.9	8.66	1.63	2	0	280675.5625
High	A0A5K 1TVP5	Vacuolar sorting protein 26 putative OS=Entamoeba	0	1.863	4	1	1	1	377	43.6	5.49	0	1	0	

		histolytica OX=5759 GN=CL6EHI_147450 PE=3 SV=1												
High	A0A5K1U8U4	Brix domain containing protein OS=Entamoeba histolytica OX=5759 GN=CL6EHI_012190 PE=4 SV=1	0	1.859	5	1	1	1	353	41.3	8.56	0	1	0
High	A0A5K1V4Y2	GOLD domain-containing protein OS=Entamoeba histolytica OX=5759 GN=CL6EHI_138080 PE=4 SV=1	0	1.859	5	1	1	1	203	23.4	5.88	2.49	1	0	928731.5625
High	A0A175JKK4	Papain family cysteine protease domain containing protein OS=Entamoeba histolytica OX=5759 GN=CL6EHI_010340 PE=4 SV=1	0	1.81	2	1	1	1	557	64.3	4.96	0	1	0	274702.9063
High	A0A5K1U5Q0	Leucine-rich repeat containing protein OS=Entamoeba histolytica OX=5759 GN=CL6EHI_119950 PE=2 SV=1	0	1.804	4	1	1	1	351	40.5	6.79	0	1	0	
High	P01966	SWISS-PROT:P01966 (Bos taurus) Hemoglobin subunit alpha	0	1.789	6	1	1	1	142	15.2	8.44	0	1	0	277069.2813
High	A0A5K1U7G7	Obg-like ATPase 1 OS=Entamoeba histolytica OX=5759 GN=CL6EHI_148270 PE=3 SV=1	0	1.786	2	1	1	1	396	44.3	7.37	0	1	0	
High	A0A5K1U613	Metallo-beta-lactamase family protein OS=Entamoeba histolytica OX=5759 GN=CL6EHI_169860 PE=4 SV=1	0	1.781	4	1	2	1	261	29.5	5.94	0	1	0	410660.6875
High	A0A175JEY7	60S ribosomal protein L14 putative OS=Entamoeba	0	1.77	7	1	1	1	135	15.1	11.06	0	1	0	

		histolytica OX=5759 GN=CL6EHI_050130 PE=3 SV=1												
High	A0A5K1UPU9	RNA-binding protein putative OS=Entamoeba histolytica OX=5759 GN=CL6EHI_053170 PE=4 SV=1	0	1.769	3	1	2	1	337	36.9	4.69	0	1	0	318071.8438
High	A0A5K1UFD6	Nucleosome assembly protein putative OS=Entamoeba histolytica OX=5759 GN=CL6EHI_119340 PE=3 SV=1	0	1.729	3	1	1	1	332	38.1	4.5	0	1	0	
High	A0A5K1UT97	26S proteasome non-ATPase regulatory subunit 5 OS=Entamoeba histolytica OX=5759 GN=CL6EHI_041980 PE=4 SV=1	0	1.722	1	1	2	1	753	84.2	5.54	0	1	0	65349.41406
High	A0A5K1UEL4	Serine/threonine-protein phosphatase OS=Entamoeba histolytica OX=5759 GN=CL6EHI_199120 PE=3 SV=1	0	1.714	2	1	2	1	525	61.3	6.86	4.31	1	0	1636406
High	A0A5K1TWR5	Rab gdp dissociation inhibitor putative OS=Entamoeba histolytica OX=5759 GN=CL6EHI_074100 PE=3 SV=1	0	1.703	2	1	1	1	480	55.1	5.44	0	1	0	176702.0625
High	A0A5K1TZR0	Proteasome subunit alpha type OS=Entamoeba histolytica OX=5759 GN=CL6EHI_098060 PE=3 SV=1	0	1.699	4	1	2	1	247	27.7	7.37	2.16	1	0	1113161.125
High	A0A5K1U6C9	Leucine-rich repeat containing protein OS=Entamoeba histolytica OX=5759 GN=CL6EHI_056090 PE=4 SV=1	0	1.696	2	1	2	1	578	66.8	6.4	0	1	0	103006.6719

High	A0A5K1UY58	WD domain containing protein OS=Entamoeba histolytica OX=5759 GN=CL6EHI_103620 PE=3 SV=1	0	1.692	3	1	1	1	392	45	6.3	2.31	1	0	
High	A0A175JJI4	MRH domain-containing protein OS=Entamoeba histolytica OX=5759 GN=CL6EHI_087320 PE=4 SV=1	0	1.682	4	1	2	1	230	26.7	6.73	0	1	0	
High	A0A5K1U1D8	Signal peptidase complex subunit 3 OS=Entamoeba histolytica OX=5759 GN=CL6EHI_121860 PE=3 SV=1	0	1.678	5	1	1	1	168	19.5	8.29	0	1	0	
High	A0A5K1VLJ6	Ikappab kinase complex-associated protein OS=Entamoeba histolytica OX=5759 GN=CL6EHI_151220 PE=3 SV=1	0	1.675	2	2	2	2	759	87.3	6.09	0	2	0	382290.1563
High	A0A5K1V4Q0	small monomeric GTPase OS=Entamoeba histolytica OX=5759 GN=CL6EHI_012240 PE=3 SV=1	0	1.661	5	1	2	1	200	22.3	6.05	1.6	1	0	571023.25
High	A0A5K1VFN7	Dead deah box helicase putative OS=Entamoeba histolytica OX=5759 GN=CL6EHI_069410 PE=4 SV=1	0	1.655	2	1	2	1	684	78.6	9.32	0	1	0	
High	A0A5K1VRT3	Ribulose-phosphate 3-epimerase OS=Entamoeba histolytica OX=5759 GN=CL6EHI_006900 PE=3 SV=1	0	1.647	5	1	1	1	216	23.7	6.34	2.31	1	0	621383.9375
High	A0A5K1V5U2	Phosphatidylserine synthase putative OS=Entamoeba	0	1.646	5	1	1	1	442	52.7	7.21	0	1	0	65430.61719

		histolytica OX=5759 GN=CL6EHI_009800 PE=4 SV=1													
High	A0A175JWR2	Protein phosphatase domain-containing protein OS=Entamoeba histolytica OX=5759 GN=CL6EHI_075640 PE=4 SV=1	0	1.644	1	1	1	1	769	86.6	5.12	0	1	0	
High	Q5NSY1	Rab family GTPase (Fragment) OS=Entamoeba histolytica OX=5759 GN=EhRabX35 PE=3 SV=1	0	1.643	6	1	2	1	194	21.9	5	0	1	0	171840.8125
High	A0A5K1V2C1	Vacuolar protein sorting-associated protein 35 putative OS=Entamoeba histolytica OX=5759 GN=CL6EHI_041950 PE=3 SV=1	0	1.642	1	1	1	1	746	85.1	5.53	2.11	1	0	
High	A0A5K1UZV1	Protein kinase putative OS=Entamoeba histolytica OX=5759 GN=CL6EHI_011510 PE=4 SV=1	0	1.632	2	1	2	1	424	48.2	6.04	0	1	0	282816.8438
High	A0A5K1U7Z3	Uncharacterized protein OS=Entamoeba histolytica OX=5759 GN=CL6EHI_140680 PE=4 SV=1	0	1.629	2	1	1	1	650	75.1	5.54	1.84	1	0	
High	A0A175JYU1	Serine threonine-protein phosphatase 2a 65 kDa regulatory subunit A putative OS=Entamoeba histolytica OX=5759 GN=CL6EHI_019620 PE=4 SV=1	0	1.628	2	1	1	1	618	70.8	5.63	0	1	0	118909.7344
High	A0A5K1UEQ0	holo-[acyl-carrier-protein] synthase OS=Entamoeba	0	1.607	3	1	1	1	259	30.3	6.76	1.64	1	0	

		histolytica OX=5759 GN=CL6EHI_168270 PE=4 SV=1													
High	A0A5K 1U4Z2	60S ribosomal protein L31 putative OS=Entamoeba histolytica OX=5759 GN=CL6EHI_068200 PE=2 SV=1	0	1.606	11	2	2	2	150	17.2	10.3 6	0	2	0	
High	A0A5K 1VML6	peptidylprolyl isomerase OS=Entamoeba histolytica OX=5759 GN=CL6EHI_178850 PE=4 SV=1	0	1.58	3	1	1	1	394	45.7	5.12	0	1	0	
High	A0A5K 1VJL5	Signal recognition particle subunit SRP68 OS=Entamoeba histolytica OX=5759 GN=CL6EHI_146790 PE=3 SV=1	0	1.58	2	1	2	1	457	54.5	8.76	0	1	0	149354.4844
High	A0A5K 1TXG0	Nucleolar complex protein 2 homolog putative OS=Entamoeba histolytica OX=5759 GN=CL6EHI_105790 PE=3 SV=1	0	1.559	3	1	1	1	508	58.3	5.69	0	1	0	
High	A0A5K 1VDR2	Ras-related protein Rab OS=Entamoeba histolytica OX=5759 GN=CL6EHI_177520 PE=3 SV=1	0	1.554	5	1	1	1	209	23.4	5.25	0	1	0	
High	Q5NT1 6	Rab family GTPase (Fragment) OS=Entamoeba histolytica OX=5759 GN=EhRabM2 PE=2 SV=1	0	1.541	4	1	1	1	203	22.9	5.19	0	1	0	
High	A0A5K 1VSZ3	non-specific serine/threonine protein kinase OS=Entamoeba histolytica OX=5759 GN=CL6EHI_127410 PE=4 SV=1	0	1.521	2	1	1	1	519	58.7	6.73	2.05	1	0	

High	Q5NT31	Rab family GTPase (Fragment) OS=Entamoeba histolytica OX=5759 GN=EhRabI2 PE=3 SV=1	0	1.511	5	1	2	1	213	23.6	5	0	1	0	199056.9375
High	A0A5K1VSP4	Sel1 repeat-containing protein OS=Entamoeba histolytica OX=5759 GN=CL6EHI_187760 PE=3 SV=1	0	1.51	3	1	1	1	241	26.4	8.53	0	1	0	18809.91016
High	A0A5K1V8R8	Alanine--tRNA ligase OS=Entamoeba histolytica OX=5759 GN=CL6EHI_198790 PE=3 SV=1	0	1.498	2	2	2	2	980	111	7.12	0	2	0	
High	A0A5K1VHX2	Protein SCAI OS=Entamoeba histolytica OX=5759 GN=CL6EHI_186790 PE=4 SV=1	0	1.493	1	1	2	1	559	65.1	8.21	0	1	0	
High	A0A5K1UU94	EF-hand calcium-binding domain containing protein OS=Entamoeba histolytica OX=5759 GN=CL6EHI_168390 PE=4 SV=1	0	1.487	3	1	2	1	317	37	6.68	0	1	0	341556.2188
High	A0A5K1VF08	CCT-beta OS=Entamoeba histolytica OX=5759 GN=CL6EHI_103270 PE=3 SV=1	0	1.487	2	1	1	1	533	58.2	6.05	1.84	1	0	742653.9375
High	A0A5K1TYT7	tRNA pseudouridine synthase d domain-containing protein OS=Entamoeba histolytica OX=5759 GN=CL6EHI_199620 PE=3 SV=1	0	1.479	2	1	1	1	634	73	8.41	0	1	0	172076.7656
High	A0A175JMX9	Rab GTPase activating protein putative OS=Entamoeba histolytica OX=5759 GN=CL6EHI_015260 PE=4 SV=1	0	1.46	3	1	1	1	320	37	6	1.83	1	0	

High	A0A5K1U0E7	60S ribosomal protein L4 putative OS=Entamoeba histolytica OX=5759 GN=CL6EHI_194270 PE=3 SV=1	0	1.46	4	1	1	1	431	47.3	10.83	0	1	0	
High	A0A5K1UCJ4	Protein kinase putative OS=Entamoeba histolytica OX=5759 GN=CL6EHI_137980 PE=3 SV=1	0	1.453	3	1	2	1	272	30.4	4.68	1.73	1	0	502621.8125
High	A0A5K1UZ22	EF-hand calcium-binding domain containing protein OS=Entamoeba histolytica OX=5759 GN=CL6EHI_197510 PE=4 SV=1	0	1.451	3	1	2	1	264	29.9	6.15	3.33	1	0	385267.1875
High	A0A5K1VL37	T-complex protein 1 subunit delta OS=Entamoeba histolytica OX=5759 GN=CL6EHI_114120 PE=3 SV=1	0	1.448	1	1	3	1	538	58.2	6.48	0	1	0	872943.3125
High	A0A5K1UCT0	Uncharacterized protein OS=Entamoeba histolytica OX=5759 GN=CL6EHI_114350 PE=3 SV=1	0.002	1.416	2	1	1	1	627	72.5	6.44	1.6	1	0	321537.0313
High	A0A5K1VQT0	phenylalanine--tRNA ligase OS=Entamoeba histolytica OX=5759 GN=CL6EHI_002130 PE=3 SV=1	0.002	1.409	1	1	1	1	574	64.8	5.54	0	1	0	3522684
High	A0A5K1UM28	CAAX prenyl protease OS=Entamoeba histolytica OX=5759 GN=CL6EHI_075660 PE=3 SV=1	0.002	1.406	2	1	1	1	416	48.6	6.54	1.94	1	0	
High	A0A5K1TYK9	GTPase-activator protein for Ras family GTPase OS=Entamoeba	0.002	1.393	2	1	3	1	393	45.5	5.16	0	1	0	188327.7969

		histolytica OX=5759 GN=CL6EHI_017630 PE=4 SV=1													
High	A0A5K1UGL2	FG-GAP repeat-containing protein OS=Entamoeba histolytica OX=5759 GN=CL6EHI_007970 PE=4 SV=1	0.002	1.385	2	1	1	1	623	72.2	5.45	2.09	1	0	351069.6875
High	A0A5K1U7Q4	Long-chain-fatty-acid--coa ligase putative OS=Entamoeba histolytica OX=5759 GN=CL6EHI_148340 PE=3 SV=1	0.002	1.377	3	1	1	1	642	73.2	6.2	0	1	0	
High	A0A5K1VJY2	sphingomyelin phosphodiesterase OS=Entamoeba histolytica OX=5759 GN=CL6EHI_125790 PE=3 SV=1	0.002	1.372	3	1	2	1	320	37	6.55	0	1	0	157471.2813
High	A0A5K1UCR2	40S ribosomal protein S6 OS=Entamoeba histolytica OX=5759 GN=CL6EHI_013890 PE=3 SV=1	0.002	1.369	4	1	3	1	266	29.7	10.48	3.61	1	0	709491
High	A0A5K1UQQ0	Kinase OS=Entamoeba histolytica OX=5759 GN=CL6EHI_051050 PE=3 SV=1	0.002	1.367	4	1	1	1	280	32.7	6.86	0	1	0	172959.0625
High	A0A5K1TYR7	Serine threonine protein kinase putative OS=Entamoeba histolytica OX=5759 GN=CL6EHI_162340 PE=4 SV=1	0.002	1.364	1	1	1	1	607	71.4	7.21	0	1	0	206065.5625
High	A0A5K1UC90	Ras guanine nucleotide exchange factor putative OS=Entamoeba histolytica OX=5759 GN=CL6EHI_023270 PE=4 SV=1	0.002	1.357	1	1	2	1	910	104	5.62	0	1	0	141844.1719

High	A0A175JSI0	Beta-1,4-mannosyltransferase OS=Entamoeba histolytica OX=5759 GN=CL6EHI_028950 PE=4 SV=1	0.002	1.352	3	1	3	1	456	53	7.65	0	1	0	
High	A0A5K1UZ10	Protein kinase putative OS=Entamoeba histolytica OX=5759 GN=CL6EHI_002790 PE=3 SV=1	0.002	1.331	2	1	2	1	474	54.9	7.15	0	1	0	441513.3438
High	A0A5K1U2R6	Importin beta putative OS=Entamoeba histolytica OX=5759 GN=CL6EHI_036520 PE=4 SV=1	0.002	1.328	2	1	1	1	843	94.5	5.34	0	1	0	
High	A0A5K1VTU0	Ribosomal protein L10 putative OS=Entamoeba histolytica OX=5759 GN=CL6EHI_044810 PE=2 SV=1	0.002	1.324	4	1	1	1	210	23.8	9.98	2.15	1	0	243572.4375
High	A0A5K1UM94	Ribosome biogenesis protein bms1 putative OS=Entamoeba histolytica OX=5759 GN=CL6EHI_196410 PE=4 SV=1	0.002	1.324	1	1	2	1	975	111.9	7.02	3.56	1	0	
High	Q5NT23	Rab family GTPase (Fragment) OS=Entamoeba histolytica OX=5759 GN=EhRabK1 PE=3 SV=1	0.002	1.323	4	1	3	1	212	24.5	5.03	0	1	0	
High	A0A5K1VFK2	40S ribosomal protein s5 OS=Entamoeba histolytica OX=5759 GN=CL6EHI_044590 PE=3 SV=1	0.002	1.32	5	1	3	1	206	23	9.51	4.34	1	0	485341.8438
High	A0A5K1VA36	Uncharacterized protein OS=Entamoeba histolytica OX=5759 GN=CL6EHI_035510 PE=2 SV=1	0.002	1.319	4	1	1	1	262	28.6	5.12	0	1	0	151101.4688

High	A0A5K1ULM5	Filamin 2 putative OS=Entamoeba histolytica OX=5759 GN=CL6EHI_104630 PE=4 SV=1	0.002	1.315	1	1	1	1	857	95.3	5.06	1.76	1	0	142115.9063
High	A0A5K1U5P8	small monomeric GTPase OS=Entamoeba histolytica OX=5759 GN=CL6EHI_070730 PE=4 SV=1	0.002	1.313	5	1	2	1	194	21.6	8.62	1.78	1	0	290207.9375
High	A0A5K1UWB9	Proteasome regulatory subunit putative OS=Entamoeba histolytica OX=5759 GN=CL6EHI_136180 PE=3 SV=1	0.002	1.301	2	1	1	1	448	52.2	5.81	0	1	0	
High	A0A5K1UWV7	Phosphoinositide phosphatase putative OS=Entamoeba histolytica OX=5759 GN=CL6EHI_141860 PE=4 SV=1	0.002	1.29	1	1	1	1	586	67.8	7.72	0	1	0	196850.2031
High	A0A5K1VCU1	TLDc domain-containing protein OS=Entamoeba histolytica OX=5759 GN=CL6EHI_036570 PE=4 SV=1	0.002	1.286	2	1	5	1	383	43.8	6.61	7.01	1	0	328405.9375
High	A0A5K1VP75	Alkyl sulfatase putative OS=Entamoeba histolytica OX=5759 GN=CL6EHI_086530 PE=3 SV=1	0.002	1.284	2	1	1	1	628	70.7	6.62	0	1	0	
High	A0A5K1UB02	Ras guanine nucleotide exchange factor putative OS=Entamoeba histolytica OX=5759 GN=CL6EHI_113430 PE=4 SV=1	0.002	1.262	3	1	2	1	440	51.1	5.03	0	1	0	
High	A0A5K1UR72	Serine/threonine-protein phosphatase 2A 55 kDa regulatory subunit B	0.002	1.259	2	1	2	1	440	50.4	5.71	0	1	0	368736.75

		OS=Entamoeba histolytica OX=5759 GN=CL6EHI_148170 PE=3 SV=1													
High	A0A5K1TWX1	Rasgef domain containing protein OS=Entamoeba histolytica OX=5759 GN=CL6EHI_072110 PE=4 SV=1	0.002	1.258	1	1	1	1	894	102.9	8.05	1.7	1	0	126153.6406
High	A0A5K1VEC7	Protein tyrosine kinase domain-containing protein OS=Entamoeba histolytica OX=5759 GN=CL6EHI_197350 PE=4 SV=1	0.002	1.257	1	1	1	1	1055	121.2	5.96	1.96	1	0	
High	A0A5K1UN03	Serine threonine protein phosphatase type 5 putative OS=Entamoeba histolytica OX=5759 GN=CL6EHI_126930 PE=4 SV=1	0.002	1.252	2	1	1	1	388	43.6	7.56	1.89	1	0	
High	A0A175JKL9	Uncharacterized protein OS=Entamoeba histolytica OX=5759 GN=CL6EHI_138000 PE=4 SV=1	0.002	1.251	2	1	1	1	514	60	5.76	0	1	0	
High	A0A5K1V8V9	Protein kinase domain containing protein OS=Entamoeba histolytica OX=5759 GN=CL6EHI_186820 PE=3 SV=1	0.002	1.243	2	1	1	1	419	47.4	6.62	1.64	1	0	122634.4531
High	A0A5K1VA33	Exosome complex exonuclease putative OS=Entamoeba histolytica OX=5759 GN=CL6EHI_000580 PE=3 SV=1	0.002	1.243	4	1	2	1	265	29.3	7.83	0	1	0	230885.7188
High	A0A175K1R9	Nadph-dependent fmn reductase domain containing	0.002	1.242	4	1	2	1	212	24	6.52	3.56	1	0	431910.2813

		protein OS=Entamoeba histolytica OX=5759 GN=CL6EHI_181710 PE=4 SV=1													
High	A0A5K1U5A9	Piwi putative OS=Entamoeba histolytica OX=5759 GN=CL6EHI_125650 PE=3 SV=1	0.002	1.239	2	1	1	1	937	109.6	8.53	0	1	0	
High	A0A5K1UD89	Multidrug resistance protein putative OS=Entamoeba histolytica OX=5759 GN=CL6EHI_100320 PE=3 SV=1	0.002	1.228	1	1	1	1	1600	181.4	7.27	0	1	0	
High	A0A5K1UUN1	glutamine--tRNA ligase OS=Entamoeba histolytica OX=5759 GN=CL6EHI_062700 PE=3 SV=1	0.002	1.223	1	1	1	1	753	86.5	7.87	0	1	0	
High	A0A175JF09	Protein kinase putative OS=Entamoeba histolytica OX=5759 GN=CL6EHI_068350 PE=4 SV=1	0.002	1.222	1	1	1	1	798	93.2	6.39	0	1	0	367492.0313
High	A0A5K1U0W2	Peptidase putative OS=Entamoeba histolytica OX=5759 GN=CL6EHI_175020 PE=3 SV=1	0.002	1.221	2	1	3	1	372	41.6	5.6	1.64	1	0	394782.1563
High	A0A5K1U1D5	Actin-related protein 2/3 complex subunit 4 OS=Entamoeba histolytica OX=5759 GN=CL6EHI_030820 PE=3 SV=1	0.002	1.211	4	1	2	1	191	21.5	8.57	0	1	0	327720.8438
High	A0A5K1VFQ4	aspartyl aminopeptidase OS=Entamoeba histolytica OX=5759 GN=CL6EHI_021340 PE=3 SV=1	0.002	1.21	2	1	1	1	435	48.7	5.39	0	1	0	

High	A0A5K1VBF2	WD domain containing protein OS=Entamoeba histolytica OX=5759 GN=CL6EHI_103410 PE=3 SV=1	0.002	1.208	1	1	1	1	998	111.4	5.49	0	1	0	
High	A0A5K1UVH5	Vacuolar proton pump subunit B OS=Entamoeba histolytica OX=5759 GN=CL6EHI_189850 PE=3 SV=1	0.001	1.195	2	1	1	1	496	55.3	5.11	1.74	1	0	469756.75
High	A0A175JTX6	Rab family GTPase OS=Entamoeba histolytica OX=5759 GN=CL6EHI_012030 PE=3 SV=1	0.001	1.191	6	1	1	1	194	21.8	6.79	0	1	0	295086.6875
High	P02663	SWISS-PROT:P02663 Alpha-S2-casein [Contains: Casocidin-1 - Bos taurus (Bovine).	0.001	1.191	4	1	1	1	207	24.3	8.27	2.24	1	0	
High	A0A5K1TYC3	Glycerophosphoryl diester phosphodiesterase putative OS=Entamoeba histolytica OX=5759 GN=CL6EHI_113970 PE=4 SV=1	0.001	1.186	2	1	1	1	380	43.5	8.4	1.8	1	0	
High	A0A5K1VV20	Mitogen-activated protein kinase OS=Entamoeba histolytica OX=5759 GN=CL6EHI_073650 PE=3 SV=1	0.001	1.185	2	1	1	1	352	40	5.2	2.35	1	0	455691.7813
High	A0A5K1VKF0	3-ketoacyl-CoA synthase OS=Entamoeba histolytica OX=5759 GN=CL6EHI_158240 PE=3 SV=1	0.001	1.179	2	1	1	1	495	56.5	8.46	1.85	1	0	
High	Q1EQ48	EhSec24A OS=Entamoeba histolytica OX=5759 GN=CL6EHI_042990 PE=3 SV=1	0.001	1.178	1	1	1	1	618	71.2	7.91	0	1	0	

High	A0A5K1UMI9	Cullin family protein OS=Entamoeba histolytica OX=5759 GN=CL6EHI_118180 PE=3 SV=1	0.001	1.164	3	1	1	1	741	86.9	8.05	0	1	0	
High	A0A5K1ULR2	Nucleosome assembly protein putative OS=Entamoeba histolytica OX=5759 GN=CL6EHI_110450 PE=3 SV=1	0.001	1.154	3	1	2	1	243	28.6	5.34	0	1	0	177976.5313
High	A0A5K1V2B9	60S ribosomal protein L35a putative OS=Entamoeba histolytica OX=5759 GN=CL6EHI_014110 PE=3 SV=1	0.001	1.152	11	1	1	1	108	12.1	10.59	2.12	1	0	60010.35547
High	A0A5K1VQV5	Adp-ribosylglycohydrolase putative OS=Entamoeba histolytica OX=5759 GN=CL6EHI_198770 PE=4 SV=1	0.001	1.15	2	1	1	1	432	49.8	5.27	0	1	0	
High	Q9NJW1	Grainin 2 OS=Entamoeba histolytica OX=5759 GN=grainin 2 PE=2 SV=1	0.001	1.139	3	1	5	1	213	24	8.22	0	1	0	2601663.5
High	A0A5K1V4Y5	Protein farnesyltransferase subunit beta OS=Entamoeba histolytica OX=5759 GN=CL6EHI_006190 PE=3 SV=1	0.001	1.135	3	1	1	1	375	42.6	6.06	1.9	1	0	
High	A0A5K1TUA0	Large ribosomal subunit protein uL2 OS=Entamoeba histolytica OX=5759 GN=CL6EHI_127200 PE=3 SV=1	0.001	1.135	4	1	1	1	257	27.8	10.7	0	1	0	
High	A0A5K1UQR4	Rab3-GAP regulatory subunit N-terminal domain-containing protein OS=Entamoeba histolytica OX=5759 GN=CL6EHI_138010 PE=4 SV=1	0.001	1.132	3	1	1	1	417	47.9	6.7	0	1	0	

High	A0A5K1UEV6	START domain-containing protein OS=Entamoeba histolytica OX=5759 GN=CL6EHI_161070 PE=4 SV=1	0.001	1.126	5	1	1	1	211	23.8	6.24	0	1	0	
High	A0A5K1UZU1	Chorein putative OS=Entamoeba histolytica OX=5759 GN=CL6EHI_067970 PE=3 SV=1	0.001	1.126	0	1	1	1	3346	381.7	6.67	0	1	0	
High	A0A5K1U8I6	non-specific serine/threonine protein kinase OS=Entamoeba histolytica OX=5759 GN=CL6EHI_151950 PE=3 SV=1	0.001	1.125	3	1	1	1	335	38.7	8.22	0	1	0	
High	A0A5K1UCI4	glutamate--tRNA ligase OS=Entamoeba histolytica OX=5759 GN=CL6EHI_155570 PE=3 SV=1	0.001	1.123	1	1	3	1	551	62.8	8.31	0	1	0	6153910
High	A0A5K1U2Q8	Serine threonine protein kinase putative OS=Entamoeba histolytica OX=5759 GN=CL6EHI_008410 PE=4 SV=1	0.003	1.114	2	1	1	1	547	62.3	8.09	2.15	1	0	
High	A0A5K1V2N1	Coatomer subunit gamma OS=Entamoeba histolytica OX=5759 GN=CL6EHI_040700 PE=3 SV=1	0.004	1.109	1	1	2	1	844	95.3	5.58	3.94	1	0	188974.8594
High	A0A5K1U225	Protein kinase putative OS=Entamoeba histolytica OX=5759 GN=CL6EHI_092530 PE=3 SV=1	0.004	1.102	2	1	1	1	397	45.6	7.43	0	1	0	229050.6094
High	A0A175JVS8	Rho GTPase activating protein putative OS=Entamoeba histolytica OX=5759 GN=CL6EHI_035940 PE=4 SV=1	0.004	1.099	1	1	2	1	549	63.1	6.86	0	1	0	

High	A0A5K 1UYB7	Uncharacterized protein OS=Entamoeba histolytica OX=5759 GN=CL6EHI_000240 PE=3 SV=1	0.004	1.098	3	1	2	1	351	38.9	5.91	0	1	0	
High	A0A5K 1UJ55	Proliferating cell nuclear antigen OS=Entamoeba histolytica OX=5759 GN=CL6EHI_128450 PE=3 SV=1	0.004	1.098	3	1	1	1	262	28.5	4.7	0	1	0	
High	A0A5K 1U9V6	Guanine deaminase OS=Entamoeba histolytica OX=5759 GN=CL6EHI_151430 PE=3 SV=1	0.004	1.095	2	1	1	1	431	48.4	5.94	0	1	0	370666.375
High	A0A5K 1UHI3	Uncharacterized protein OS=Entamoeba histolytica OX=5759 GN=CL6EHI_112850 PE=4 SV=1	0.004	1.091	3	1	1	1	396	45.1	7.44	0	1	0	
High	A0A5K 1UXZ9	Translation initiation factor eIF2B subunit epsilon OS=Entamoeba histolytica OX=5759 GN=CL6EHI_131020 PE=3 SV=1	0.004	1.086	1	1	1	1	634	72.9	4.77	0	1	0	
High	A0A5K 1UQZ3	Serine threonine protein kinase putative OS=Entamoeba histolytica OX=5759 GN=CL6EHI_052800 PE=4 SV=1	0.004	1.084	1	1	1	1	907	103.3	9.01	0	1	0	1214195.75
High	A0A5K 1VPX1	Elongator complex protein 2 OS=Entamoeba histolytica OX=5759 GN=CL6EHI_111020 PE=3 SV=1	0.004	1.081	1	1	1	1	722	80.6	5.97	0	1	0	
High	A0A5K 1VU46	Cytoplasmic tRNA 2-thiolation protein 2 OS=Entamoeba	0.004	1.078	3	1	2	1	272	32.4	6.67	0	1	0	518907.5938

		histolytica OX=5759 GN=CL6EHI_166980 PE=4 SV=1													
High	A0A5K 1U8E2	Nad synthetase putative OS=Entamoeba histolytica OX=5759 GN=CL6EHI_134830 PE=4 SV=1	0.004	1.042	3	1	1	1	306	34.3	6.04	0	1	0	135209.1406
High	A0A5K 1VS28	Long-chain-fatty-acid--coa ligase putative OS=Entamoeba histolytica OX=5759 GN=CL6EHI_185240 PE=4 SV=1	0.004	1.032	2	1	1	1	774	87.1	8.12	2.47	1	0	277170.9375
High	A0A5K 1UFA7	Tbc domain containing protein OS=Entamoeba histolytica OX=5759 GN=CL6EHI_009510 PE=4 SV=1	0.004	1.021	3	1	1	1	312	36.3	4.94	0	1	0	
High	A0A17 5JTM0	Uncharacterized protein OS=Entamoeba histolytica OX=5759 GN=CL6EHI_065230 PE=4 SV=1	0.004	1.019	1	1	1	1	996	115. 2	6.39	0	1	0	
High	A0A5K 1V1G1	Ras family GTPase OS=Entamoeba histolytica OX=5759 GN=CL6EHI_091090 PE=4 SV=1	0.004	1.019	4	1	1	1	201	22.7	5.71	1.62	1	0	201899.4219
High	A0A5K 1VFM2	Queuosine 5'-phosphate N-glycosylase/hydrolase OS=Entamoeba histolytica OX=5759 GN=CL6EHI_098190 PE=3 SV=1	0.004	1.018	3	1	3	1	305	35.9	5.49	0	1	0	
High	A0A5K 1TW16	Ribosome biogenesis protein putative OS=Entamoeba histolytica OX=5759 GN=CL6EHI_175090 PE=3 SV=1	0.004	1.016	3	1	1	1	291	33.9	8.19	0	1	0	87777.01563

High	A0A5K1V7J9	TLDc domain-containing protein OS=Entamoeba histolytica OX=5759 GN=CL6EHI_027750 PE=4 SV=1	0.004	1.011	3	1	1	1	260	29.9	8.43	0	1	0	297006.375
High	A0A5K1UJQ0	Heat repeat domain containing protein OS=Entamoeba histolytica OX=5759 GN=CL6EHI_124870 PE=3 SV=1	0.004	0.995	2	1	1	1	549	61.9	5.1	0	1	0	
High	A0A5K1UQM3	AIG1 family protein putative OS=Entamoeba histolytica OX=5759 GN=CL6EHI_025990 PE=4 SV=1	0.004	0.992	2	1	3	1	298	34.8	4.98	0	1	0	218036.4688
High	Q2KJ83	TREMBL:Q2KJ83 (Bos taurus) Similar to Carboxypeptidase N catalytic chain	0.004	0.972	2	1	2	1	462	52.6	8.59	0	1	0	5832355.5
High	A0A5K1UNL0	Uncharacterized protein OS=Entamoeba histolytica OX=5759 GN=CL6EHI_035430 PE=4 SV=1	0.004	0.958	0	1	3	1	1801	208.1	5.85	0	1	0	
High	A0A5K1VT25	Mov34 mpn pad 1 family protein OS=Entamoeba histolytica OX=5759 GN=CL6EHI_050500 PE=3 SV=1	0.004	0.948	3	1	3	1	318	36.5	5.52	0	1	0	
High	A0A5K1UBQ7	AP-2 complex subunit alpha OS=Entamoeba histolytica OX=5759 GN=CL6EHI_045710 PE=3 SV=1	0.004	0.935	1	1	3	1	961	109.1	5.48	0	1	0	169133.6406
High	A0A5K1U5H9	Uncharacterized protein OS=Entamoeba histolytica OX=5759 GN=CL6EHI_125710 PE=4 SV=1	0.004	0.934	3	1	2	1	295	34.4	6.06	1.65	1	0	93535.42188

High	S0AVE7	Actin-related protein 3 OS=Entamoeba histolytica OX=5759 GN=CL6EHI_198930 PE=2 SV=1	0.004	0.93	3	1	1	1	416	46.9	5.52	2.06	1	0	
High	A0A5K1VK72	Uncharacterized protein OS=Entamoeba histolytica OX=5759 GN=CL6EHI_196400 PE=4 SV=1	0.004	0.916	1	1	2	1	597	68.2	6.74	2.06	1	0	1218798.75
High	A0A5K1UU82	Oxidoreductase short chain dehydrogenase reductase family OS=Entamoeba histolytica OX=5759 GN=CL6EHI_146380 PE=3 SV=1	0.005	0.904	3	1	1	1	272	31.1	5.58	0	1	0	5976476.5
High	A0A5K1TY28	Aldose 1-epimerase OS=Entamoeba histolytica OX=5759 GN=CL6EHI_094090 PE=3 SV=1	0.008	0.891	4	1	1	1	341	38.7	6.07	0	1	0	66334.33594
High	A0A5K1V0Q5	Heat shock protein 70 OS=Entamoeba histolytica OX=5759 GN=CL6EHI_131920 PE=3 SV=1	0.008	0.881	1	1	2	1	601	69.9	5.85	1.65	1	0	300182.3125
High	A0A5K1VU17	non-specific serine/threonine protein kinase OS=Entamoeba histolytica OX=5759 GN=CL6EHI_044640 PE=3 SV=1	0.009	0.873	2	1	1	1	384	43.8	7.14	0	1	0	
High	A0A5K1UKG1	Serine aminopeptidase S33 domain-containing protein OS=Entamoeba histolytica OX=5759 GN=CL6EHI_006710 PE=3 SV=1	0.009	0.871	2	1	1	1	447	51.1	6.52	1.78	1	0	144902.4531
Medium	A0A5K1V239	Rap ran GTPase-activating protein putative OS=Entamoeba	0.011	0.858	1	1	2	1	1340	154.1	6.1	1.69	1	0	142686.0313

		histolytica OX=5759 GN=CL6EHI_155710 PE=4 SV=1													
Medium	A0A5K1UQQ7	Gelsolin repeat protein putative OS=Entamoeba histolytica OX=5759 GN=CL6EHI_009570 PE=3 SV=1	0.011	0.85	0	1	1	1	1416	169	4.65	0	1	0	
Medium	A0A5K1UH78	Eukaryotic translation initiation factor 4e putative OS=Entamoeba histolytica OX=5759 GN=CL6EHI_040280 PE=3 SV=1	0.011	0.847	4	1	1	1	182	20.8	7.5	0	1	0	1127545.25
Medium	S0B0B9	Cysteine proteinase 1 putative OS=Entamoeba histolytica OX=5759 GN=CL6EHI_074180 PE=2 SV=1	0.011	0.843	2	1	8	1	315	35	6.05	0	1	0	
Medium	A0A5K1UAR0	Ras GTPase activating protein putative OS=Entamoeba histolytica OX=5759 GN=CL6EHI_035800 PE=4 SV=1	0.011	0.843	1	1	1	1	787	90.3	5.78	0	1	0	
Medium	A0A5K1U2W8	Fibronectin type-III domain-containing protein OS=Entamoeba histolytica OX=5759 GN=CL6EHI_020350 PE=2 SV=1	0.011	0.838	3	1	1	1	225	26.3	5.78	0	1	0	92402.14063
Medium	A0A5K1VV23	Uncharacterized protein OS=Entamoeba histolytica OX=5759 GN=CL6EHI_134980 PE=4 SV=1	0.012	0.837	1	1	2	1	846	97.3	8.34	1.61	1	0	184647.8281
Medium	A0A5K1VB93	Protein phosphatase putative OS=Entamoeba histolytica OX=5759 GN=CL6EHI_092510 PE=4 SV=1	0.012	0.833	3	1	1	1	263	29.6	5.43	0	1	0	

Medium	A0A5K1UTP9	PPM-type phosphatase domain-containing protein OS=Entamoeba histolytica OX=5759 GN=CL6EHI_037200 PE=4 SV=1	0.012	0.833	3	1	1	1	471	52.1	7.8	0	1	0	
Medium	Q6AW65	EhRab7D protein OS=Entamoeba histolytica OX=5759 GN=EhRAB7D PE=3 SV=1	0.012	0.833	5	1	1	1	204	22.9	5.2	0	1	0	
Medium	A0A5K1VPR3	Ribosomal protein L13 putative OS=Entamoeba histolytica OX=5759 GN=CL6EHI_017700 PE=2 SV=1	0.012	0.832	3	1	1	1	205	23.1	10.05	0	1	0	
Medium	A0A175JEG1	Rho guanine nucleotide exchange factor putative OS=Entamoeba histolytica OX=5759 GN=CL6EHI_044760A PE=4 SV=1	0.012	0.829	4	1	1	1	245	28.3	6.23	0	1	0	
Medium	A0A175JHM2	non-specific serine/threonine protein kinase OS=Entamoeba histolytica OX=5759 GN=CL6EHI_155360 PE=3 SV=1	0.012	0.826	4	1	3	1	231	26.6	8.85	0	1	0	160424.1094
Medium	A0A5K1UTA3	T-complex protein 1 subunit gamma OS=Entamoeba histolytica OX=5759 GN=CL6EHI_158250 PE=3 SV=1	0.012	0.808	1	1	2	1	551	60.1	6.67	0	1	0	285102.125
Medium	A0A5K1VUX5	H(+)-transporting two-sector ATPase OS=Entamoeba histolytica OX=5759 GN=CL6EHI_043010 PE=3 SV=1	0.012	0.807	1	1	1	1	607	67	5.36	0	1	0	
Medium	A0A5K1UFH2	Rab-GAP TBC domain-containing protein	0.012	0.806	2	1	1	1	350	41.7	7.88	1.86	1	0	

		OS=Entamoeba histolytica OX=5759 GN=CL6EHI_069260 PE=4 SV=1													
Medium	A0A5K1UHZ0	Uncharacterized protein OS=Entamoeba histolytica OX=5759 GN=CL6EHI_092620 PE=4 SV=1	0.012	0.802	2	1	1	1	415	46.8	5.6	0	1	0	
Medium	A0A175JTL8	Pumilio family RNA-binding protein OS=Entamoeba histolytica OX=5759 GN=CL6EHI_026330 PE=4 SV=1	0.016	0.798	2	1	1	1	526	61.3	4.82	0	1	0	
Medium	A0A5K1VEQ5	Glycerophosphoryl diester phosphodiesterase putative OS=Entamoeba histolytica OX=5759 GN=CL6EHI_068320 PE=4 SV=1	0.016	0.798	2	1	1	1	396	45.2	7.47	0	1	0	61739.22266
Medium	A0A5K1U3A8	small monomeric GTPase OS=Entamoeba histolytica OX=5759 GN=CL6EHI_190440 PE=4 SV=1	0.016	0.79	6	1	1	1	198	22.1	5.3	0	1	0	
Medium	A0A5K1ULP2	Cytoskeletal protein Sojo OS=Entamoeba histolytica OX=5759 GN=CL6EHI_086030 PE=4 SV=1	0.016	0.784	1	1	1	1	629	75	9.92	0	1	0	110859.2188
Medium	A0A175JFG2	Uncharacterized protein OS=Entamoeba histolytica OX=5759 GN=CL6EHI_178970 PE=4 SV=1	0.016	0.783	1	1	1	1	578	68.8	9.32	1.69	1	0	
Medium	A0A5K1VH68	Uncharacterized protein OS=Entamoeba histolytica OX=5759 GN=CL6EHI_035470 PE=4 SV=1	0.016	0.782	2	1	3	1	283	33.4	5.21	0	1	0	1671117.75

Medium	A0A5K1UPV6	GDP-Man:Man(3)GlcNAc(2)-PP-Dol alpha-1,2-mannosyltransferase OS=Entamoeba histolytica OX=5759 GN=CL6EHI_093950 PE=3 SV=1	0.017	0.777	2	1	1	1	445	50.8	8.37	0	1	0	280922.4688
Medium	A0A5K1U1J4	NTF2 domain-containing protein OS=Entamoeba histolytica OX=5759 GN=CL6EHI_080900 PE=3 SV=1	0.017	0.773	2	1	1	1	449	51.7	7.69	0	1	0	118123.8516
Medium	A0A5K1U953	Mic1 domain-containing protein OS=Entamoeba histolytica OX=5759 GN=CL6EHI_126970 PE=4 SV=1	0.017	0.773	1	1	1	1	917	106.2	6.16	0	1	0	3292105.75
Medium	A0A5K1UXP4	SPRY domain-containing protein OS=Entamoeba histolytica OX=5759 GN=CL6EHI_166970 PE=4 SV=1	0.017	0.767	4	1	1	1	337	39.4	4.93	0	1	0	
Medium	A0A060N1E3	Ribonuclease putative OS=Entamoeba histolytica OX=5759 GN=CL6EHI_156310 PE=2 SV=1	0.017	0.767	3	1	1	1	251	27.8	6.9	0	1	0	
Medium	A0A5K1USG3	Uncharacterized protein OS=Entamoeba histolytica OX=5759 GN=CL6EHI_004880 PE=3 SV=1	0.017	0.763	3	1	1	1	245	28.3	5.64	0	1	0	
Medium	A0A5K1VS95	Polyribonucleotide 5'-hydroxyl-kinase Clp1 P-loop domain-containing protein OS=Entamoeba histolytica OX=5759 GN=CL6EHI_011190 PE=3 SV=1	0.018	0.763	2	1	1	1	477	54	7.21	0	1	0	130863.7813

Medium	A0A5K1V6T4	Ribosomal RNA-processing protein 42 OS=Entamoeba histolytica OX=5759 GN=CL6EHI_092720 PE=3 SV=1	0.018	0.762	5	1	1	1	232	26.5	5.77	0	1	0	103835.9609
Medium	A0A5K1UE30	40S ribosomal protein s23 putative OS=Entamoeba histolytica OX=5759 GN=CL6EHI_020310 PE=3 SV=1	0.02	0.748	5	1	1	1	141	15.7	10.67	0	1	0	
Medium	A0A5K1TZU8	Uncharacterized protein OS=Entamoeba histolytica OX=5759 GN=CL6EHI_008120 PE=4 SV=1	0.022	0.746	2	1	1	1	329	36.8	4.93	0	1	0	108727.8672
Medium	A0A5K1U160	Proteasome subunit alpha type OS=Entamoeba histolytica OX=5759 GN=CL6EHI_163650 PE=3 SV=1	0.022	0.745	8	1	1	1	245	27.5	6.81	0	1	0	
Medium	A0A5K1VV18	adenosine deaminase OS=Entamoeba histolytica OX=5759 GN=CL6EHI_014320 PE=3 SV=1	0.022	0.74	2	1	1	1	348	39.3	5.57	0	1	0	1690584.125
